# Auditory evoked potential audiometry in fish

**DOI:** 10.1007/s11160-012-9297-z

**Published:** 2013-01-18

**Authors:** Friedrich Ladich, Richard R. Fay

**Affiliations:** 1 Department of Behavioural Biology, University of Vienna, Althanstrasse 14, 1090 Vienna, Austria; 2 Marine Laboratory, Woods Hole, MA 02543 USA; 3 179 Woods Hole Rd., Falmouth, MA 02540 USA

**Keywords:** AEP, Hearing, Sound pressure level, Particle acceleration levels, Thresholds, Noise, Ontogeny, Communication

## Abstract

A recent survey lists more than 100 papers utilizing the auditory evoked potential (AEP) recording technique for studying hearing in fishes. More than 95 % of these AEP-studies were published after Kenyon et al. introduced a non-invasive electrophysiological approach in 1998 allowing rapid evaluation of hearing and repeated testing of animals. First, our review compares AEP hearing thresholds to behaviorally gained thresholds. Second, baseline hearing abilities are described and compared in 111 fish species out of 51 families. Following this, studies investigating the functional significance of various accessory hearing structures (Weberian ossicles, swim bladder, otic bladders) by eliminating these morphological structures in various ways are dealt with. Furthermore, studies on the ontogenetic development of hearing are summarized. The AEP-technique was frequently used to study the effects of high sound/noise levels on hearing in particular by measuring the temporary threshold shifts after exposure to various noise types (white noise, pure tones and anthropogenic noises). In addition, the hearing thresholds were determined in the presence of noise (white, ambient, ship noise) in several studies, a phenomenon termed masking. Various ecological (e.g., temperature, cave dwelling), genetic (e.g., albinism), methodical (e.g., ototoxic drugs, threshold criteria, speaker choice) and behavioral (e.g., dominance, reproductive status) factors potentially influencing hearing were investigated. Finally, the technique was successfully utilized to study acoustic communication by comparing hearing curves with sound spectra either under quiet conditions or in the presence of noise, by analyzing the temporal resolution ability of the auditory system and the detection of temporal, spectral and amplitude characteristics of conspecific vocalizations.

## Introduction

In the modern era, interest in the questions of hearing by fishes began in 1903 (Parker 1903) and reached a peak in its first phase with the work of von Frisch (1938) and his students (e.g., von Frisch and Stetter 1932; von Frisch and Dijkgraaf 1935). The interest stemmed primarily from the questions of how the ears of fishes, lacking a basilar membrane, functioned in hearing, sound source localization, and in frequency analysis. Interest peaked again in the 1960s and 1970s (e.g., Tavolga and Wodinsky 1963; Enger 1966; Fay 1969; Popper 1970; Chapman and Johnstone 1974; Hawkins and Johnstone 1978). At that time, most investigators used behavior or conditioning techniques combined with psychophysical paradigms as the ideal way to investigate the sense of hearing in animals (reviewed in Fay 1988). In more recent years, behavioral techniques have declined in popularity due, for example, to the time required to determine a complete audiogram, the interest in rapid measures applicable to studies of temporary threshold shift, and in longitudinal studies on development. For these and other reasons, electrophysiological methods have become more popular than behavioral studies.

Invasive electrophysiological methods have included recording of auditory end organs (microphonic potentials), eighth nerve fibers, and neurons within the auditory brainstem and higher centers in auditory pathway (e.g., Furukawa and Ishii 1967; Fay and Popper 1974; Sand 1974; Fay and Ream 1986; Edds-Walton and Fay 2009). Invasive surgery generally precludes using an individual animal repeatedly. Non-invasive recordings of auditory evoked potentials [auditory evoked potentials (AEP) or auditory brainstem response (ABR)] in fish were first suggested by Bullock (1981) and Corwin (1981). Corwin et al. (1982) showed in two elasmobranchs and several bony fishes that AEPs can be recorded using cutaneous electrodes non-invasively attached to the head. In 1998 Kenyon et al. described a technical approach which allowed the measurement of complete audiograms within a short time period (4 h) based on the recording of AEPs from the skull surface. They demonstrated that the AEP-technique resulted in audiograms similar in shape to audiograms gained by behavioral methods in the goldfish *Carassius auratus* and the oscar *Astronotus ocellatus*.

Approximately 100 papers on more than 100 species have been published using the method inspired by Kenyon et al. (1998) on AEP. These papers range in focus from simple descriptions of auditory response to studies investigating the development of hearing, effects of noise on hearing, and the determination of peripheral sound conduction pathways and other fundamental hearing mechanisms in fishes. There is considerable diversity in auditory structures and functions among the more than 30,000 extant fish species. The new literature on AEP in fishes has helped to reveal some of the dimensions of this diversity by greatly expanding the number of species investigated.

The aim of our review is to present and compare results of these many recent experiments, and to help evaluate the use of AEP techniques to investigate auditory function in fishes.

## Behavioral and electrophysiological measures of hearing function

Hearing is generally defined as the act of perceiving sound, a sensory function that involves the entire organism’s behavior. This behavioral “act of perceiving” can only be measured using behavioral methods. We believe that behavioral studies of hearing have a face validity that AEP measures lack and that AEP audiograms, while popular and increasingly used, require comparison with behavioral audiograms wherever possible to help establish their validity as a possible description of a species characteristic (see “Behavioral and AEP thresholds in the goldfish *Carassius auratus*, Behavioral and AEP thresholds for other fish species” sections). Although behavioral and AEP audiograms are independent measures of auditory thresholds, we compare here the two measures to investigate the hypothesis that behavioral thresholds can be estimated from AEP measures.

Various behavioral techniques have been used in conjunction with psychophysical methods, including classical (Pavlovian) conditioning (e.g., Fay and MacKinnon 1969), operant conditioning (e.g., Yan and Popper 1991), and instrumental avoidance conditioning (e.g., Tavolga and Wodinsky 1963).

Electrophysiological measurements in hearing focus on a subset (sensory and neural) of the many functional elements that normally determine behavior, and as such comprise an incomplete description of hearing. But how “incomplete” is the definition of hearing using these methods? What can we know about hearing, and what can’t we know using electrophysiological methods? It is certain that a physiological response is not equivalent to “an act of perceiving” sound. However, it seems likely that a physiological response from the auditory brainstem or whole auditory pathway (inner ear up to the midbrain or forebrain) could stand in for, or predict, hearing under some circumstances, such measuring the Auditory Brainstem Response (ABR) in the screening for brainstem abnormalities and consequent hearing impairment in newborn infants (Starr et al. 1977).

There are two fundamental aspects of the description of an organism’s hearing capacities: the detectable range of frequencies (bandwidth), and absolute sensitivity (the lowest detectable stimulus level). We assume that electrophysiological methods can be used to reasonably estimate the frequency range of hearing (what sound frequencies produce detectable responses?). This is based on our belief that significant responses from some early elements of the auditory nervous system strongly imply that they could be used by the brain to signal the presence of a sound and produce a hearing sensation. This is not certain, but it is reasonable to believe.

The question of sensitivity, or the meanings of behavioral hearing thresholds versus those of electrophysiological thresholds, is much more problematic. Both behavioral and electrophysiological thresholds are properly defined statistically in terms of probabilities. However, there is no present theory of how these two very different types of thresholds relate to one another. Psychophysical thresholds have been studied for over 100 years, and the various complex factors that determine the statistical nature of the thresholds are relatively well understood (e.g., Green and Swets 1966). Electrophysiological thresholds have often been defined as a voltage response that is discernable with respect to the various sources of noise in the voltage waveform or spectrum, primarily by visual inspection. These measures can be objective and quantitative in every sense, but it is unknown (and perhaps unknowable) what the analytic relationship is between a given voltage recorded from the brainstem and a behavioral detection threshold. Therefore, the correspondence or correlation between these two independent quantities is the only means by which the electrophysiological thresholds can be evaluated as estimates of hearing thresholds measured behaviorally. One major purpose of this review is to determine what these correspondences (and variabilities) are, when both measures are available for a given species. However, we are well aware that this relationship may be species specific and dependent on many factors such as electrical and acoustic noise levels, electrode locations on the skull, and the acoustic characteristics of the test environment.

While electrophysiological and psychophysical thresholds are independent measures of auditory thresholds or auditory response, non-invasive electrophysiological measures have utility, both in within-species or individuals and in between-species investigations of hearing function. They are far more rapid and easier to accomplish compared with behavioral measures, and yet make sequential measurements on then same individuals possible. So, for example, they are ideal for use in experiments involving surgical manipulations of accessory hearing structures (e.g., swim bladders, Weberian ossicles), or in longitudinal studies of hearing development where the equality of behavioral and electrophysiological thresholds need not be assumed. In a limited way, they are also useful for between-species comparisons of sound thresholds and relative descriptions of the frequency range of auditory response (i.e., that one species has a wider hearing bandwidth or different best frequency of response than another). For these and other reasons, AEP audiometry in fishes has been popular in the literature since 1998, and has lead to renewed interest in the questions of hearing by fishes. As long as it is recognized that electrophysiological and behavioral methods do not necessarily lead to the same results, and that they are not ultimately equivalent or interchangeable descriptions of the auditory response, AEP studies on fishes should continue to grow in popularity and continue to answer fundamental questions about hearing in fishes. One of the goals of this review is to illustrate and evaluate how the AEP studies since 1998 have contributed to this rapidly growing field.

In summarizing this field of research, we note that it is presently controversial whether fishes having swim bladders without an obvious connection between the swim bladder and ears (otophysic connections) detect sound pressure or can only respond to acoustic particle motion. In most cases, critical experiments to confirm pressure sensitivity have not been done in fishes without special otophysic adaptations, and it is a matter of opinion whether or not the mere presence of a swim bladder confers sound pressure sensitivity (see Popper and Fay 2011). The Atlantic cod *Gadus morhua* (Chapman and Hawkins 1973) and the European eel *Anguilla anguilla* (Jerko et al. 1989) have been shown to be sound pressure sensitive at frequencies above about 100 Hz, but an otophysic connection has not been observed in these species. The Atlantic salmon *Salmo salar* (Hawkins and Johnstone 1978) and the dusky damselfish *Stegastes* *adustus* (formerly *Eupomacentrus dorsopunicans*) (Myrberg and Spires 1980) have been shown to be pressure-sensitive at the higher frequencies, but sensitive to particle acceleration at the lowest frequencies. The majority of species, however, have not been analyzed in this respect. For most species, other than the otophysi and others having a confirmed otophysic connection, auditory thresholds should be measured in terms of particle motion (either displacement, velocity, or acceleration) and sound pressure in order to help determine what acoustic quantity is most appropriate.

In typical experimental lab tanks, the ratio between sound pressure and particle motion amplitudes (impedance) is likely to be different from normal habitats (particle motion will be higher than in the species’ normal habitat). Because the relationship between sound pressure and particle motion in typical test tanks cannot be predicted in most cases sound pressure and particle acceleration levels should be measured at the same time. It is possible and likely that many unspecialized fishes detect particle acceleration in their natural habitat, not sound pressure, and audiometric data measured in sound pressure terms may not be appropriate.

### Behavioral and AEP thresholds in the goldfish *Carassius auratus*

Among the more than 30,000 extant fish species, the goldfish has become by far the most popular species for studying hearing physiology. The goldfish adapts easily to various holding conditions and is therefore chosen very often by physiologists. It belongs to the otophysines a series of primarily freshwater fishes comprising the orders Cypriniformes (carps and minnows), Siluriformes (catfishes), Characiformes (characins) and Gymnotiformes (South American knifefishes) which possesses well developed hearing and sensitivity to sound pressure due to their Weberian apparatus that mechanically connects the swim bladder and inner ears (e.g., Weber 1820; Popper and Fay 1973, 1993).

In this paper, we first compare hearing curves gained using behavioral techniques and electrophysiological techniques separately. Secondly, we compare the results of both techniques. This should help to determine if and to what degree audiograms differ using both approaches. Possibly, some ‘factor’ might be identified which would help to predict behavioral hearing thresholds (and bandwidths) from electrophysiological thresholds. Comparisons among other species (see below) for which these data exist could tell us whether or not this hypothetical ‘factor’ is universal among fish, or is species-specific. The baseline hearing abilities of goldfish have been investigated by numerous investigators applying different behavioral (Enger 1966; Fay 1969; Jacobs and Tavolga 1967; Offutt 1968; Popper 1971; Weiss 1966) or electrophysiological (e.g., Amoser and Ladich 2003; Cordova and Braun 2007) techniques in different labs.

**Fig. 1:**
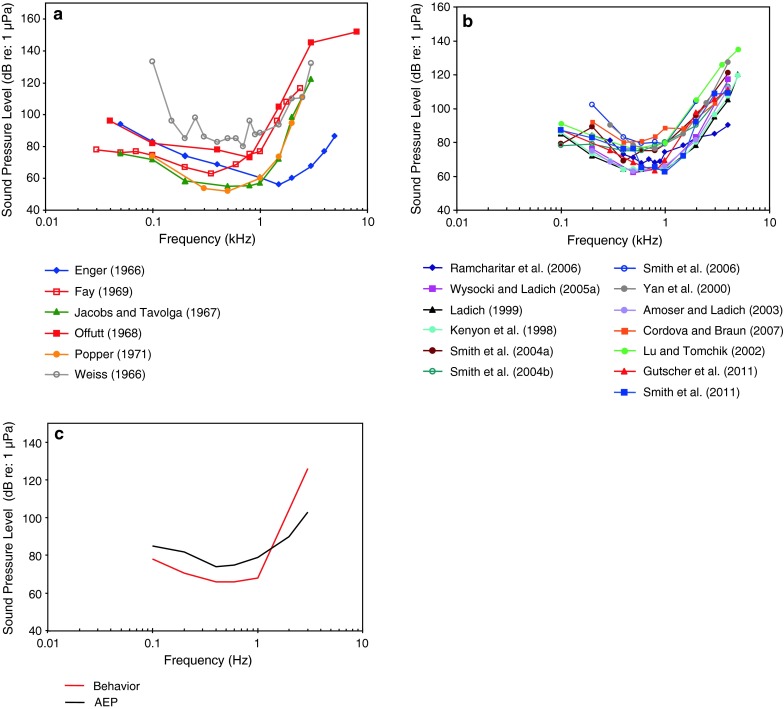
Comparisons of behavioral **a** and AEP **b** audiograms for goldfish (*Carassius auratus*). **c** Summaries derived from the median values of both behavioral and AEP data sets

The behavioral audiograms in Fig. 1a are quite diverse in threshold and bandwidth. Thresholds differ by as much as 60 dB at some frequencies. Best frequency of hearing is between 0.35 and 1.5 kHz, and thresholds at the best frequency varies between 52 and 80 dB re: 1 μPa. The conditioning and acoustic methods employed in these studies are also very diverse. Weiss (1966) used instrumental avoidance conditioning with two opposing sound projectors (Navy, J9) operating into a small plexiglas tank operating in a push–pull manner. This was done to create “a uniform sound field” and is unusual among all other studies on goldfish. It was criticised by Harris (comment in Weiss 1967) as possibly producing an “almost perfect near field.” Enger (1966) used “conditioned snapping for food” with an open top trough as a tank with a loudspeaker in air and a Navy J9 projector underwater, and got two different audiograms that only significantly differed from one another below about 1 kHz. Both Enger and Weiss believed that the lateral line system determined thresholds at the lower frequencies (below 200 Hz for Weiss 1966 and below 1 kHz for Enger 1966). Fay (1969) used classical respiratory conditioning with a loudspeaker in air operating into a cylindrical water tank through a closed air cavity above the water tank. Popper (1971) and Jacobs and Tavolga (1967) used instrumental avoidance conditioning with a loudspeaker in air, and Offutt (1968) used classical heart-rate conditioning with a Navy J9 projector. The various experiments on goldfish were all done at nominal “room temperature.” They may have used different strains of goldfish, but this was not noted or known by the investigators other than that Enger obtained goldfish in Norway and all the rest obtained them in the USA. Every study attempted to reduce ambient noise, but only in some cases were they reported. All studies used similar psychophysical methods (method of limits, and the staircase procedure). Therefore, the methodologies used in these studies varied considerably, but there is no particular correlation between the methods used and the resulting audiograms that we can make sense of, and thus no justifiable rationale for deciding which audiogram may be more valid.

The many AEP audiograms (Fig. 1b) are generally comparable in bandwidth and sensitivity with the behavioral audiograms but show somewhat less variation. The methods employed for the AEP audiograms are similarly diverse. Investigators used different speakers (air speakers above or beside subjects, vs. underwater speakers below or in front of subjects), fish positions (immediately below the water surface vs. 5–30 cm below the surface), different water temperatures, fish sizes, degrees of immobilization, different threshold criteria (visual comparison of AEP waveforms, waveform correlation coefficients, analysis of AEP spectra), different stimuli, different numbers of responses averaged (200–2,000), and perhaps different background noise levels. Best frequency varies from 0.3 to 0.8 kHz, and thresholds at best frequency vary between 63 and 84 dB.

The medians of all behavioral and all AEP data sets were calculated and shown in Fig. 1c. They reveal that median AEP thresholds are about 10 dB above behavioral thresholds up to 1 kHz, but are generally lower than behavioral thresholds above this frequency. This effect can partly be explained by the fact that it is difficult with the AEP technique to create short tone bursts at lower frequencies with good precision in the frequency domain. Short tone bursts with a greater rapidity of onset results in a greater efficacy at generating AEPs at higher frequencies (Silman and Silverman 1991; Kenyon et al. 1998).

It is also important to note that the signals used in AEP studies are short (about 20 ms in duration) while many of the behavioral studies used long duration signals (several seconds). Detection thresholds in behavioural studies have been shown to be higher when signal duration decreases in goldfish (Fay and Coombs 1983) and in Atlantic cod (Hawkins 1981), but in a study by Popper (1972a), no effect of signal duration was observed. So, signal duration may have contributed to the 10 dB differences observed between AEP and behavioral audiograms at frequencies below 1 kHz in goldfish. The lower AEP thresholds at higher frequencies contradict this assumption and cannot be explained by differences in stimuli length but by the greater rapidity of the stimulus onset.

Note that this effect of duration can be explained, at least in part, by central brain processing (Fay 1985) that may not be reflected in AEP measures.

### Behavioral and AEP thresholds for other fish species

There are only a few additional species which have been investigated in behavior and electrophysiology, and therefore our comparison is limited. These additional species are the little skate, *Raja erinaceus* (Casper et al. 2003), the common carp *Cyprinus carpio* (Popper 1972b; Köhler 1973; Amoser and Ladich 2005; Kojima et al. 2005), the oyster toadfish *Opsanus tau* (Fish and Offutt 1972; Yan et al. 2000), the European perch *Perca fluviatilis* (Wolff 1967; Amoser and Ladich 2005), the red sea bream *Pagrus major* (Kojima et al. 2010) and the oscar (Yan and Popper 1992; Kenyon et al. 1998).

#### Little skate *Raja erinacea*

**Fig. 2:**
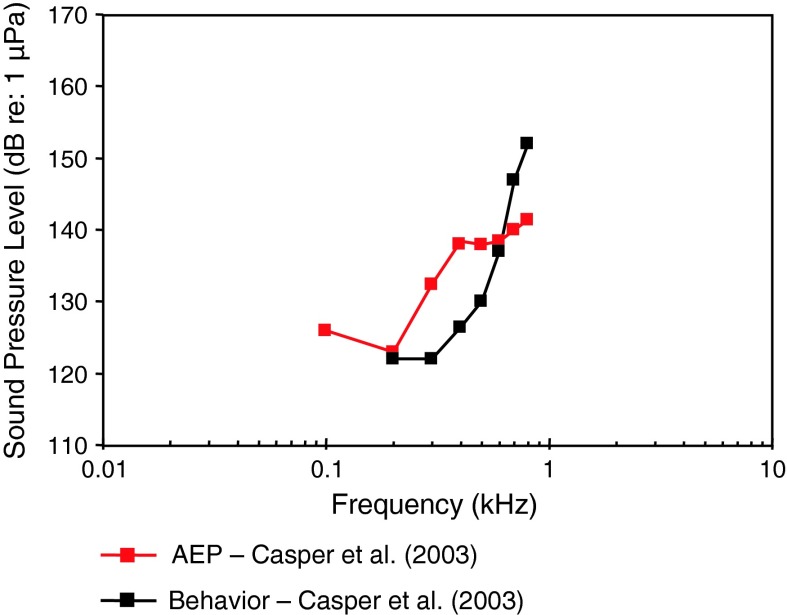
Audiograms for the little skate *Raja erinacea* determined behaviorally and using AEP measures. After Casper et al. (2003)

All elasmobranchs are sensitive to the impinging particle acceleration (and not sound pressure), because they lack a swim bladder, the structure that gives fish the capacity to detect sound pressure. For the little skate (family Rajidae) the audiograms are roughly similar, but with the AEP audiogram giving higher thresholds below and lower thresholds above 0.6 kHz than the behavioral audiogram (Casper et al. 2003). The skate’s frequency of best hearing is between 0.1 (AEP) and 0.2 kHz (behavior) (Fig. 2).

#### Common carp *Cyprinus carpio*

**Fig. 3:**
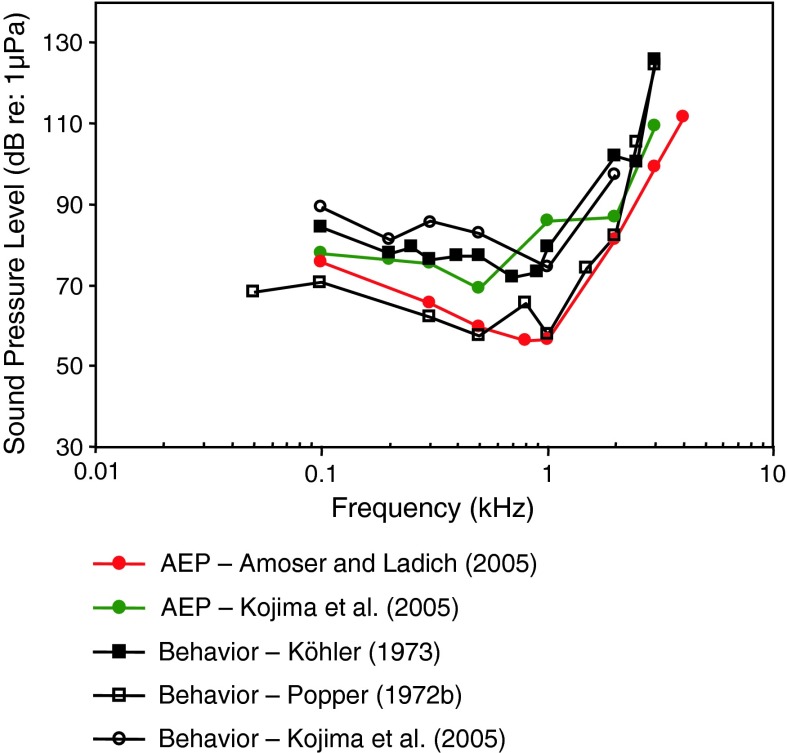
Behavioral and AEP thresholds for the carp *Cyprinus carpio*. Köhler (1973) investigated Japanese carps, commonly known as kois. All other studies used common carps. After Köhler (1973), Popper (1972b), Amoser and Ladich (2005) and Kojima et al. (2005)

For the common carp (family Cyprinidae), a species closely related to the goldfish, there is comparable variation threshold at best frequency among the behavioral and AEP data (Fig. 3). There is an excellent correspondence between behavioral thresholds of Popper (1972b) and the AEP thresholds of Amoser and Ladich (2005).

The two behavioral threshold estimates differ by as much as 20 dB at some frequencies, although the frequency of best hearing roughly agrees (between 0.5 and 1 kHz). The two AEP threshold estimates differ by about 25 dB at some frequencies, and the frequency of lowest thresholds vary somewhat between 0.5 and 0.8 kHz. Thus, for the carp, AEP thresholds by Amoser and Ladich (2005) are reasonable estimates of the behavioral thresholds.

Ideally, for comparative purposes behavioral and AEP-thresholds should be determined under the same acoustical conditions in the same lab. So far this was only done in the investigation by Kojima et al. (2005) in the carp. They used a heart rate (electrocardiogram, ECG) conditioning procedure including electric shocks to measure behavioral thresholds. In general differences between methodical approaches were small (Fig. 3). Interestingly, Kojima et al. (2005) got lower thresholds when using the AEP-techniques (except at 1 kHz) as compared to the behavioral (ECG) technique. This is in contrast to the comparison in goldfish using medians of all hearing curves (Fig. 1c).

#### Oyster toadfish *Opsanus tau*

**Fig. 4:**
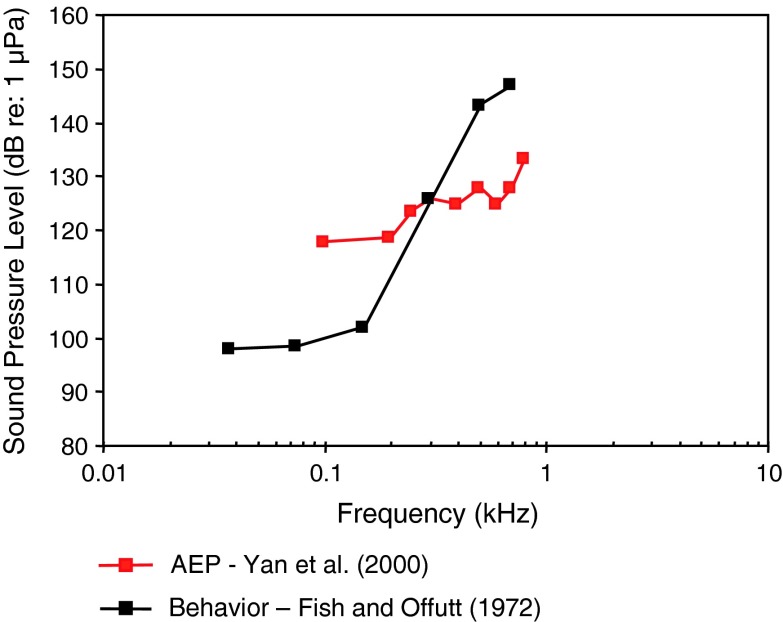
Behavioral (Fish and Offutt 1972) and AEP (Yan et al. 2000) audiogram for the oyster toadfish *Opsanus tau*

The oyster toadfish (family Batrachoididae) is a popular species for physiological studies, primarily of its directional hearing (e.g., Fay and Edds-Walton 1997). Figure 4 presents the behavioral (Fish and Offutt 1972) and AEP (Yan et al. 2000) audiogram estimates for the oyster toadfish. The correspondence between these curves is only general in that both indicate a very low-frequency response in which thresholds rise above 0.1 kHz. The AEP thresholds are about 20 dB higher at 0.1 kHz, but decline only gradually toward 0.8 kHz, and are about 15 dB below the behavioral thresholds at 0.8 kHz. Clearly, the AEP thresholds are below behavioral thresholds at the higher frequencies. For a comparison with other toadfish see the AEP audiogram of Lusitanian toadfish *Halobatrachus didactylus* (Fig. 17; Vasconcelos et al. 2007).

We note here that toadfish and many other species are unlike the goldfish and carp shown above in that they lack any peripheral specializations (even though they may have a swim bladder) that enhance hearing by sound pressure detection; toadfish are thought to detect sound through the otolith organ’s direct response to acoustic particle acceleration and not to sound pressure (e.g., Popper and Fay 2011), as is the case for elasmobranchs (see Fig. 2 above), and some other species.

#### European perch *Perca fluviatilus*

**Fig. 5:**
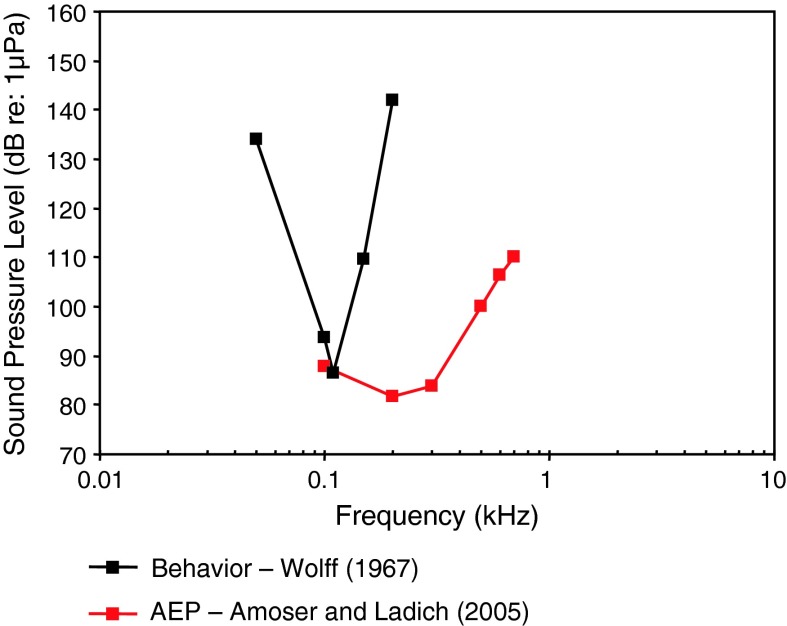
Audiograms for the European perch *Perca fluviatilis* determined using behavioral (Wolff 1967) and AEP paradigms (Amoser and Ladich 2005)

The European perch (family Percidae) is also not specialized for sound pressure hearing and is likely sensitive to particle acceleration. The behavioral audiogram by Wolff (1967) is unusual in that it shows a very narrowly tuned response at about 0.1 kHz (Fig. 5). The AEP audiogram for the European perch (Amoser and Ladich 2005) is more usual for unspecialized species than the behavioral audiogram.

#### Red sea bream *Pagrus major*

**Fig. 6:**
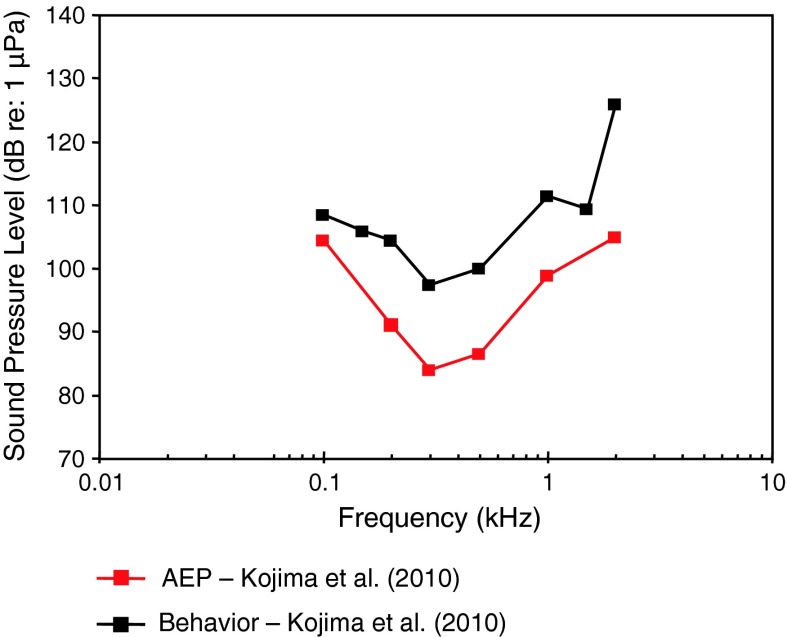
Audiograms for the Red sea bream *Pagrus major* determined using behavioral (heart rate conditioning) and AEP techniques. After Kojima et al. (2010)

Auditory sensitivity has been measured in the red sea bream (family Sparidae) by Kojima et al. (2010) utilizing a cardiac conditioning technique and an underwater speaker in the far field (7.7 m away from the animal), and using the AEP technique in a small tub using an air speaker. Both threshold curves were similar in shape with AEP showing lower thresholds (Fig. 6). Authors argue that the far field cardiac conditioning thresholds are pressure thresholds whereas the AEP thresholds were pressure and particle acceleration thresholds. This might explain partly why AEP thresholds are lower at 200–500 Hz. It seems unusual that the AEP gives lower thresholds at all frequencies (See “Behavioral and AEP thresholds in the goldfish *Carassius auratus*” section and Fig. 1c on the goldfish) but agrees with a similar observation of Kojima et al. (2005) in carps (Fig. 3).

#### Oscar *Astronotus ocellatus*

**Fig. 7:**
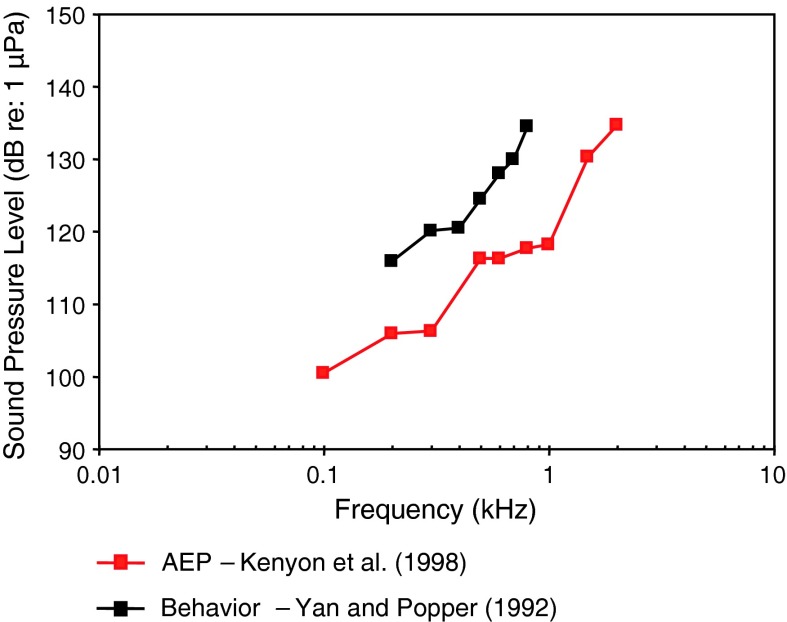
Audiograms for the oscar *Astronotus ocellatus* using behavioral (operant conditioning) (Yan and Popper 1992) and AEP (Kenyon et al. 1998) techniques

The oscar (family Cichlidae) is not specialized for sound pressure hearing, and thus is probably more properly described with respect to acoustic particle acceleration. Nevertheless, there is one behavioral (Yan and Popper 1992) and one AEP (Kenyon et al. 1998) sound pressure audiogram (Fig. 7). The AEP thresholds are well below behavioral thresholds, as is also the case for carps (Fig. 3) and red sea bream (Fig. 6). The behavioral study is the only example among hearing studies in fish to use operant conditioning for food reward. Oscars were trained to peck a paddle for food reward upon hearing a sound. The thresholds are unusually high, even for unspecialized fish. Yan and Popper (1992) mentioned that it was quite difficult to condition oscars to learn this response. Both estimates are similar in indicating that the oscar is a very low-frequency animal with relatively high thresholds and a frequency of best response at or below 0.1 kHz.

#### Summary and Conclusions about the relation between behavioral and AEP measures of hearing

Both behavioral and AEP measures of hearing exist for only seven fish species, even through about 60 species have been studied behaviorally, and approximately 100 species have been investigated using AEP measures. By far, the goldfish has been the most studied species. Behavioral and AEP audiograms for the goldfish show high variability among studies and laboratories. Thus, any one audiogram, whether behavioral or AEP, cannot be regarded as the proper definition of the species’ auditory capability with any certainty. However, within one laboratory and with the application of consistent techniques, the determined audiogram will still be useful and valid as a baseline measure of hearing against which the effects of manipulations of sound conducting structures, development, and acoustical manipulations can be evaluated (see next sections).

One generalization that arises from some of the comparisons above is that AEP measures tend to produce thresholds that are higher than the behavioral values at low frequencies, and produce lower thresholds than the behavioral thresholds at the higher frequencies. We can only be certain of this statistical tendency in the comparison between the median thresholds of six behavioral audiograms and the 10 AEP audiograms for the goldfish. Further evidence for this tendency comes from studies on the oyster toadfish (Fig. 4) but not from common carp (Fig. 2), the oscar (Fig. 7) and, possibly, the European perch (Fig. 5). Thus, it appears that the goal of discovering a “factor” for estimating behavioral thresholds from AEP measures is not possible. This is particularly the case when there is one AEP audiogram available for a given species (see “Systematic description of baseline AEP-audiograms” section below). In the absence of a behavioral audiogram, we recommend that such an AEP audiogram be accepted on its own terms as a reasonable estimate of auditory response for the species.

## Systematic description of baseline AEP-audiograms

**Fig. 8:**
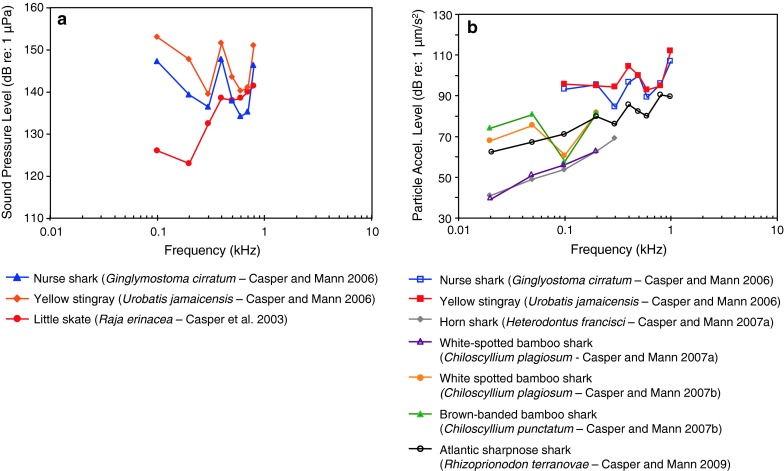
Audiograms for elasmobranchs studied using AEP technique. **a** SPL audiograms and **b** PAL audiograms. After Casper et al. (2003) and Casper and Mann (2006, 2007a, b, 2009)

**Fig. 9:**
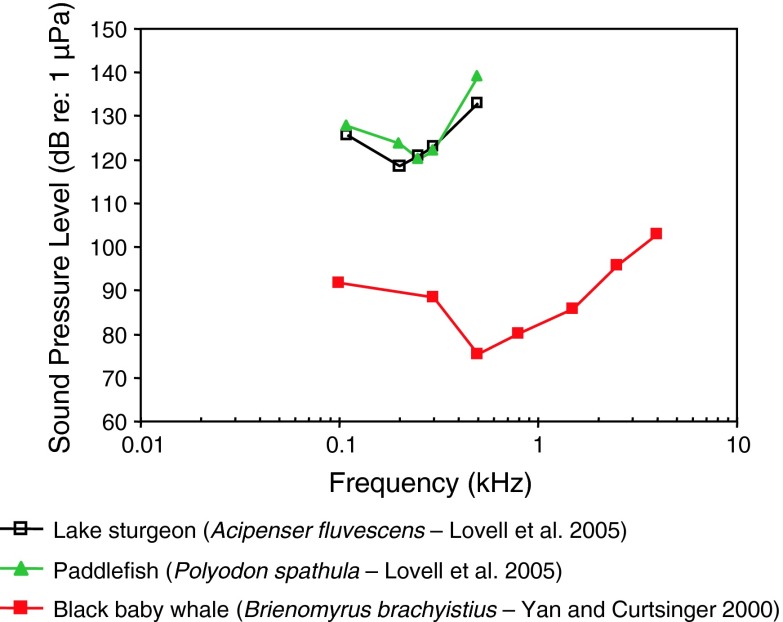
Audiograms for the lake sturgeon *Acipenser fluvescens*, the paddlefish *Polyodon spathula* and the black baby whale *Brienomyrus brachyistius*. After Yan and Curtsinger (2000) and Lovell et al. (2005)

**Fig. 10:**
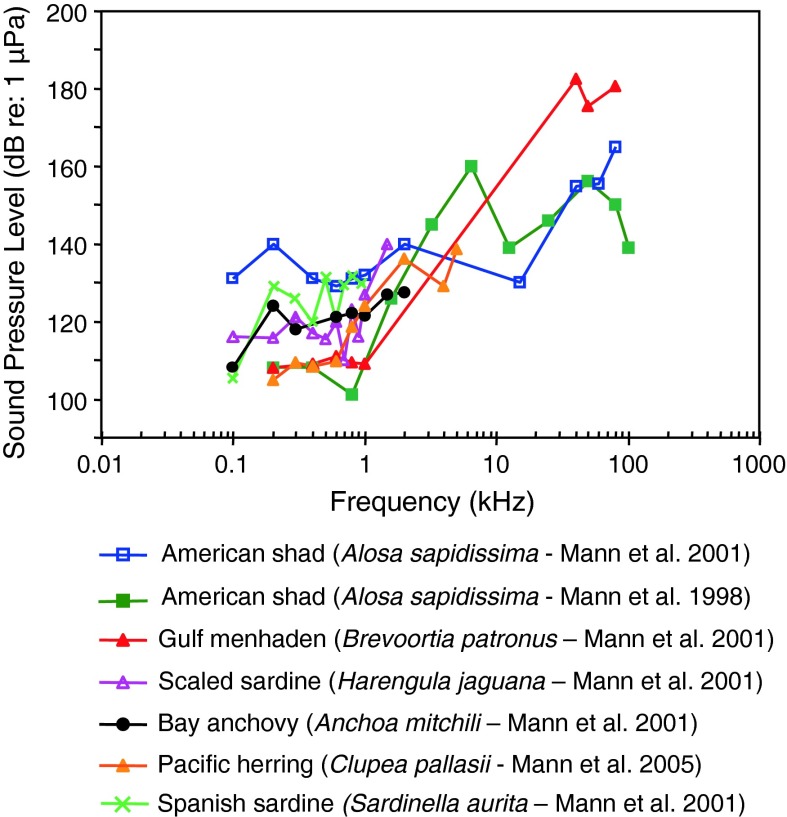
Audiograms for six representatives of the order Clupeiformes; After Mann et al. (1998, 2001, 2005)

**Fig. 11:**
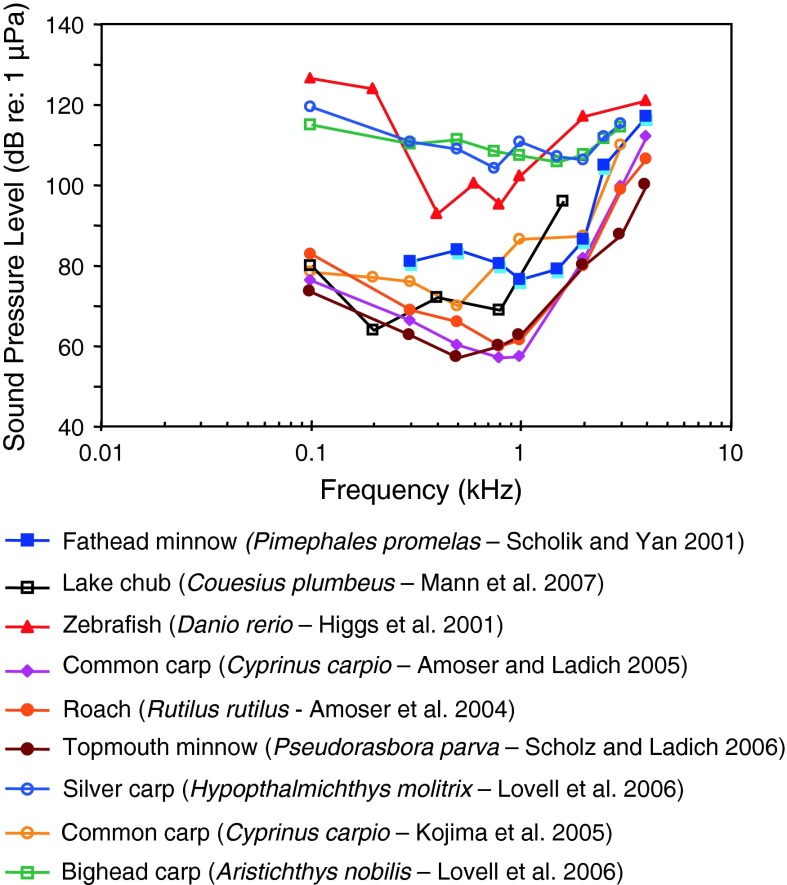
Audiograms for eight representatives of the family Cyprinidae. After Higgs et al. (2001), Scholik and Yan (2001), Amoser et al. (2004), Amoser and Ladich (2005), Kojima et al. (2005), Scholz and Ladich (2006), Lovell et al. (2006) and Mann et al. (2007)

**Fig. 12:**
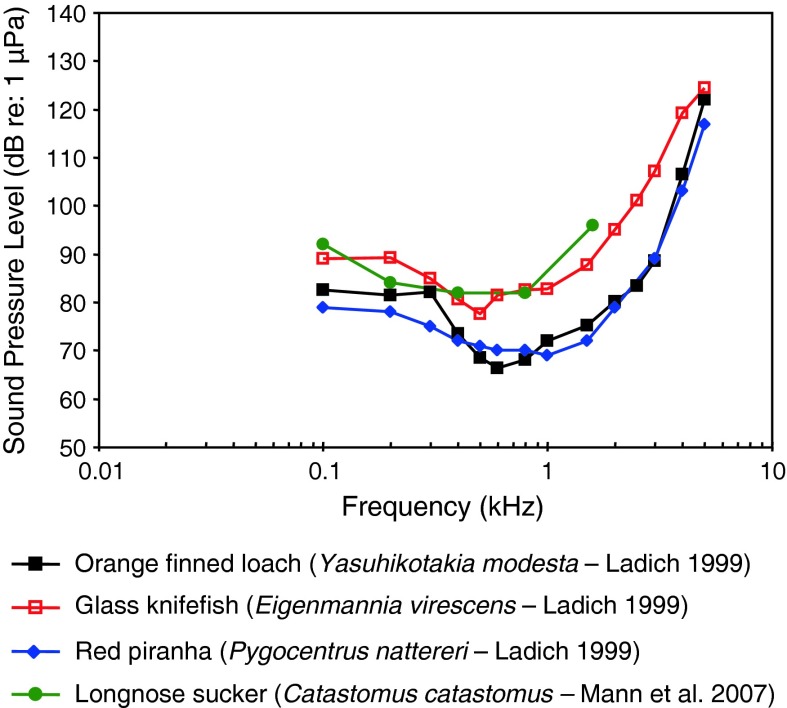
Audiograms for the longnose sucker *Castotomus catostomus*, orange finned loach *Yasuhikotakia modesta*, the red piranha *Pygocentrus* (formerly *Serrasalmus*) *nattereri* and the glass knifefish *Eigenmannia virescens* After Ladich (1999) and Mann et al. (2007)

**Fig. 13:**
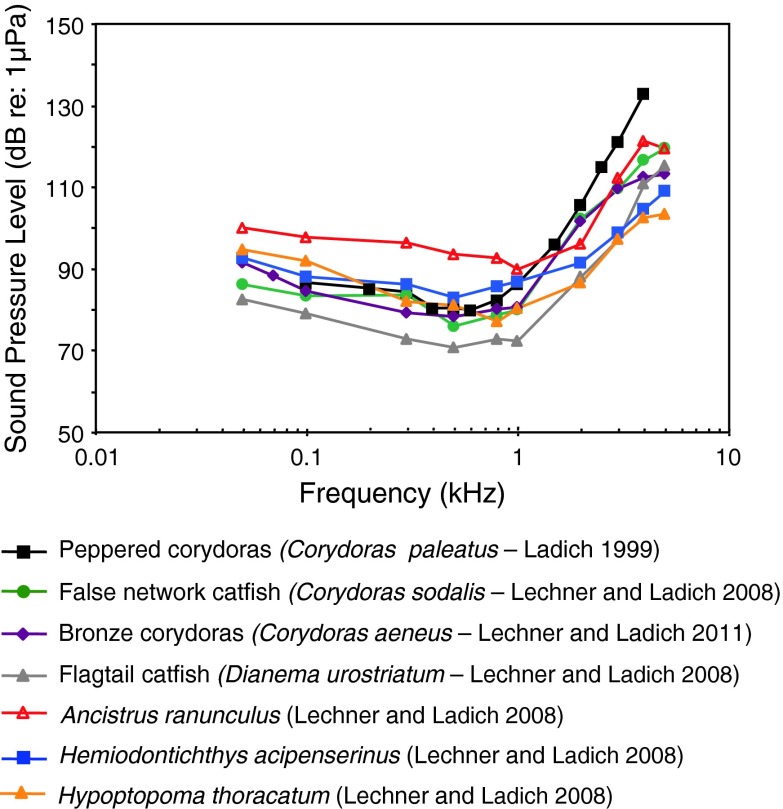
Audiograms of eight representatives of the catfish families Doradidae, Auchenipteridae, Pseudopimelodidae, Heptateridae, Malapteruridae, Mochokidae and Ictaluridae. After Ladich (1999), Lechner and Ladich (2008) and Wysocki et al. (2009b)

**Fig. 14:**
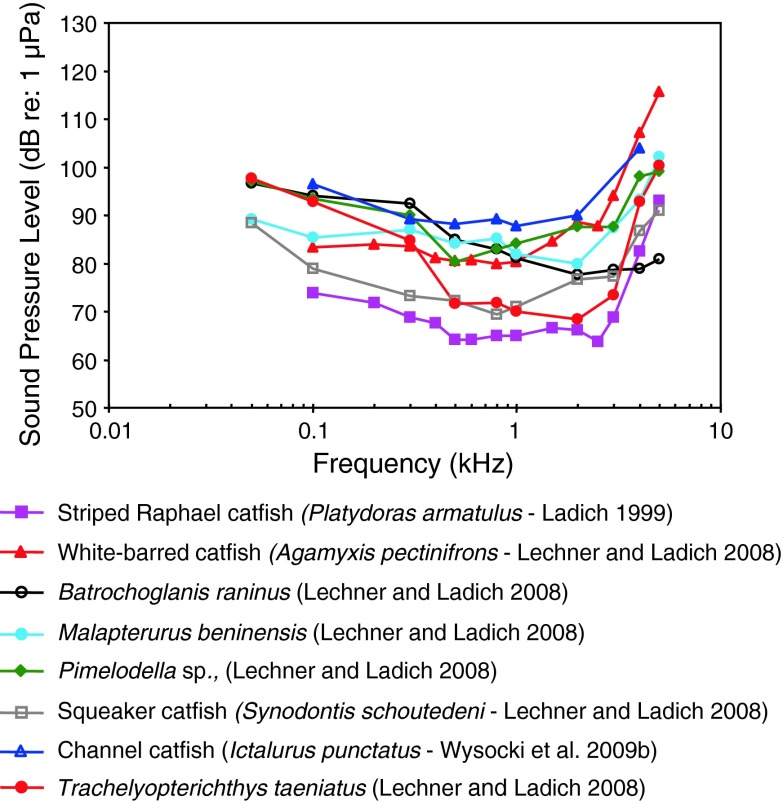
Audiograms of eight representatives of the catfish families Doradidae, Auchenipteridae, Pseudopimelodidae, Heptateridae, Malapteruridae, Mochokidae and Ictaluridae. After Ladich (1999), Lechner and Ladich (2008) and Wysocki et al. (2009b)

**Fig. 15:**
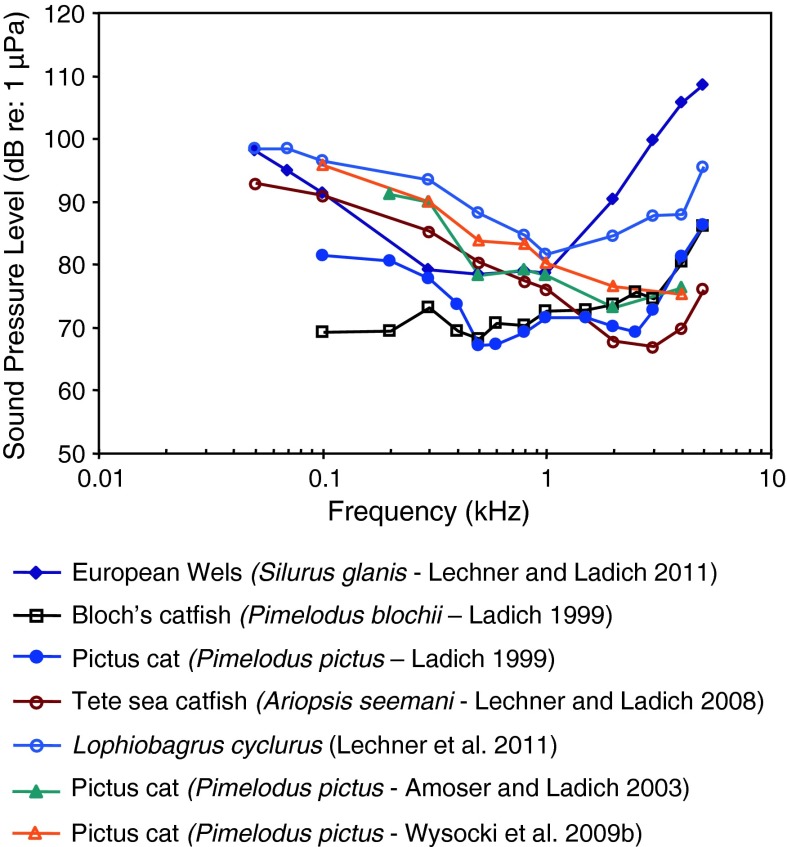
Audiograms for seven representatives of the catfish families Siluridae, Pimelodidae, Ariidae and Claroteidae. After Ladich (1999), Lechner and Ladich (2008, 2011), Wysocki et al. (2009b) and Lechner et al. (2011)

**Fig. 16:**
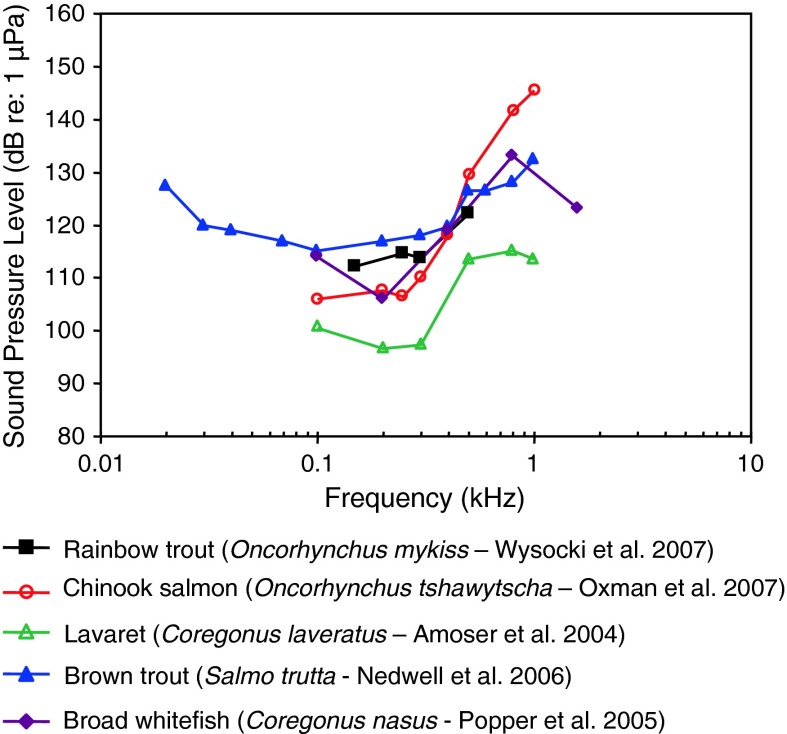
Audiograms for five representatives of the family Salmonidae. After Amoser et al. (2004), Popper et al. (2005), Nedwell et al. (2006), Oxman et al. (2007) and Wysocki et al. (2007)

**Fig. 17:**
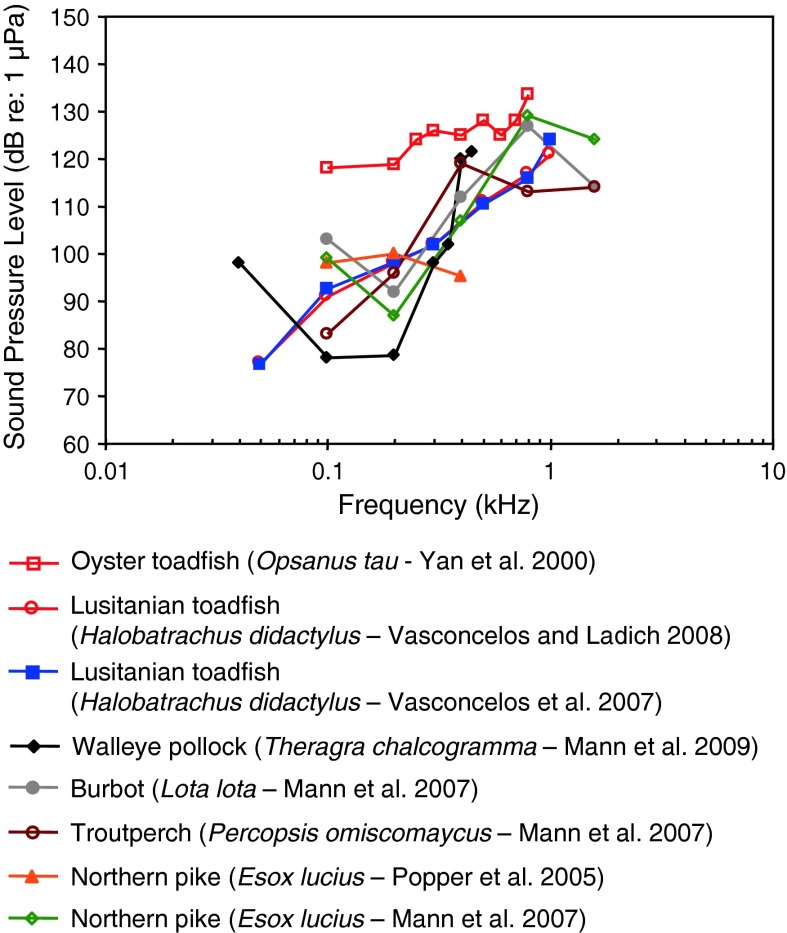
Audiograms for six representatives of the orders Esociformes, Percopsiformes, Batrachoidiformes and Gadiformes. After Yan et al. (2000), Popper et al. (2005), Vasconcelos et al. (2007), Mann et al. (2007, 2009) and Vasconcelos and Ladich (2008)

**Fig. 18:**
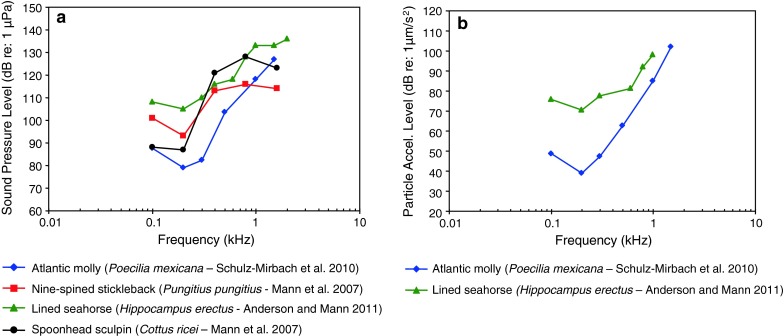
Audiograms of four representatives of the orders Cyprinodontiformes, Gasterosteiformes and Scorpaeniformes. **a** SPL audiograms and **b** PAL audiograms. After Mann et al. (2007), Schulz-Mirbach et al. (2010) and Anderson and Mann (2011)

**Fig. 19:**
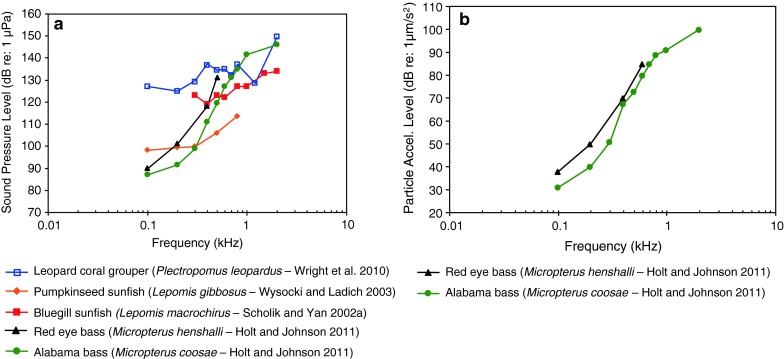
Audiograms for one representative of the family Serranidae and four of the family Centrarchidae. **a** SPL audiograms and **b** PAL audiograms. After Wright et al. (2010), Scholik and Yan (2002a), Wysocki and Ladich (2003) and Holt and Johnston (2011)

**Fig. 20:**
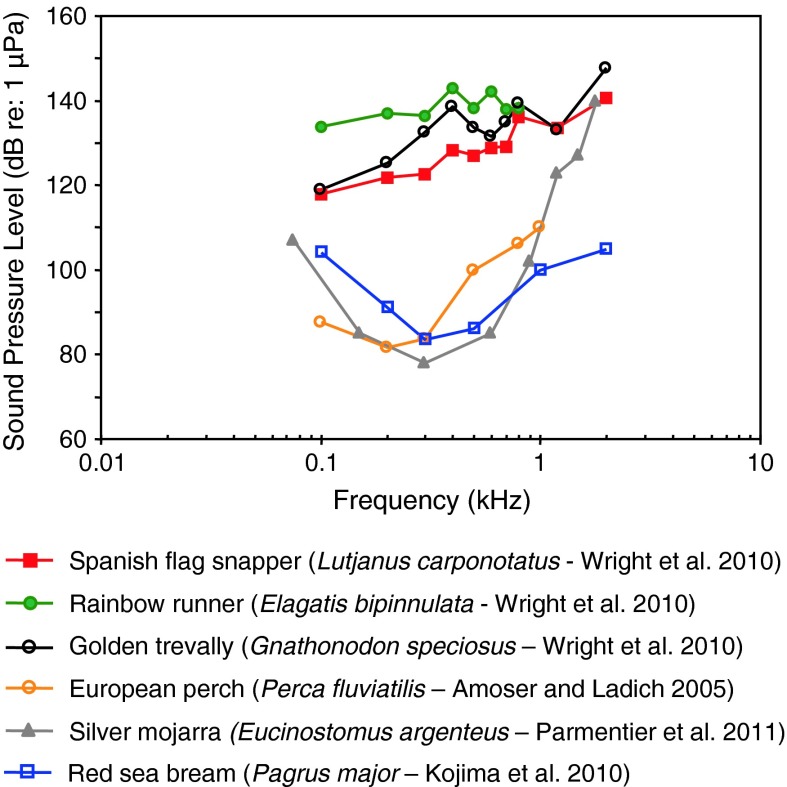
Audiograms for representatives of the perciform families Percidae, Carangidae, Lutjanidae, Gerreidae and Sparidae. After Amoser and Ladich (2005), Kojima et al. (2010), Wright et al. (2010) and Parmentier et al. (2011)

**Fig. 21:**
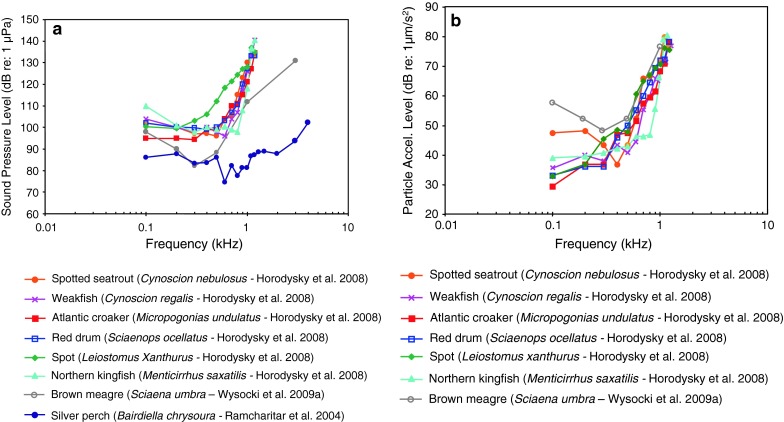
Audiograms for eight representatives of the perciform family Sciaenidae. **a** SPL audiograms and **b** PAL audiograms. After Ramcharitar et al. (2004), Horodysky et al. (2008), and Wysocki et al. (2009a)

**Fig. 22:**
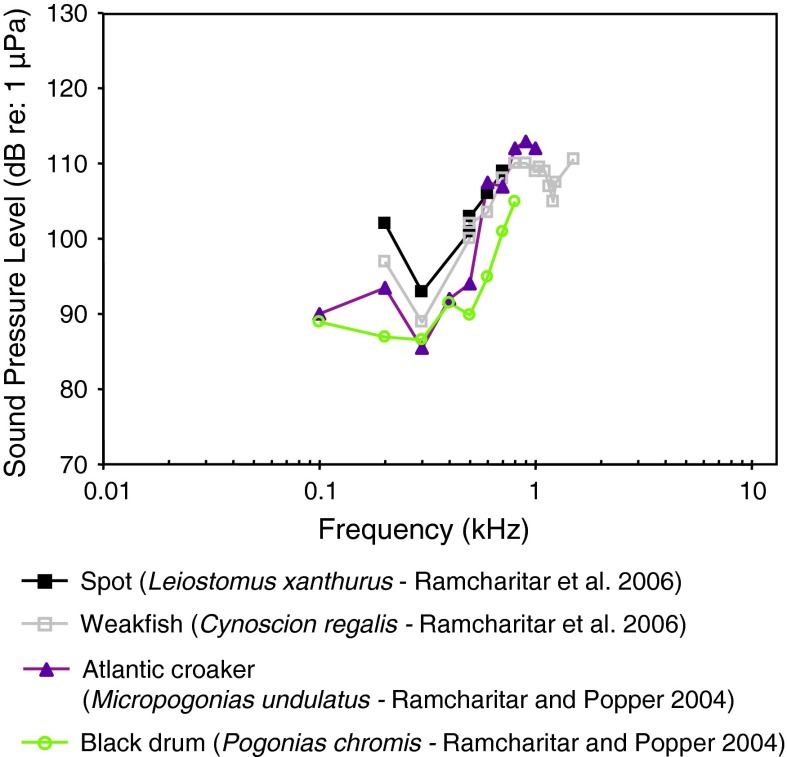
Audiograms for four representatives of the perciform family Sciaenidae. After Ramcharitar and Popper (2004) and Ramcharitar et al. (2006)

**Fig. 23:**
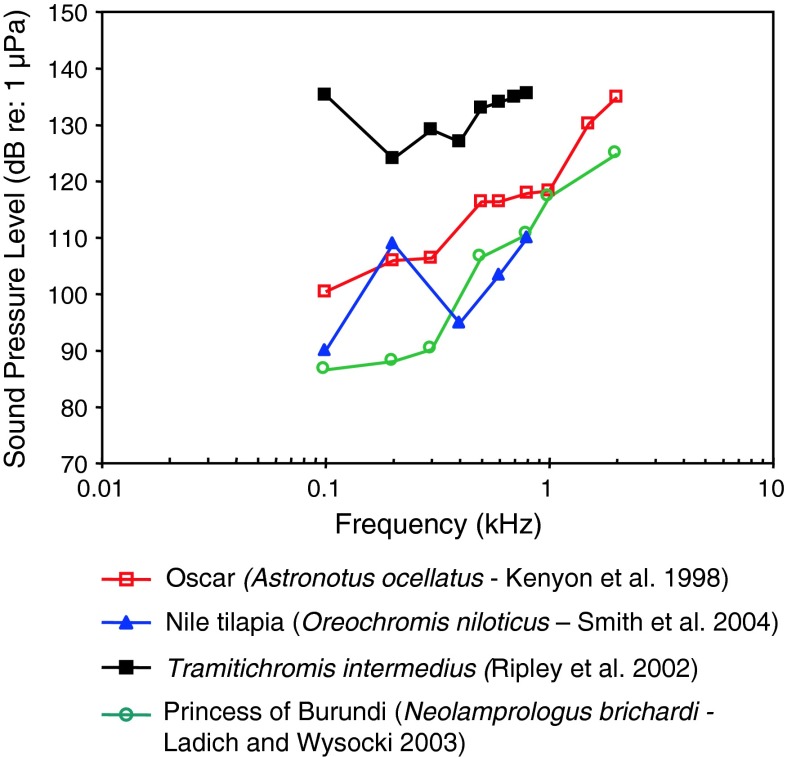
Audiograms for four representatives of the perciform family Cichlidae. After Kenyon et al. (1998), Ripley et al. (2002), Ladich and Wysocki (2003) and Smith et al. (2004b)

**Fig. 24:**
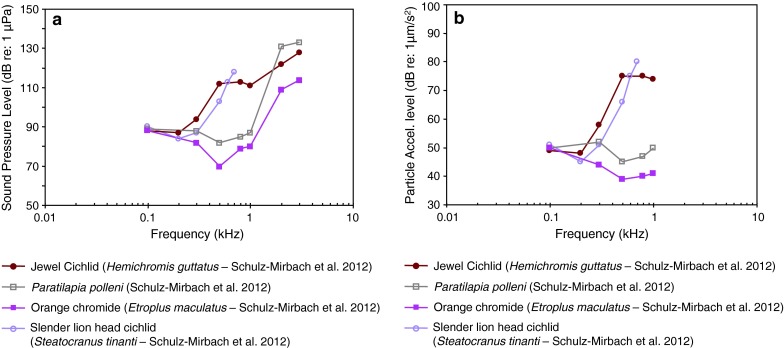
Audiograms of four representatives of the perciform family Cichlidae in which **a** SPL and **b** PAL audiograms have been determined. After Schulz-Mirbach et al. (2012)

**Fig. 25:**
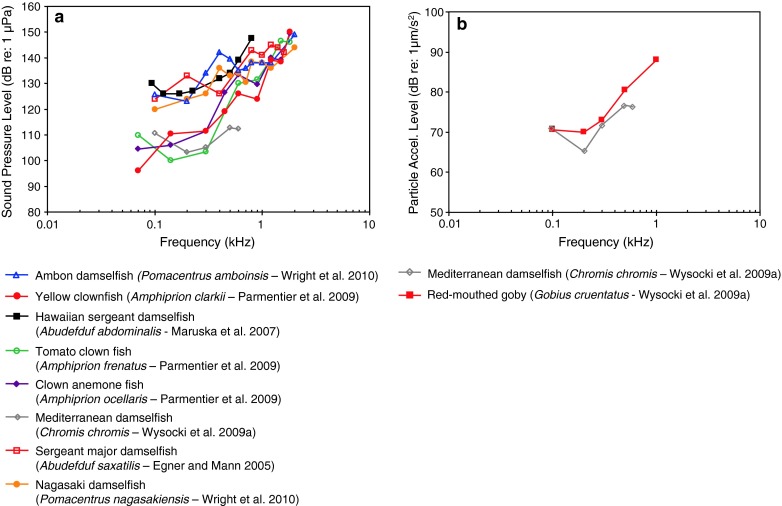
Audiograms for eight representatives of the perciform family Pomacentridae. **a** SPL audiograms and **b** PAL audiogram for the damselfish *Chromis chromis* and the red-mouthed goby *Gobius cruentatus*. After Egner and Mann (2005), Maruska et al. (2007), Wysocki et al. (2009a), Parmentier et al. (2009) and Wright et al. (2010)

**Fig. 26:**
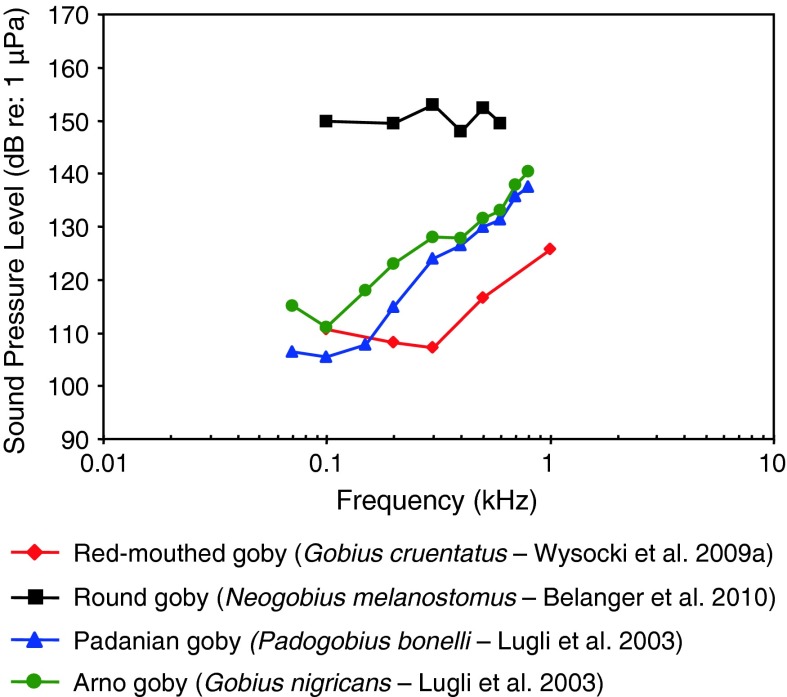
Audiograms for four representatives of the perciform family Gobiidae. SPL audiograms are shown. The PAL audiogram of the red-mouthed goby *Gobius cruentatus* is shown in Fig. 25b. After Lugli et al. (2003), Wysocki et al. (2009a) and Belanger et al. (2010)

**Fig. 27:**
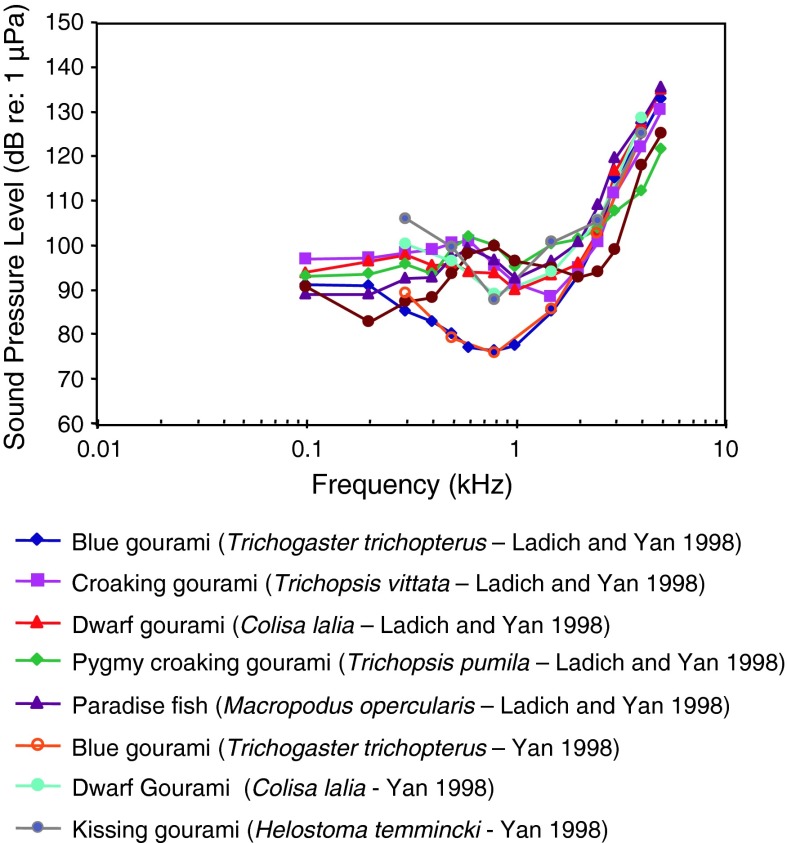
Audiograms for representatives of the closely related perciform families Osphronemidae and Helostomatidae (labyrinth fishes or gouramis). After Ladich and Yan (1998) and Yan (1998)

**Fig. 28:**
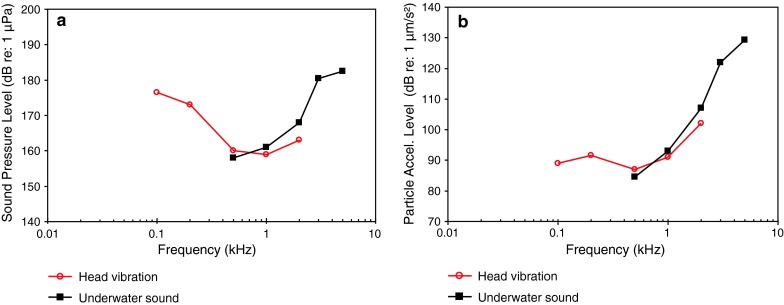
Audiograms for the African lungfish *Protopterus annectens*. **a** SPL audiogram and **b** PAL audiogram. After Christensen-Daalsgard et al. (2011)

AEP-audiograms of 110 fish species out of 22 orders out of 51 families have been published and are dealt with in this review (see Table 1; Figs. 8, 9, 10, 11, 12, 13, 14, 15, 16, 17, 18, 19, 20, 21, 22, 23, 24, 25, 26, 27, 28). We use the term baseline audiogram to indicate that animals have not been manipulated in any (known) way such as by prior noise exposure, eliminating accessory hearing structures etc. We grouped species and subsequently presented their audiograms according to their systematic relationship following the systematics by Nelson (2006) starting with cartilaginous fishes, followed by bony fishes and ending with lungfishes (see Table 1). In the majority of species (with a few exceptions) auditory thresholds have been described in terms of sound pressure level (SPL) and in a few cases only in terms of particle acceleration levels (PAL), and in some cases, in both. Seven cartilaginous species (5 shark species, 2 ray species¸ Fig. 8a, b) have been described in contrast to more than 100 bony fish species.

**Table 1:** Systematic overview over fish species where baseline hearing sensitivities have been described using the AEP recording technique

Order	Family	Genus species	Common name	Additional variable	Reference	Figures
Heterodontiformes	Heterodontidae	*Heterodontus fransisci*	Horn shark	PAL only	Caspar and Mann (2007a)	8b
Orectolobiformes	Hemiscyliidae	*Chiloscyllium plagiosum*	White-spotted bamboo shark	PAL only	Caspar and Mann (2007a, b)	8b
		*Chiloscyllium punctatum*	Brown-banded bamboo shark	PAL only	Caspar and Mann (2007b)	8b
Carcharhiniformes	Carcharhinidae	*Ginglymostoma cirratum*	Nurse shark	PAL	Casper and Mann (2006)	8a, b
		*Rhizoprionodon terraenovae*	Atlantic sharpnose shark	PAL	Caspar and Mann (2009)	8b
Rajiformes	Rajidae	*Raja erinacea*	Little skate	Food reward conditioning	Casper et al. (2003)	8a
Myliobatiformes	Urotrygonidae	*Urobatis jamaicensis*	Yellow stingray	PAL	Casper and Mann (2006)	8a, b
Acipenseriformes	Acipenseridae	*Acipenser fulvescens*	Lake sturgeon		Lovell et al. (2005)	9
	Polyodontidae	*Polyodon spathula*	Paddlefish		Lovell et al. (2005)	9
Osteoglossiformes	Mormyridae	*Brienomyrus brachyistius*	Black baby whale	Otic gasbladder deflation	Yan and Curtsinger (2000)	9
Clupeiformes	Engraulidae	*Anchoa mitchilli*	Bay anchovy		Mann et al. (2001)	10
	Clupeidae	*Brevoortia patronis*	Gulf menhaden		Mann et. al. (2001)	10
				Elimination experiment	Wilson et al. (2009)	
		*Alosa sapidissima*	American shad		Mann et al. (2001)	10
				Ontogeny	Higgs et al. (2004)	34
		*Clupea pallasii*	Pacific herring		Mann et al. (2005)	10
		*Harengula jaguana*	Scaled sardine		Mann et al. (2001)	10
		*Sardinella aurita*	Spanish sardine		Mann et al. (2001)	10
Cypriniformes	Cyprinidae	*Carassius auratus*	Goldfish		Kenyon et al. (1998)	1b
					Ladich (1999)	1b
				WN exposure, TTS	Amoser and Ladich (2003)	1b
				Temporal resolution	Wysocki and Ladich (2002)	
				Tripus extirpation	Ladich and Wysocki (2003)	
				WN exposure, TTS	Smith et al. (2004a)	1b
				WN exposure, TTS	Smith et al. (2004b)	1b
				WN exposure, TTS	Smith et al. (2006)	1b
				Pure tone exposure, TTS	Smith et al. (2011)	1b
					Ramcharitar et al. (2006)	1b
				WN masking	Wysocki and Ladich (2005a)	1b
				WN exposure, temporal resolution	Wysocki and Ladich (2005b)	
				Aquarium noise masking	Gutscher et al. (2011)	1b
				Evaluation of AEP method	Ladich and Wysocki (2009)	
				Anesthesia	Cordova and Braun (2007)	1b
				Effects of red-tide toxin	Lu and Tomchik (2002)	1b
				Evaluation of thresholds determination	Xiao and Braun (2008)	
				Swimbladder deflation	Yan et al. (2000)	1b
		*Cyprinus carpio*	Carp	Ambient noise masking	Amoser and Ladich (2005)	4, 11
				ECG	Kojima et al. (2005)	4, 11
		*Pseudorasbora parva*	Topmouth minnow	Lake noise masking, sounds	Scholz and Ladich (2006)	11
		*Danio rerio*	Zebrafish	Ontogeny	Higgs et al. (2001)	11
				Ontogeny	Higgs et al. (2003)	
		*Couesius plumbeus*	Lake chub		Mann et al. (2007)	11
				Seismic airgun exposure, TTS	Popper et al. (2005)	
		*Pimephales promelas*	Fathead minnow	WN exposure, TTS	Scholik and Yan (2001)	11
				Boat noise exposure, TTS	Scholik and Yan (2002a)	
		*Rutilus rutilus*	Roach	Swimbladder elimination	Laming and Morrow (1981)	
					Amoser et al. (2004)	11
		*Hypopthalmichthys molitrix*	Silver carp		Lovell et al. (2006)	11
		*Aristichthys nobilis*	Bighead carp		Lovell et al. (2006)	11
	Catostomidae	*Catostomus catostomus*	Longnose sucker		Mann et al. (2007)	12
	Cobitidae	*Yasuhikotakia modesta*	Orange finned loach	Vocalizations	Ladich (1999)	12
				Lateral trunk channel elimination	Kratochvil and Ladich (2000)	
Characiformes	Characidae	*Pygocentrus nattereri*	Red piranha	Vocalizations	Ladich (1999)	12
Siluriformes	Callichthyidae	*Corydoras paleatus*	Peppered corydoras	Vocalizations	Ladich (1999)	13
		*Corydoras sodalis*	False network catfish	Weberian ossicles	Lechner and Ladich (2008)	13
		*Corydoras aeneus*	Bronze corydoras	Albinism	Lechner and Ladich (2011)	13
		*Dianema urostriatum*	Flagtail catfish	Weberian ossicles	Lechner and Ladich (2008)	13
	Loricariidae	*Ancistrus ranunculus*		Weberian ossicles	Lechner and Ladich (2008)	13
		*Hemiodontichthys acipenserinus*		Weberian ossicles	Lechner and Ladich (2008)	13
		*Hypoptopoma thoracatum*		Weberian ossicles	Lechner and Ladich (2008)	13
	Pseudopimelodidae	*Batrochoglanis raninus*		Weberian ossicles	Lechner and Ladich (2008)	14
	Heptapteridae	*Pimelodella* sp.		Weberian ossicles	Lechner and Ladich (2008)	14
	Ictaluridae	*Ictalurus punctatus*	Channel catfish	Temperature	Wysocki et al. (2009b)	14
				MFAS exposure, TTS	Halvorsen et al. (2012)	
	Mochokidae	*Synodontis schoutedeni*	Squeaker catfish	Weberian ossicles	Lechner and Ladich (2008)	14
				Ontogeny, vocalizations	Lechner et al. (2010)	
	Doradidae	*Platydoras armatulus*	Striped Raphael catfish	Vocalizations	Ladich (1999)	14
				AEP to vocalizations	Wysocki and Ladich (2003)	
				WN masking	Wysocki and Ladich (2002a, b	
				Temperature, vocalizations	Papes and Ladich (2011)	
				Temporal resolution	Wysocki and Ladich (2002)	
		*Agamyxis pectinifrons*	Whitebarred catfish	Vocalization	Ladich (1999)	14
	Auchenipteridae	*Trachelyopterichthys taeniatus*	Striped woodcat	Weberian ossicles	Lechner and Ladich (2008)	14
	Siluridae	*Silurus glanis*	European wels	Albinism	Lechner and Ladich (2011)	15
	Malapteruridae	*Malapterurus beninensis*		Weberian ossicles	Lechner and Ladich (2008)	14
	Claroteidae	*Lophiobagrus cyclurus*	African bullhead	Ontogeny, Weberian ossicles	Lechner et al. (2011)	15
	Ariidae	*Ariopsis seemani*	Tete sea catfish	Weberian ossicles	Lechner and Ladich (2008)	15
	Pimelodidae	*Pimelodus blochii*	Bloch’s catfish	Vocalization	Ladich (1999)	15
		*Pimelodus pictus*	Pictus cat	Vocalization	Ladich (1999)	15
				AEP to vocalizations	Wysocki and Ladich (2003)	
				WN exposure, TTS	Amoser and Ladich (2003)	15
				Temperature	Wysocki et al. (2009b)	15
Gymnotiformes	Sternopygidae	*Eigenmannia virescens*	Glass knifefish		Ladich (1999)	12
Salmoniformes	Salmonidae	*Oncorhynchus mykiss*	Rainbow trout	Aquaculture noise masking, TTS	Wysocki et al. (2007)	16
				LFAS exposure, TTS	Popper et al. (2007)	
				MFAS exposure, TTS	Halvorsen et al. (2012)	
		*Oncorhynchus tshawytscha*	Chinook salmon	Otolith crystal structure	Oxman et al. (2007)	16
		*Coregonus nasus*	Broad whitefish	Seismic airgun exposure, TTS	Popper et al. (2005)	16
					Mann et al. (2007)	
		*Coregonus lavaretus*	Lavaret		Amoser et al. (2004)	16
		*Salmo truta*	Brown trout		Nedwell et al. (2006)	16
Esociformes	Esocidae	*Esox lucius*	Northern pike	Seismic airgun exposure, TTS	Popper et al. (2005)	17
					Mann et al. (2007)	17
Percopsiformes	Percopsidae	*Percopsis omiscomaycus*	Troutperch		Mann et al. (2007)	17
Gadiformes	Gadidae	*Lota lota*	Burbot		Mann et al. (2007)	17
		*Theragra chalcogramma*	Walleye pollock	Three age groups	Mann et al. (2009)	17
Batrachoidiformes	Batrachoididae	*Halobatrachus didactylus*	Lusitanian toadfish	Ship noise masking, vocalization	Vasconcelos and Ladich (2008)	17
				Ontogeny	Vasconcelos et al. (2007)	17
		*Opsanus tau*	Oyster toadfish	Swim bladder deflation	Yan et al. (2000)	17
Cyprinodontiformes	Poeciliidae	*Poecilia mexicana*	Atlantic molly	PAL, cave fish	Schulz-Mirbach et al. (2010)	18a, b
Gasterosteiformes	Gasterosteidae	*Pungitius pungitius*	Nine-spined stickelback		Mann et al. (2007)	18a
	Syngnathidae	*Hippocampus erectus*	Lined seahorse	PAL	Anderson and Mann (2011)	18a, b
Scorpaeniformes	Cottidae	*Cottus ricei*	Spoonhead sculpin		Mann et al. (2007)	18a
Perciformes	Serranidae	*Plectropomus leopardus*	Leopard coralgrouper	Settlement-stage larvae	Wright et al. (2010)	19a
	Centrarchidae	*Lepomis gibbosus*	Pumkinseed sunfish	WN masking	Wysocki and Ladich (2005a)	19a
				AEP to vocalizations	Wysocki and Ladich (2003)	
		*Micropterus coosae*	Red eye bass	PAL	Holt and Johnston (2011)	19a, b
		*Micropterus henshalli*	Alabama bass	PAL	Holt and Johnston (2011)	19a, b
		*Lepomis macrochirus*	Bluegill sunfish	Boat noise exposure, TTS	Scholik and Yan (2002a, b)	19a
	Percidae	*Perca fluviatilis*	European perch	Ambient noise masking	Amoser and Ladich (2005)	20
	Carangidae	*Gnathanodon speciosus*	Golden trevally	Settlement-stage larvae	Wright et al. (2010)	20
		*Elagatis bipinnulata*	Rainbow runner	Settlement-stage larvae	Wright et al. (2010)	20
	Lutjanidae	*Lutjanus carponotatus*	Spanish flag snapper	Settlement-stage larvae	Wright et al. (2010)	20
	Gerreidae	*Eucinostomus argenteus*	Silver mojarra	Swimbladder	Parmentier et al. (2011)	20
	Sparidae	*Pagrus major*	Red Sea bream	ECG	Kojima et al. (2010)	20
	Sciaenidae	*Cynoscion regalis*	Weakfish		Ramcharitar et al. (2006)	22
				PAL	Horodsky et al. (Horodysky et al. 2008)	21a, b
		*Leiostomus xanthurus*	Spot		Ramcharitar et al. (2006)	22
				PAL	Horodsky et al. (2008)	21a, b
		*Bairdiella chrysoura*	Silver perch		Ramcharitar et al. (2004)	22
		*Sciaena umbra*	Brown meagre	PAL	Wysocki et al. (2009a, b)	21a, b
				Boat noise masking, vocalization	Codarin et al. (2009)	
		*Cynoscion nebulosus*	Spotted seatrout	PAL	Horodsky et al. (2008)	21a, b
		*Micropogonias undulatus*	Atlantic croaker	PAL	Horodsky et al. (2008)	21a, b
				WN exposure, TTS	Ramcharitar and Popper (2004)	22
		*Sciaenops ocellatus*	Red drum	PAL	Horodsky et al. (2008)	21a, b
		*Menticirrhus saxatilis*	Northern kingfish	PAL	Horodsky et al. (2008)	21a, b
		*Pogonias chromis*	Black drum	WN exposure, TTS	Ramcharitar and Popper (2004)	22
					Loscacio and Mann (2011)	
	Cichlidae	*Astronotus ocellatus*	Oscar		Kenyon et al. (1998)	23
		*Neolamprologus brichardi*	Princess of Burundi		Ladich and Wysocki (2003)	23
		*Tramitichromis intermedius*			Ripley et al. (2002)	23
		*Oreochromis niloticus*	Nile tilapia	WN exposure, TTS	Smith et al. (2004b)	23
		*Astatotilapia burtoni*		Sex, dominance, reproductive status	Maruska et al. (2012)	73
		*Etroplus maculatus*	Orange chromide	PAL	Schulz-Mirbach et al. (2012)	24a, b
		*Paratilapia polleni*		PAL	Schulz-Mirbach et al. (2012)	24a, b
		*Hemichromis maculatus*	Jewel cichlid	PAL	Schulz-Mirbach et al. (2012)	24a, b
		*Steatocranus tinanti*	Slender lionhead cichlid	PAL	Schulz-Mirbach et al. (2012)	24a, b
	Pomacentridae	*Amphiprion frenatus*	Tomato clownfish	Vocalizations	Parmentier et al. (2009)	25a
		*Amphiprion ocellaris*	Clownfish anemonefish	Vocalizations	Parmentier et al. (2009)	25a
		*Amphiprion clarkii*	Yellowtail clownfish	Vocalizations	Parmentier et al. (2009)	25a
		*Chromis chromis*	Mediterranian damselfish	Boat noise masking, vocalization	Codarin et al. (2009)	
				PAL	Wysocki et al. (2009a)	25a, b
		*Abudefduf saxatilis*	Sergeant major damselfish	Ontogeny	Egner and Mann (2005)	25a
		*Abudefduf abdominalis*	Hawaiian sergeant damselfish	Sex, vocalizations	Maruska et al. (2007)	25a
		*Pomacentrus nagasakiensis*	Nagasaki damselfish	Settlement-stage larvae	Wright et al. (2010)	25a
		*Pomacentrus amboinsis*	Ambon damselfish	Settlement-stage larvae	Wright et al. (2010)	25a
	Gobiidae	*Gobius cruentatus*	Red-mouthed goby	PAL	Wysocki et al. (2009a)	25b, 26
		*Neogobius melanostomus*	Round goby	PAL, size	Belanger et al. (2010)	26
		*Padogobius bonelli*	Padanian goby	Vocalization, Ambient noise	Lugli et al. (2003)	26
		*Gobius nigricans*	Arno goby	Vocalization, Ambient noise	Lugli et al. (2003)	26
	Osphronemidae	*Trichopsis vittata*	Croaking gourami	Vocalization	Ladich and Yan (1998)	27
				Ontogeny	Wysocki and Ladich (2001)	
				Temporal resolution	Wysocki and Ladich (2002)	
				AEP to vocalization	Wysocki and Ladich (2003)	
		*Trichopsis pumila*	Pygmy gourami	Vocalization	Ladich and Yan (1998)	27
		*Trichogaster trichopterus*	Blue gourami		Ladich and Yan (1998)	27
				SBO deflation	Yan (1998)	27
				Temporal resolution	Wysocki and Ladich (2002)	
				Swimbladder deflation	Yan et al. (2000)	
		*Colisa lalia*	Dwarf gourami	Vocalization	Ladich and Yan (1998)	27
				SBO deflation	Yan (1998)	27
		*Macropodus opercularis*	Paradise fish		Ladich and Yan (1998)	27
	Helostomatidae	*Helostoma temmincki*	Kissing gourami	SBO deflation	Yan (1998)	27
Ceratodontiformes	Protopteridae	*Protopterus annectens*	African lungfish	PAL	Christensen-Daalsgard et al. (2011)	27a, b

Systematics according to Nelson (2006). Additional variable indicates that additional data are available besides baseline AEP—thresholds. Sound pressure level thresholds are given in dB re 1 μPa; all particle motion thresholds are given in dB re 1 μm/s^2^

*AEP* auditory evoked potentials, *ECG* electrocardiogram, *LFA* low frequency active sonar, *MFAS* midfrequency active sonar, *PAL* particle acceleration level, *SBO* suprabranchial organ, *TTS* temporary threshold shift, *WN* white noise. Common names are given according to www.fishbase.org

Families most often investigated in terms of species numbers are sciaenids or drums (11 species), cyprinids and cichlids (9 species), and pomacentrids or damselfish (8 species). The species most often chosen for investigations in AEP-studies is the goldfish (17 studies). Baseline audiograms were not the main purpose of many of these studies but were determined to investigate other issues such as the effects of accessory hearing structures, of noise exposure or masking, or of changes during ontogeny (see following sections and the column labelled “Additional Variable” in Table 1).

The particle acceleration (Fig. 8b) audiograms for elasmobranchs indicate a general low-frequency, low-pass characteristic. Most species have been studied at very low frequencies (below 100 Hz), but the audiograms for the nurse shark *Ginglyostoma cirratum* and the yellow stingray *Urobatis jamaicensis*, (Casper and Mann 2006) have not.

These particle acceleration audiograms are probably the best estimates of sensitivity because elasmobranchs lack a swim bladder or any other gas-filled structures that could give them sound pressure sensitivity. The various elasmobranch species differ in sensitivity by approximately 30 dB in the frequency range between 20 and 200 Hz, and are similar in particle acceleration sensitivity to all other species tested (Figs. 18b, 19b, 21b, 24b) except lungfish (Fig. 27b). There is no suggestion in these AEP audiograms of the remarkably great sensitivity suggested in behavioral studies of sound source localization (e.g., Myrberg et al. 1972).

The audiograms for the lake sturgeon *Acipenser fluvescens* (family Acipenseridae) and paddlefish *Polyodon spathula* (family Polyodontidae) (Fig. 9) (Lovell et al. 2005) are at least 30 dB less sensitive than the black baby whale *Brienomyrus brachyistius* (family Mormyridae). The former two species are likely sensitive to particle acceleration while the latter species is likely sound-pressure sensitive (Yan and Curtsinger 2000). For species that differ in the acoustic quantity to which they are most sensitive (sound pressure vs. particle acceleration), there is no rational way to compare thresholds when audiograms are expressed in sound pressure as in Fig. 9, except to say that particle acceleration species should appear less sensitive (by an unpredictable amount) and have a lower best frequency (<200 Hz) than pressure-sensitive species (500 Hz). The baby black whale exhibits a sensitivity and bandwidth generally similar to other species that are specialized for sound pressure detection.

Representatives of the family Clupeidae are specialized for sound pressure detection by virtue of an air-filled bulla adjacent to the utricle. Audiograms for these species in the region between 100 and 4,000 Hz (Fig. 10) have uncharacteristically high thresholds compared with other species that are specially adapted to detect sound pressure. The shapes of the low-frequency (<2,000 Hz) portions of these audiograms are typical of species specialized for detecting sound pressure, but were possibly masked by ambient noise.

Some clupeids (American shad *Alosa sapidissima* and gulf menhaden *Brevoortia patronus*; subfamily Alosinae) respond to ultrasound (in the range between 20 and about 90 kHz; Fig. 10), although sensitivity in this frequency range is poor compared with other species that are sound pressure sensitive. Alosinae have been shown to respond to frequencies of over 180 kHz (e.g., Mann et al. 1997). There is some evidence that the main receptor organs that may play a role in this sensitivity are the utricle and the cephalic lateral line organs in combination with the air-filled bullae (Wilson et al. 2009).

Representatives of all four otophysine orders (Cypriniformes, Siluriformes, Characiformes, Gymnotiformes) have been investigated. Among the order Cypriniformes the largest number of species has been investigated in the family Cyprinidae. Audiograms gained in representatives of the family Cyprinidae reveal considerable variation (Fig. 11). The hearing curves of common carps, the lake chub *Couesius plumbeus*, the topmouth minnow *Pseudorasbora parva* and the fathead minnow *Pimephales promelas* are similar to one another. Best thresholds for the zebrafish *Danio rerio*, the bighead carp *Aristichthys nobilis* and silver carp *Hypopthalmichthys molitrix* are 40–50 dB higher than thresholds of common carps. The difference of up to 50 dB between audiograms for these sound pressure sensitive species cannot be explained morphologically because all species possess swim bladders and Weberian ossicles. This is in contrast to catfishes which show a reduction in swim bladder size and number of Weberian ossicles (Bird and Hernandez 2007; Lechner and Ladich 2008). It is assumed that this difference is due to one of the technical factors described in “Behavioral and AEP thresholds in the goldfish *Carassius auratus*” section and discussed by Ladich and Wysocki (2009).

Representatives of three otophysan orders (Fig. 12) (longnose sucker *Catostomus catostomus*, family Catostomidae, order Cypriniformes; red piranha *Pygocentrus* (formerly *Serrasalmus*) *nattereri*, family Characidae, order Characiformes; glass knifefish *Eigenmannia virscens*, family Sternopygidae, order Gymnotiformes) are all specialized for sound pressure detection, and they all have similar audiograms in terms of sensitivity (maximum difference 15–20 dB) and frequency range of best hearing (0.6–1 kHz).

Siluriformes (catfishes—Figs. 13, 14, 15) are the most species-rich otophysan order (more than 3,300 species) possessing relatively great sensitivity to sound pressure (lowest thresholds below 90 dB) and a high frequency range of best hearing (500–1,000 Hz). Representatives of the families Callichthyidae and Loricariidae possess paired, tiny and encapsulated swim bladders and 1–2 Weberian ossicles which result in poorer sensitivities above 1 kHz as compared to the other catfish families. Representatives of all other families investigated—Doradidae, Auchenipteridae, Pseudopimelodidae, Heptapteridae, Mochokidae, Siluridae, Pimelodidae, Ariidae and Claroteidae—possess large unpaired swim bladders and typically 3–4 Weberian ossicles (Lechner and Ladich 2008). Best thresholds within the latter families differ by about 25 dB (Fig. 14) and 35 dB (Fig. 15), respectively.

Salmonids (Fig. 16) are primarily particle acceleration sensitive, as has been demonstrated behaviorally for the Atlantic salmon (Hawkins and Johnstone 1978). Therefore, these sound pressure audiograms are almost certainly inappropriate as estimates of their hearing. However, the relatively high sound pressure thresholds and the low-frequency range of best hearing (100–300 Hz) are to be expected from particle acceleration sensitive species.

Batrachoidiformes (toadfishes), Esociformes (pikes) and Percopsiformes (trout-perches) are sensitive to particle acceleration, not sound pressure (Fig. 17). This was demonstrated for the oyster toadfish by Yan et al. (2000; however, see caveat in “Using AEP-technique to investigate accessory hearing structures” section). Most of these species have a low or very low frequency of best hearing (<50–200 Hz). Gadiformes such as the Atlantic cod (family Gadidae), on the other hand, are sensitive to sound pressure at the higher frequencies (above 100 Hz—Chapman and Hawkins 1973), and the swim bladder has been shown to function in hearing (see also Sand and Enger 1973).

Figure 18 shows four species out of three orders namely Cyprinodontiformes (killifishes), Gasterosteiformes (sticklebacks) and Scorpaeniformes (mail-cheeked fishes) that have a low frequency of best hearing (200 Hz), suggesting that they are particle acceleration sensitive. Figure 18b shows great sensitivity to particle acceleration at 200 Hz in the Atlantic molly *Poecilia* *mexicana* (family Poeciliidae), but about 30 dB less sensitivity for the lined seahorse *Hippocampus erectus* (family Syngnathidae).

Among Perciformes (perches), the most species-rich fish order, representatives of 13 (out of 160) families have been investigated (Table 1). Representatives of the families Serranidae and Centrarchidae (Fig. 19) are probably particle acceleration sensitive as they all have a low or very low frequency of best hearing (<100–300 Hz) and relatively poor sound pressure sensitivity. The particle acceleration function (Fig. 19b) for the two basses correspond well with the sound pressure functions (Fig. 19a) in indicating a frequency of best hearing below 100 Hz.

The perciform audiograms depicted in Fig. 20 fall into two groups based on the sensitivity. The Spanish flag snapper *Lutjanus carponotatus* (family Lutjanidae), the rainbow runner *Elagatis bipinnulata*, and the golden trevally *Gnathonodon speciosus* (family Carangidae) (Wright et al. 2010) have low-pass audiogram shapes and poor sensitivity. The remaining three species have bandpass audiograms with a best frequency at 200 Hz, and seem to be more sensitive. We assume that the difference between the first group and the second group is mostly based on methodical differences.

Most of the representatives of the family Sciaenidae of Fig. 21a and b (Horodsky et al. 2008) were studied using the same techniques in the same lab, except for the brown meagre *Sciaena umbra* (Wysocki et al. 2009a). In general, they have similar audiograms, with a very low frequency of best hearing in terms of particle acceleration (<100–400 Hz—Fig. 21b). The relatively high sound pressure thresholds (above 90 dB in Fig. 21a) and the low frequency shape of the particle acceleration audiograms (Fig. 21b) suggest that they are not primarily sound-pressure sensitive. The brown meagre (Wysocki et al. 2009a) and the silver perch *Bardiella chrysoura* (Ramcharitar et al. 2004) may be exceptions, having relatively low sound pressure thresholds and a wide bandwidth of hearing, suggesting that they may respond primarily to sound pressure. The silver perch possess forward extentions of the swim bladder that increase the probability that they respond primarily to sound pressure but such extensions are lacking in the brown meagre. The sciaenid audiograms shown in Fig. 22 were determined in essentially the same lab using similar techniques. These species are likely to be more sensitive to particle acceleration because of the low-frequency hearing range and the relatively high sound pressure thresholds.

Representatives of the species-rich family Cichlidae (more than 1,300 species) of Figs. 23 and 24a and b represent a large diversity in hearing sensitivities due to the large differences in swim bladder anatomy. Some possess tiny reduced swim bladders (slender lionhead cichlid *Steatocranus tinanti*), some large swim bladders (jewel cichlid *Hemichromis guttatus*) and some anterior extensions of the swim bladder contacting the inner ear (orange chromide *Etroplus maculatus* and *Paratilapia polleni*) (Schulz-Mirbach et al. 2012). Thus some are likely particle acceleration sensitive due to the low-pass nature of the audiogram shapes (best frequency of hearing <100 Hz) and some sound pressure sensitive. The audiograms of the cichlid *Astatotilapia burtoni* are given in Fig. 73.

Comparison of the SPL and PAL audiograms for *Etroplus* and *Paratilapia* (Fig. 24a, b) reveals that the particle acceleration audiograms show good sensitivity out to much higher frequencies than is the case for some other cichlids (*Hemichromis* and *Steatocranus*). The sound pressure curves for *Etroplus* and *Paratilapia* are similarly quite sensitive at the higher frequencies. This comparison could be explained in at least two ways. The first possible explanation is that *Etroplus* and *Paratilapia* are both primarily sensitive to sound pressure and that plotting their audiograms in particle acceleration terms only reflects PALs in the tank at threshold. A second possible explanation is that these two species possess additional frequency-selective peripheral channels tuned at higher frequencies that are also activated by particle acceleration. Presently, we do not have enough experimental data to critically evaluate these two possibilities.

Members of the family Pomacentridae of Fig. 25a and b fall into two groups based on sound pressure thresholds, with the tomato clownfish *Amphiprion frenatus*, Mediterranean damselfish *Chromis chromis*, yellow clownfish *Amphiprion clarkii*, and clown anemone fish *Amphiprion ocellaris* much more sensitive below about 800 Hz. However, all these species have relatively high sound pressure thresholds (>90–100 dB) and a low-pass shaped audiogram, suggesting that they are primarily sensitive to particle acceleration. Figure 25b, however, indicates relatively high particle acceleration thresholds for the two species investigated.

The Gobiidae of Fig. 26 have relatively high sound pressure thresholds (>100 dB) but diverse audiogram shapes. The round goby *Neogobius melanostomus* is probably sensitive only to particle acceleration based on its high sound pressure thresholds, as is probably the Padanian goby *Padogobius bonelli* (formerly *martensi*) and the Arno goby *Gobius nigricans*, based on their low-frequency best hearing frequency (<100 Hz). Gobiids sometimes lack swim bladders.

The gouramis (families Osphronemidae and Helostomatidae) of Fig. 27 are probably all sensitive to sound pressure due to their suprabranchial air-breathing organ closely attached to the inner ear. Audiogram reveal wide bandwidth of hearing and fairly sensitive sound pressure thresholds. The blue gourami *Trichogaster trichopterus* seems unusual in having a band-pass shaped audiogram with a best frequency of 800 Hz, although it is similar to the others in having relatively good high-frequency hearing (>3,000 Hz) (See “Using AEP-technique to investigate accessory hearing structures” section).

The African lungfish *Protopterus annectens* (family Protopteridae) of Fig. 28a and b has been shown to be particle acceleration sensitive (Fig. 28b), although its particle acceleration thresholds seem unusually high, and its bandwidth of hearing is relatively wide (>2,000 Hz). The AEP method used here (Christensen-Dalsgaard et al. 2011) was unique in that the thresholds were obtained using a masking paradigm in which a broad band impulse, containing a wide range of frequencies was the signal and long duration pure tones of various frequencies served as maskers. This technique was used in order to obtain reliable thresholds at especially low frequencies, but the thresholds obtained this way were not compared at higher frequencies with thresholds obtained using the standard AEP method (e.g., Kenyon et al. 1998).

### General discussion of baseline audiograms

A comparison of hearing sensitivities in closely related species reveals a variety of trends. Hearing thresholds are either quite similar in some taxa such as catfishes (order Siluriformes, Figs. 13, 14 and 15) or in different species of drums (family Sciaenidae, Figs. 21a, b, 22), or audiograms resemble each other at certain frequencies e.g., in salmonids (family Salmonidae, Fig. 16). In some species or families audiograms deviate from each other considerably such as in toadfishes (family Batrachoididae, Fig. 17), cyprinids and damselfish (family Pomacentridae, Fig. 25a). In cyprinids differences in thresholds of up to 50 dB were found at particular frequencies (Fig. 11). Differences between species within a family could be due to real differences in sensitivity but, as it is suggested for cyprinids, toadfishes and gobiids, these could be due to different methods applied, including the acoustic characteristics of the test tanks. Reasons for this phenomenon have been discussed in goldfish by Hawkins (1981) for the diversity of behavioral audiograms and by Ladich and Wysocki (2009) and in “Comparison of different AEP-protocols”section for AEP audiograms.

Comparing audiograms from different taxa reveals general trends. Fishes lacking hearing spezializations such as elasmobranchs (sharks and rays), sturgeons, salmonids, pikes (Esocidae), gobiids, some cichlids, damselfish, sunfish (Centrarchidae) have best sound pressure hearing thresholds at 90 dB re 1 μPa or higher and maximal upper frequencies of 1–2 kHz (Figs. 8, 9, 16, 17, 18, 19, 23). For most species lacking hearing specializations and having particle acceleration threshold measurements, minimum thresholds are in the range between 30 and 40 dB re: 1 μm s^−2^, and best detection frequencies tend to be very low (<100 Hz). In contrast, species possessing hearing specializations such as weakly electric fish (family Mormyridae), otophysines and gouramis (Figs. 9, 11, 12, 13, 14, 15, 27) have best hearing thresholds (sound pressure) below 90 dB and upper frequency limits of 3–6 kHz (Ladich 1999; Yan and Curtsinger 2000; Lechner and Ladich 2008 etc.). In additional species such as the silver perch (family Sciaenidae, Fig. 22), the cichlids (*Etroplus* and *Paratilapia*, Figs. 23 and 24a), and the silver mojarra *Eucinostomus argenteus* (family Gerreidae, Fig. 20) a close connection between the swim bladder and the inner ear has been found and their sound pressure thresholds are accordingly low (Ramcharitar et al. 2004; Parmentier et al. 2011; Schulz-Mirbach et al. 2012). It must also be noted that the relationship between morphological connections and hearing sensitivities are not always straightforward. In clupeids swim bladders are connected to the inner ear via an anterior extension but hearing sensitivities are relatively poor (Fig. 10). None of the clupeid species exhibits auditory thresholds below 100 dB (Mann et al. 2001). The hearing specialization obviously evolved for detecting ultrasound in some species (subfamily Alosinae) (Fig. 10), but is not particularly effective for detecting low levels at the lower frequencies (American shad, Gulf menhaden, subfamily Alosinae). Among sciaenids bearing swim bladders, those possessing diverticulae (weakfish *Cynoscion regalis*, spotted seatrout *Cynoscion nebulosus* and Atlantic croaker *Micropogonias undulatus*) had generally lower pressure thresholds than species lacking diverticulae (spot and red drum). However, the lowest sound pressure thresholds at higher frequencies (800–1,100 Hz) occurred in northern kingfish *Menticirrhus saxatilis*, a species with low hair cell densities and swim bladder that atrophies as adults (Ramcharitar et al. 2001). These lower sound pressure thresholds of kingfish may be due to a combination of particle acceleration and sound pressure sensitivity (Horodysky et al. 2008).

## Using AEP-technique to investigate accessory hearing structures

Many fish taxa possess accessory hearing structures which are thought to enhance their hearing sensitivities by extending the detectable frequency range and by lowering their hearing thresholds. This is made possible by connecting the inner ear to air-filled cavities and transmitting the oscillations of the wall of these cavities in a field of fluctuating sound pressure to the inner ears. Accessory hearing structures have developed in numerous species. Some taxa such as some holocentrids and clupeids develop anterior swim bladder horns, otophysines possess Weberian ossicles, whereas gouramis and mormyrids possess air-filled bubbles touching the inner ear without being connected to the swim bladder (for reviews see Ladich and Popper 2004; Braun and Grande 2008). Experiments that include removal of the gas cavity are one of the ways to help determine whether or not the species studied is primarily pressure sensitive or particle acceleration sensitive. If removal of the gas bladder results in a reduction of sound pressure sensitivity, then the species studied is probably primarily sound-pressure sensitive; if not, then the species is primarily particle acceleration sensitive, at least in the acoustic test tank environment employed. After a swim bladder is deflated, the fish becomes, in effect, only sensitive to particle motion which was generally not measured in the studies reviewed here. The differences in pre- and post-deflation audiograms possibly reflect differences in PAL in the tanks used, and/or differences in particle motion sensitivity of the species studied. Before the introduction of the AEP-technique several authors investigated the functional significance using behavioral or microphonic techniques to study this question (Schneider 1941; Poggendorf 1952; Kleerekoper and Roggenkamp 1959; Fay and Popper 1974, 1975).

The AEP-techniques proved to be a useful tool to measure the status of auditory sensitivity after eliminating the accessory hearing structures. The AEP-technique is typically a non-invasive approach and thus animals can be measured repeatedly before and after the elimination of various morphological structures.

**Fig. 29:**
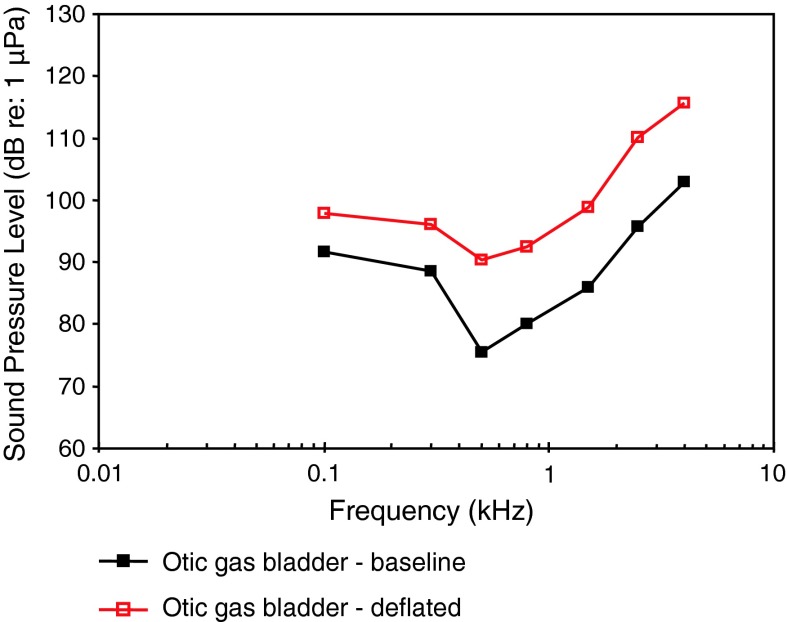
Hearing sensitivity in the black baby whale *Brienomyrus brachyistius* before and after removal of the gas from the otic gas bladder. After Yan and Curtsinger (2000)

Removal of the gas from the otic gas bladder in black baby whale, a weakly electric mormyrid, resulted in a nearly parallel decline in sensitivity (about 6 dB to a maximum of 15 dB at 500 Hz) from 100 Hz to 4 kHz (Yan and Curtsinger 2000) (Fig. 29).

Clupeids possess anterior bulla-like extensions of the swim bladder which are in contact with the utricle and lateral line canal on the head. Wilson et al. (2009) investigated in what way this accessory hearing structure contributes to sensitivity to ultrasound in the Gulf menhaden. Filling the gas-filled bullae with Ringer solution reduced the response of the auditory system to 40 kHz tone bursts measured by the AEP technique indicating that the air-filled bullae are necessary for ultrasonic hearing.

**Fig. 30:**
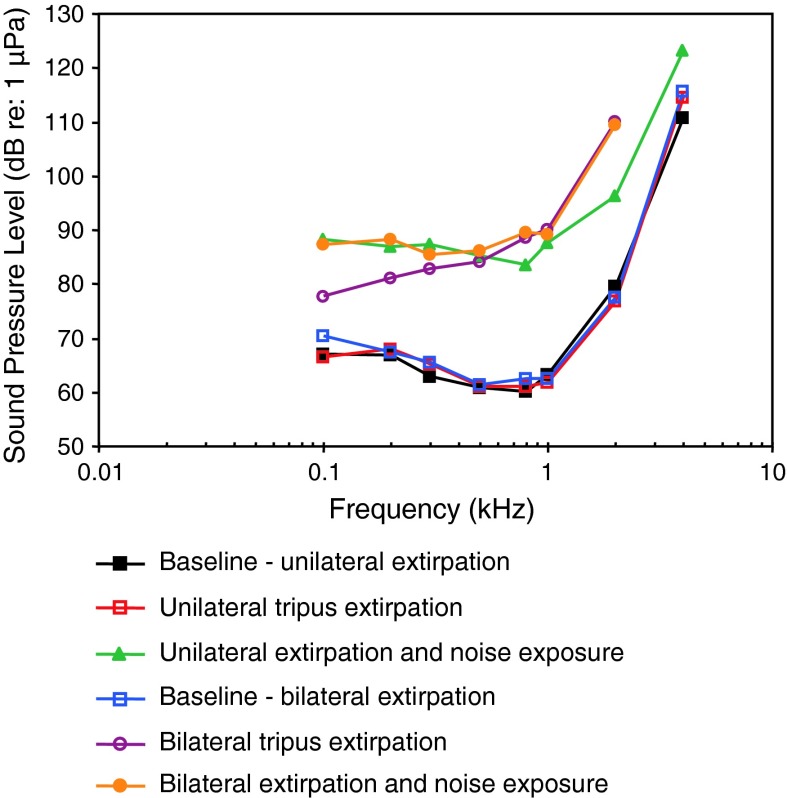
Hearing sensitivity in the goldfish before and after unilateral and bilateral extirpation of the tripodes. In addition, the effects of noise exposure in unilaterally and bilaterally extirpated animals are shown. After Ladich and Wysocki (2003)

The function of the swim bladder and the Weberian apparatus has been investigated in the goldfish using different experimental approaches. Ladich and Wysocki (2003) removed the tripodes, the most caudal of the Weberian ossicles, and observed a highly frequency-dependent increase in thresholds from 7 dB at 100 Hz up to 35 dB at 2 kHz (Fig. 30).

**Fig. 31:**
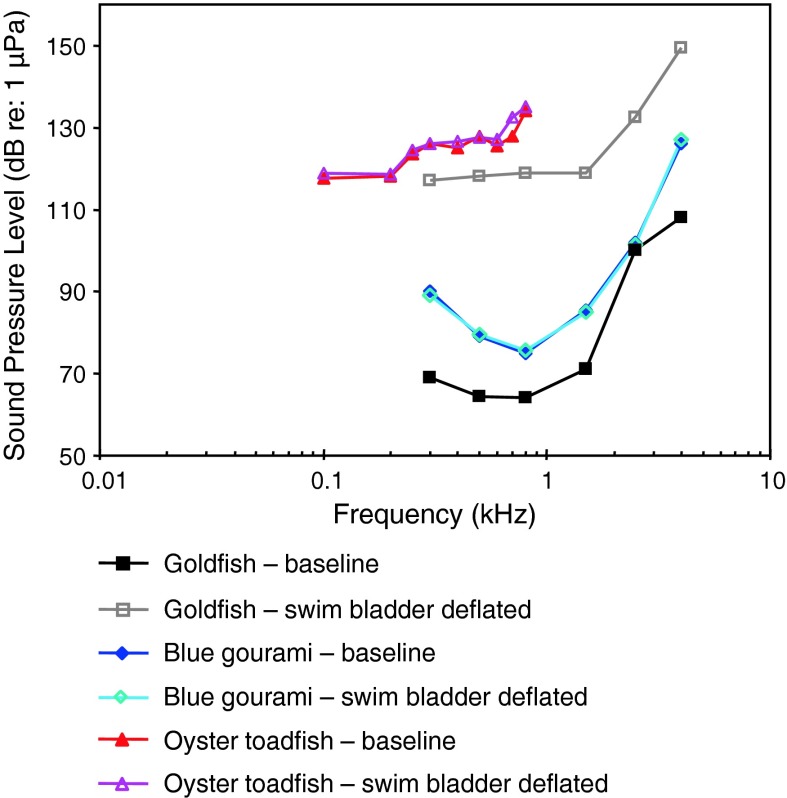
Effects of swim bladder deflation in the goldfish, the oyster toadfish *Opsanus tau* and the blue gourami *Trichogaster trichopterus*. Swim bladder deflation affected hearing sensitivity only in the goldfish. After Yan et al. (2000)

Yan et al. (2000) investigated the effect of swim bladder deflation in the goldfish and observed a rise in thresholds of approximately 50 dB between 300 Hz to 1.5 kHz and a somewhat smaller drop at 2.5 and 4 kHz (Fig. 31). Interestingly, elimination of the Weberian ossicles and the swim bladder had different effects in both goldfish studies (Figs. 30, 31). Swim bladder deflation affected hearing thresholds in the goldfish but not in the non-related oyster toadfish or the blue gourami (Yan et al. 2000). The latter two results indicate that the swim bladder is not connected acoustically to the inner ear in toadfishes and gouramis. One caveat regarding gas bladder deflation experiments is that negative findings (no effect of deflation) may not indicate the true effectiveness of the bladder for hearing in the species’ normal habitat. In deeper water (deeper than in the AEP measurement situation), where the ratio between sound pressure to particle velocity is expected to be considerably higher (higher impedance) the contribution of the swim bladder to hearing will appear greater than at the surface (Poggendorf 1952).

**Fig. 32:**
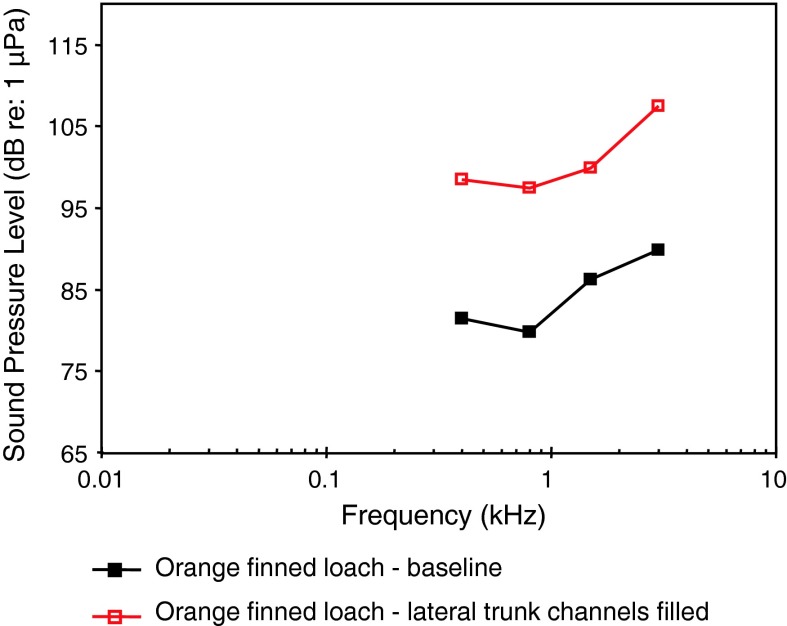
Auditory thresholds of the red finned loach *Yasuhikotakia modesta* before (baseline) and after filling the lateral trunk channels. After Kratochvil and Ladich (2000)

In representatives of the family Cobitidae (loaches), an otophysine family closely related to cyprinids, a second accessory hearing structure evolved in addition to Weberian ossicles. Loaches have a cranial encapsulation of the anterior part of the swim bladder and in addition special channels stretching laterally from the swim bladder to the outer body wall. These lateral trunk channels form a muscle-free window beneath the skin. Filling the lateral trunk channels with cotton/rayon stapple in the red finned loach *Yasuhikotakia modesta* resulted in an increase in thresholds of 14–18 dB indicating mechanical damping of the swim bladder (Fig. 32) (Kratochvil and Ladich 2000). These experiments indicate that lateral trunk channels enhance hearing sensitivity of cobitid fishes.

**Fig. 33:**
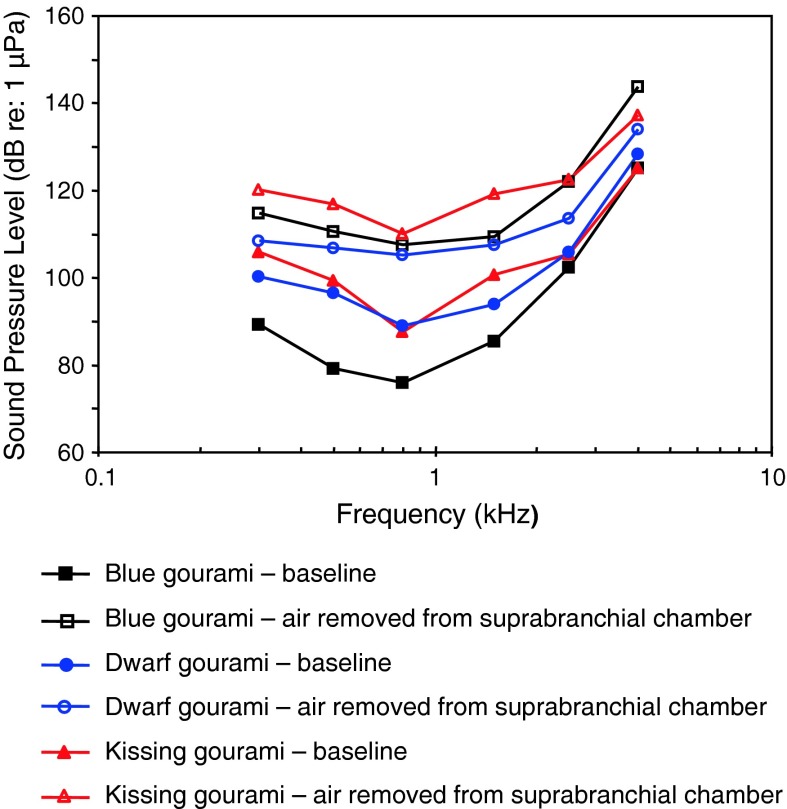
Effects of the removal of gas from the suprabranchial air-breathing organs by filling it with water in three gourami species, the blue gourami *Trichogaster trichopterus*, the dwarf gourami *Colisa lalia* and the kissing gourami *Helostoma temmincki*. After Yan (1998)

Gouramis or labyrinth fishes (suborder Anabantoidei) possess suprabranchial air-breathing chambers which enhance their hearing sensitivity as demonstrated by Schneider (1941) by conditioning the fish. Filling the chambers with water in three more gourami species (blue gourami; kissing gourami *Helostoma temminckii*; dwarf gourami *Colisa lalia*) resulted in an increase in thresholds between 5 and 25 dB as shown by Yan (1998) using the AEP technique (Fig. 33). The decrease was lowest at the highest frequencies (4 kHz). These results corroborate the hearing function of these air-breathing organs.

## Applying AEP-techniques to study ontogenetic development of hearing

Due to the difficulty training or conditioning juvenile fish of a few millimeter length, just one study investigated the ontogenetic development of hearing sensitivity using behavioral techniques (Kenyon 1996). Kenyon (1996) measured the hearing sensitivity in the bicolor damselfish *Stegastes* (formerly *Eupomacentrus*) *partitus*. Since the introduction of the AEP protocol in 1998 the ontogenetic development of hearing sensitivity has been investigated in eight species using the AEP technique, one representative of the family Clupeidae, one of the family Cyprinidae, two catfish species, one toadfish species and two perciforms. In general, hearing thresholds were given in sound pressure units but it is assumed that all fish species including those possessing hearing specialization are also particle acceleration sensitive.

**Fig. 34:**
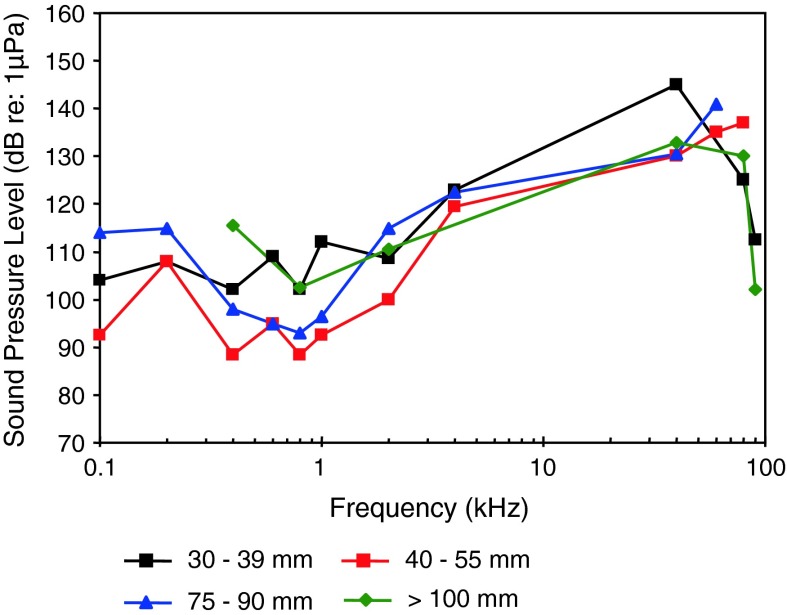
Development of auditory sensitivity in the American shad *Alosa sapidissima*. After Higgs et al. (2004)

Following the systematics by Nelson (2006) the family Clupeidae will be discussed first. Higgs et al. (2004) investigated the development in the American shad in order to find out when ultrasound detection begins. AEP thresholds in 4 different stages starting from 30 to 39 mm from 0.1 to 90 kHz were measured (Fig. 34). The sensitivity to sounds including ultrasound did not change developmentally for these size ranges.

**Fig. 35:**
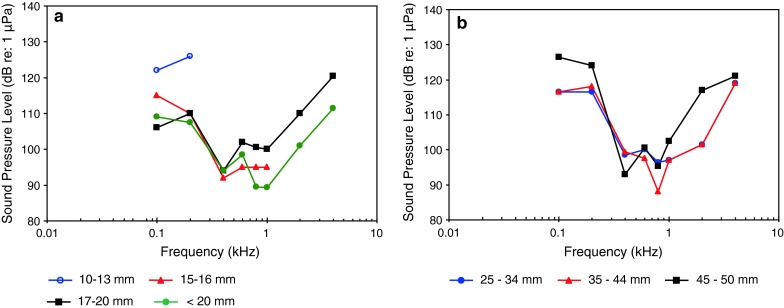
Development of auditory sensitivity in the zebrafish *Danio rerio* from **a** 10–20 mm and **b** from 25–50 mm. After Higgs et al. (2001, 2003)

Among otophysines the development in the zebrafish, a representative of the order Cypriniformes, and two catfish species have been studied. In the zebrafish, Higgs et al. (2001, 2003) observed an expansion of maximum detectable frequency from 200 Hz at 10 mm to 4,000 Hz at 45 mm total length (TL) but no change in auditory threshold, bandwidth, or best frequency over the size range of 34–50 mm TL (Fig. 35a, b).

**Fig. 36:**
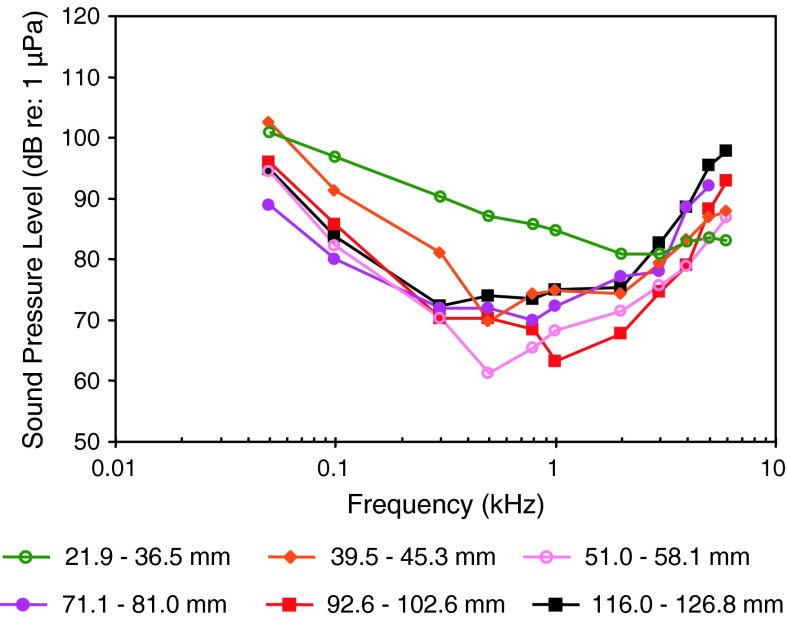
Development of auditory sensitivity in the squeaker catfish *Synodontis schoutedeni*. After Lechner et al. (2010)

**Fig. 37:**
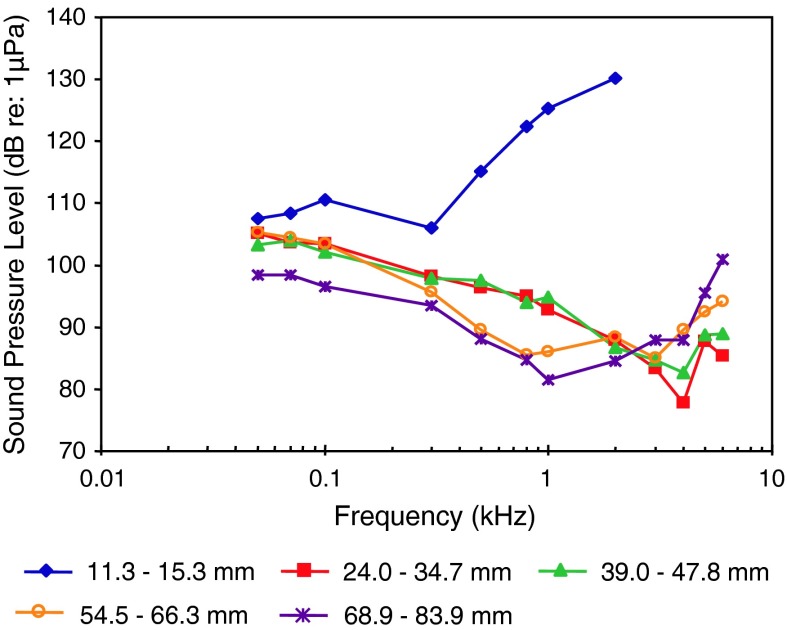
Development of auditory sensitivity in the African bullhead catfish *Lophiobagrus cyclurus*. After Lechner et al. (2011)

In contrast to the zebrafish, a change in sensitivity was observed in both catfish species studied. In the squeaker catfish *Synodontis schoutedeni* (family Mochokidae) larger stages showed significantly lower thresholds at frequencies below 2 kHz (Lechner et al. 2010) (Fig. 36). Similarly, in the African bullhead catfish *Lophiobagrus cyclurus* (family Bagridae), the smallest juveniles had the lowest auditory thresholds. They were unable to detect frequencies higher than 2 or 3 kHz (Fig. 37). In the latter the increase in sensitivity and detectable frequency range was attributed to the development of interossicular ligaments between Weberian ossicles (Lechner et al. 2011).

**Fig. 38:**
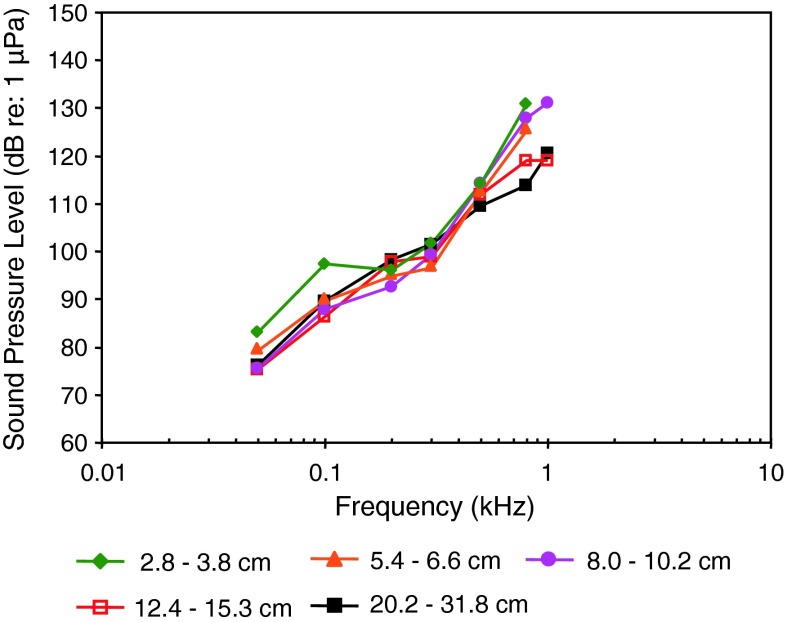
Development of auditory sensitivity in Lusitanian toadfish *Halobatrachus didactylus*. After Vasconcelos and Ladich (2008)

Among toadfishes the development of auditory sensitivity was studied in the Lusitanian toadfish (Vasconcelos and Ladich 2008). The best hearing was below 300 Hz in all age/size groups. Statistically significant higher hearing thresholds were found in the smallest juveniles at 100 Hz as well as at higher frequencies (800 and 1,000 Hz). A small increase in the detectable frequency range was observed with size increase (Fig. 38).

**Fig. 39:**
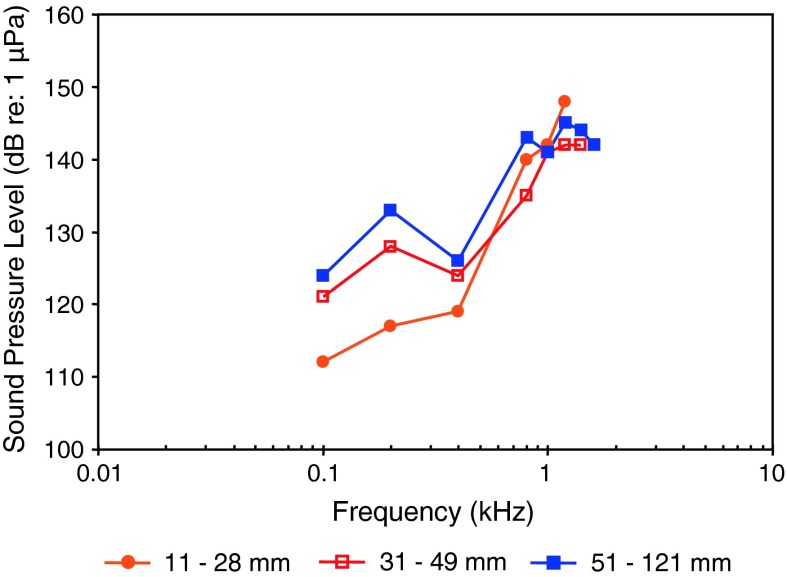
Development of auditory sensitivity in sergeant major damselfish *Abudefduf saxatilis*. After Egner and Mann (2005)

Within perciforms the ontogenetic development was measured in three species from three different families. Egner and Mann (2005) observed a change in sensitivity in the sergeant major damselfish *Abudefduf saxatilis* (family Pomacentridae) (Fig. 39) at 100 and 200 Hz. Younger stages were more sensitive to sounds. The larger fish (>50 mm) were more likely to respond to higher frequencies (1,000–1,600 Hz).

**Fig. 40:**
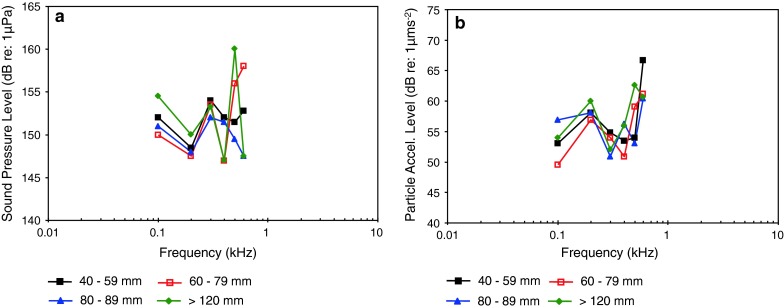
Development of auditory sensitivity in round goby *Neogobius melanostomus*. **a** SPL audiograms and **b** PAL audiograms. After Belanger et al. (2010)

In the round goby (family Gobiidae) Belanger et al. (2010) studied the hearing sensitivity in different sized stages from 40 to 120 mm. No effect of size on hearing was observed. This lack of a change in sensitivity may partly be due to the fact that earlier stages have not been measured (Fig. 40). The sound pressure (Fig. 40a) and particle acceleration thresholds (Fig. 40b) are both unusually high in this study, but it is likely that this species is primarily sensitive to particle acceleration.

**Fig. 41:**
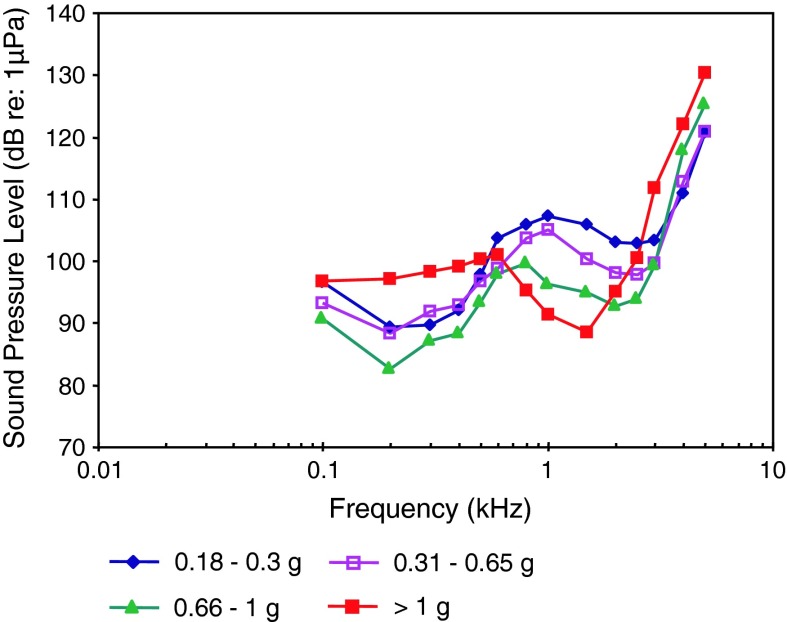
Development of auditory sensitivity in croaking gourami *Trichopsis vittata*. After Wysocki and Ladich (2001)

Within the third perciform family, the osphronemids, hearing development was investigated in the croaking gourami *Trichopsis vittata* (Wysocki and Ladich 2001). Absolute sensitivity increased whereas the most sensitive frequency decreased with growth (Fig. 41). The changes in sensitivity were attributed to the developmental changes in the air-breathing suprabranchial chamber, which functions as an accessory hearing organ.

## Applying AEP-techniques to study the effects of noise exposure

The effects of underwater noise on fish hearing have been investigated in two different ways. The first involved exposing fish to noise of high levels for different durations and studying the increase in auditory thresholds [temporary threshold shift (TTS)] afterwards. In addition, the time until fish recovered normal their hearing abilities was investigated. The second approach was to study the hearing sensitivity in the presence of noise (not after a certain period of noise exposure), a phenomenon known as masking. Masking effects are observable at much lower noise levels than effects of noise exposure, and occur in all vertebrates investigated (Fay 1988).

Noise exposure can result in a temporary increase in hearing thresholds (TTS) if the noise exposure levels are high enough; this varies considerably among species. The AEP-technique has proven to be very useful for these investigations because fish could be measured repeatedly to study the degree and recovery from any TTS over short recovery periods. Different noise types have been used including white noise, ambient noise, sonar, seismic shots (the sounds from air guns used in seismic exploration) and pure tones.

### Exposure to white noise

The majority of studies on white noise exposure were carried out in otophysines, in particular in cyprinids. The goldfish was the species most often used.

**Fig. 42:**
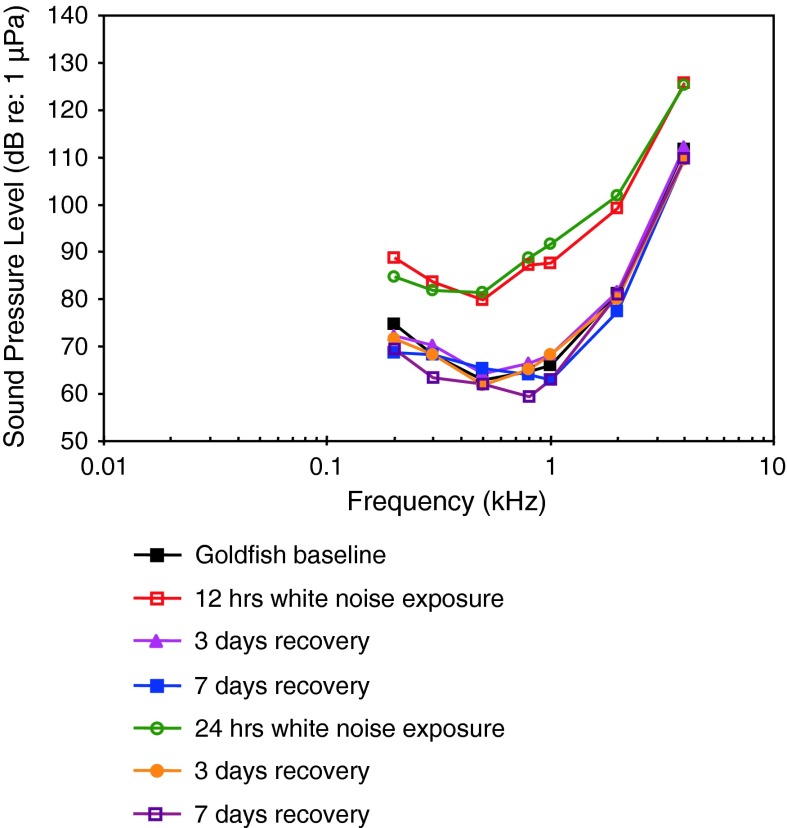
Auditory sensitivity of the goldfish before (baseline) and after exposure to white noise for 12 or 24 h at 158 dB. Thresholds are shown after 3 and 7 days of recovery. After Amoser and Ladich (2003)

Amoser and Ladich (2003) studied TTS and recovery in the goldfish after exposure for 12 or 24 h of 158 dB. The goldfish showed a significant rise (up to 26 dB) in thresholds immediately after exposure. Exposure duration had no effect on thresholds in this study and recovery took 3 days (Fig. 42).

**Fig. 43:**
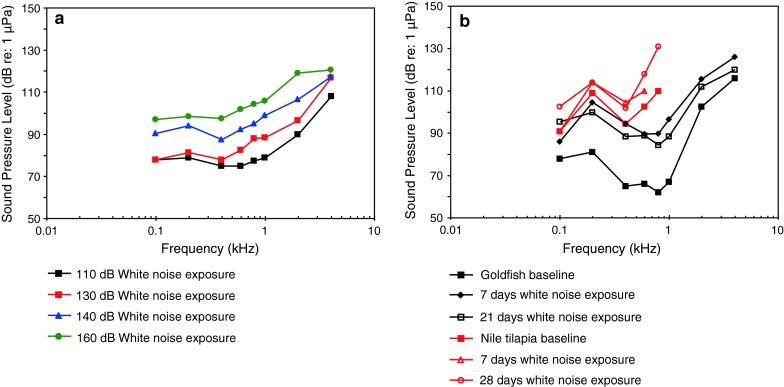
Auditory sensitivity of the goldfish after exposure to white noise of **a** different levels and **b** different time periods. Effects of exposure to different time periods are also shown for the Nile tilapia *Oreochromis niloticus*. After Smith et al. (2004a)

**Fig. 44:**
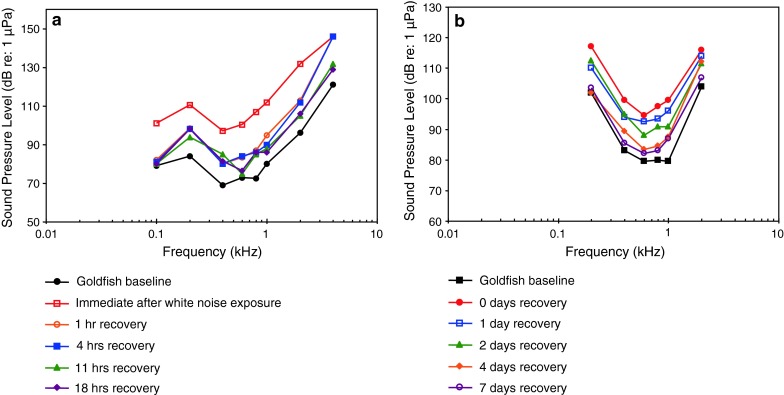
Auditory sensitivity of the goldfish after exposure to white noise and after different periods of recovery. **a** Recovery for 0–18 h and **b** recovery for 0–7 days. After Smith et al. (2004b, 2006)

Smith et al. (2004a) exposed goldfish to different levels of white noise (between 110 and 160 dB) for 24 h and found a correlation between time of exposure and TTS (Fig. 43a). Extending the exposure for 7 or 28 days resulted in no additional TTS in the goldfish or in the Nile tilapia *Oreochromis niloticus* (family Cichlidae) (Fig. 43b). In a subsequent study Smith et al. (2004b) found out that even 10 min of noise exposure (160–170 dB) resulted in a TTS of 5 dB (Fig. 44a). After 14 days of recovery thresholds were similar to baseline thresholds (Smith et al. 2004b) (Fig. 44a). In a subsequent study Smith et al. (2006) exposed goldfish to white noise of 170 dB for 48 h and found a small 4 dB TTS after 7 days of recovery (Fig. 44b).

**Fig. 45:**
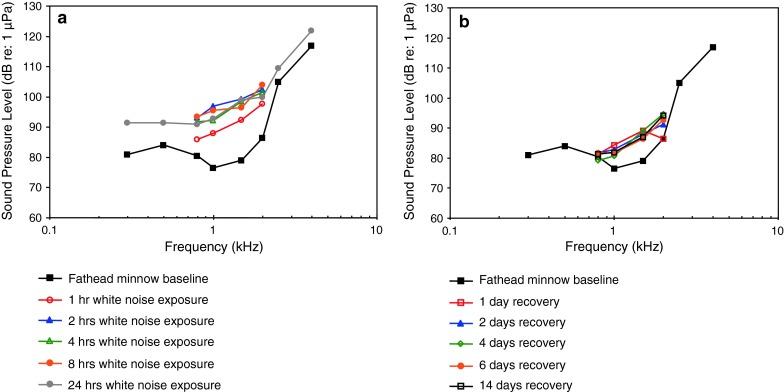
Auditory sensitivity of the fathead minnow *Pimephales promelas* (**a**) before (baseline) and after exposure to white noise at 142 dB for 1–24 h. **b** gives thresholds after exposure to 24 h and recovery for certain time periods. After Scholik and Yan (2001)

Scholik and Yan (2001) exposed fathead minnows (family Cyprinidae) to white noise between 0.3 and 4 kHz at 142 dB re 1 μPa for 1 to 24 h (Fig. 45a). Immediately after exposure, fish showed significantly higher thresholds compared to the control baseline fish at most test frequencies. The exposure effects appear to reach asymptote with a 2 h exposure. Recovery did not occur for at least 14 days (Fig. 45b).

**Fig. 46:**
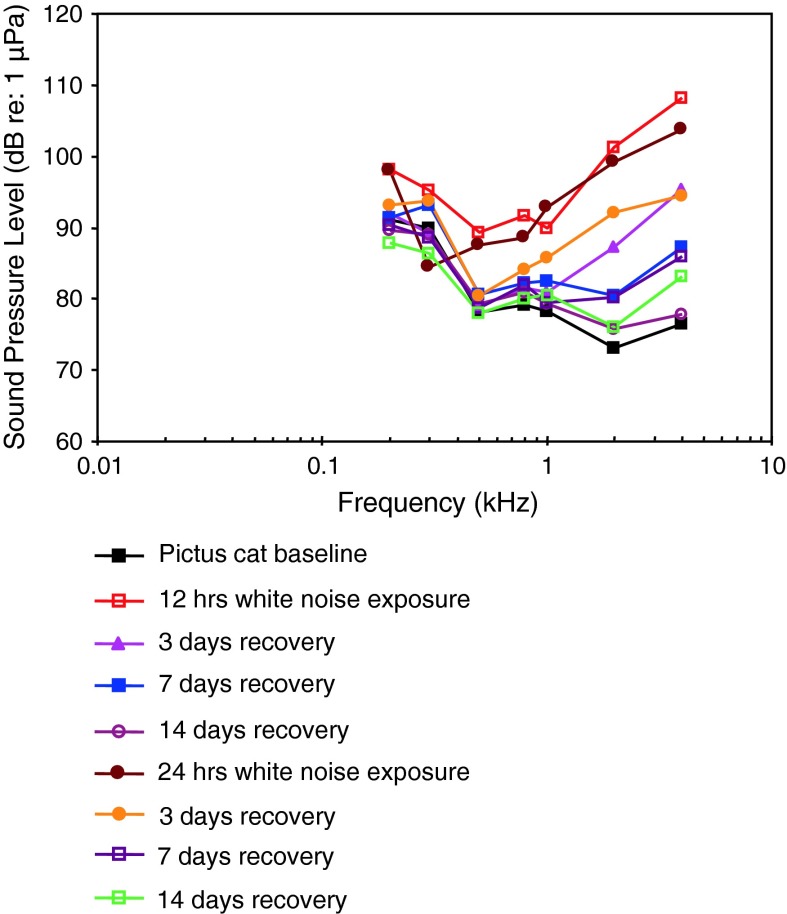
Auditory sensitivity of the pictus cat *Pimelodus pictus* before (baseline) and after exposure to white noise at 158 dB for 12 or 24 h. Thresholds are shown after 3 and 7 days of recovery. After Amoser and Ladich (2003)

Amoser and Ladich (2003) studied the TTS and recovery in one representative of catfishes, the Amazonian pimelodid pictus cat *Pimelodus pictus* (family Pimelodidae), after exposure for 12 or 24 h at 158 dB. The catfish showed a significant rise of thresholds of up to 32 dB after exposure. Exposure duration had no effect on sensitivity. Recovery took 14 days in the pictus cat (Fig. 46).

**Fig. 47:**
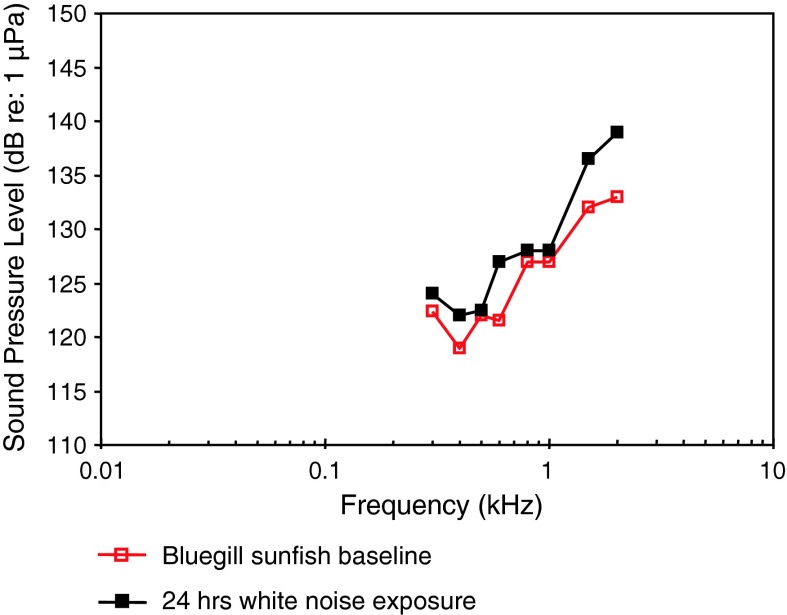
Auditory sensitivity of the bluegill sunfish *Lepomis macrochirus* before (baseline) and after exposure to white noise (142 dB) for 24 h. After Scholik and Yan (2002a)

Scholik and Yan (2002a) exposed the bluegill sunfish *Lepomis macrochirus* (family Centrarchidae) to white noise at 142 dB for 24 h (Fig. 47). The noise exposure had only a minimal effect (from about 1 to 6 dB TTS). It is likely that pre-exposure thresholds determine the magnitude of the effect of noise exposure, with the most sensitive species showing a greater effect of noise exposure (e.g., Smith et al. 2004b). The bluegill (Fig. 47) is not very sensitive to sound.

### Exposure to pure tones

**Fig. 48:**
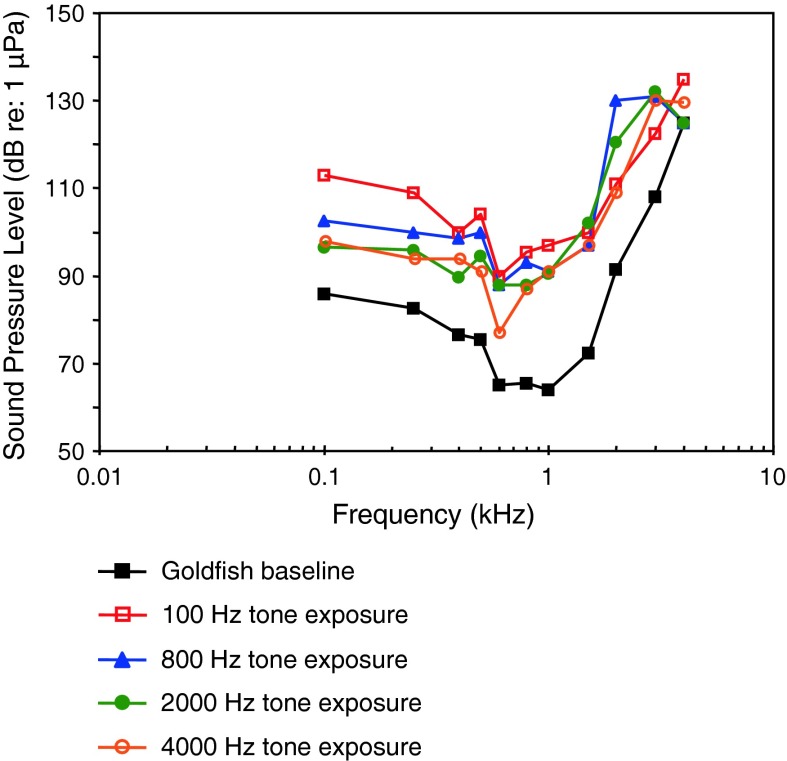
Auditory sensitivity of the goldfish before (baseline) and after exposure to pure tones at 178 dB for 48 h. After Smith et al. (2011)

Exposure to intense pure tones was carried out to find out if different frequencies cause damage in different frequency regions and possibly to different regions of the sensory epithelium. Smith et al. (2011) exposed goldfish to pure tones of 100, 800, 2,000 and 4,000 Hz for 48 h and found a frequency dependent TTS (Fig. 48). Smith et al. (2011) interpreted these findings as demonstrating a crude “place” representation of frequency on the saccular epithelium.

### Exposure to anthropogenic noise

Exposure to anthropogenic noise of different origin again resulted in TTS depending on noise source and time of exposure indicating the negative effects of human-made noise on hearing thresholds in fishes. The effects of anthropogenic noise on fishes has recently become a significant topic in the field of hearing and other effects (e.g., Popper and Hawkins 2012). The literature on noise effects on fish hearing is relatively recent, and all of it has made use of AEP methods (see “Applying AEP-techniques to study the effects of masking in fish” section below for anthropogenic noise masking experiments).

**Fig. 49:**
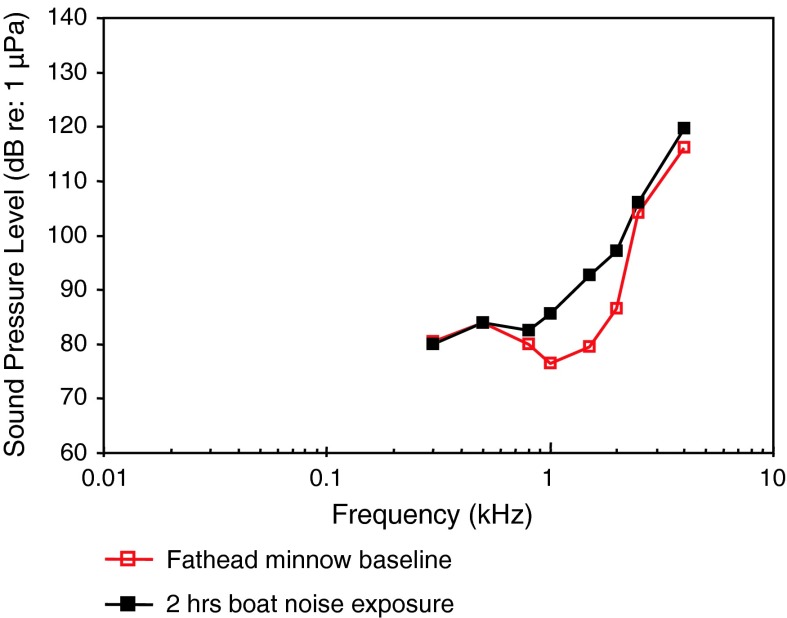
Auditory sensitivity of the fathead minnow *Pimephales. promelas* before and after exposure to boat noise for 2 h at 142 dB. After Scholik and Yan (2002b)

Scholik and Yan (2002b) measured the effect of exposure to 2 h of recorded boat noise at 142 dB in the fathead minnow. TTS was primarily observed at frequencies at which main energies of engine noise was concentrated (Fig. 49).

**Fig. 50:**
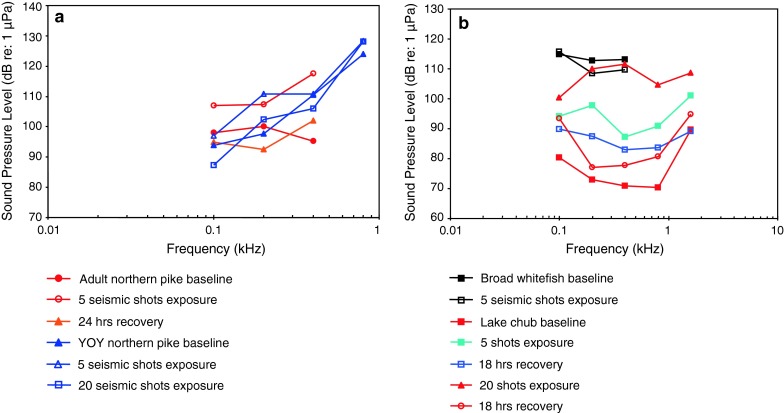
Auditory sensitivity of **a** the northern pike *Esox lucius* and **b** the broad whitefish *Coregonus nasus* and the lake chub *Couesius plumbeus* before and after exposure to seismic gun shots (5, 2) and different periods of recovery (18, 24 h) (average of 210 dB at the fish’s location). *YOY* Young of the year. After Popper et al. (2005)

Popper et al. (2005) investigated the effects of seismic shots from air guns (used in geophysical exploration) on the northern pike *Esox lucius* (Family Esocidae), the broad whitefish *Coregonus nasus* (family Coregonidae) and the lake chub *Couesius plumbeus* (family Cyprinidae) in the Mackenzie River Delta. Threshold shifts were found for exposed fish as compared to controls in the northern pike and lake chub, with recovery within 18 or 24 h of exposure, while there was no threshold shift in the broad whitefish (Fig. 50a, b).

**Fig. 51:**
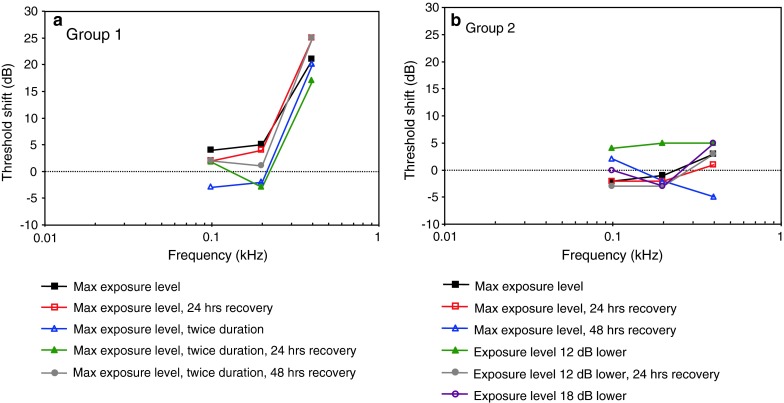
Threshold shifts of the rainbow trout *Oncorhynchus mykiss* when exposed to LFA sonar (193 dB) for different periods (324 or 648 s), different levels of attenuation (0, 12 or 18 dB) and after different periods of recovery (24 or 48 h). Note the differences between group 1 and group 2 (Figs. 51a, b). After Popper et al. (2007). *LFA* low-frequency active

Popper et al. (2007) measured the effects of low-frequency active sonar (LFA) on the hearing thresholds of rainbow trout *Oncorhynchus mykiss* (Family Salmonidae). Animals were exposed to a maximum received SPL of 193 dB re 1 Pa^2^ for 324 or 648 s. The TTS was more pronounced in one group of rainbow trout studied (group 1) than in another group (group 2) at 400 Hz (Figs. 51a, b).

**Fig. 52:**
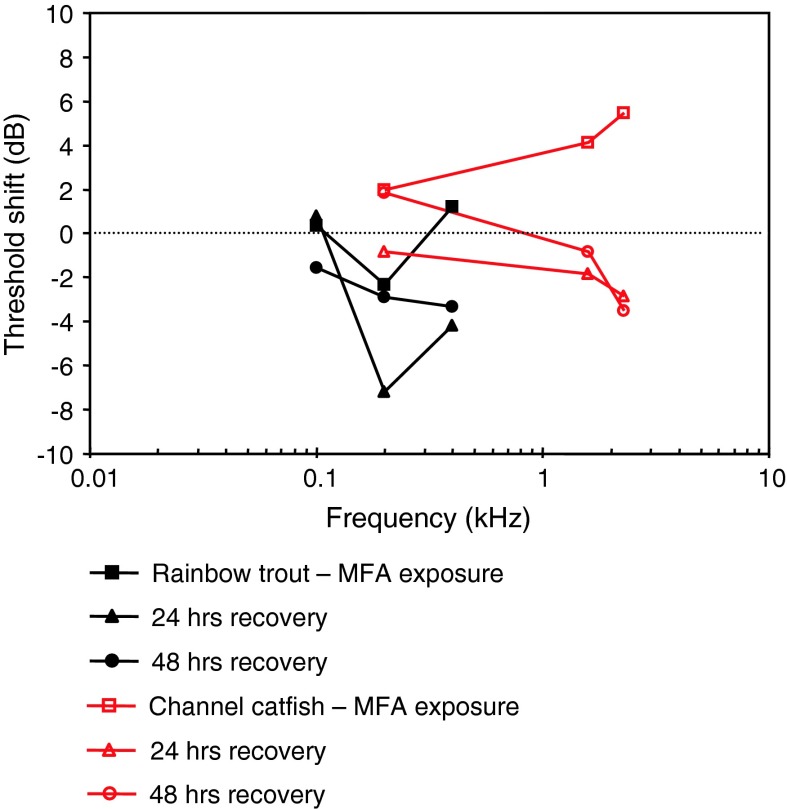
Threshold shifts of the rainbow trout *Oncorhynchus mykiss* and the channel catfish *Ictalurus punctatus* immediately after being exposed to MFA sonar at 210 dB and after 24 or 48 h of recovery. The average of two groups is shown. After Halvorsen et al. (2012). *MFA* mid-frequency active sonar

Halvorsen et al. (2012) studied the effects of exposure to mid-frequency active sonar (MFA—a military sonar) in rainbow trout and the channel catfish *Ictalurus punctatus* (family Ictaluridae) at SPLs of 210 dB between 2.8 and 3.6 kHz. The exposure level did not affect the hearing sensitivity of rainbow trout, a species whose hearing range is lower than the MFA frequencies, and is sensitive to particle acceleration. In contrast, one cohort of channel catfish (an otophysine) showed a TTS of 4–6 dB at 2.3 kHz, but not at lower tested frequencies, whereas a second cohort showed no change. The average of the two cohorts is plotted in Fig. 52.

**Fig. 53:**
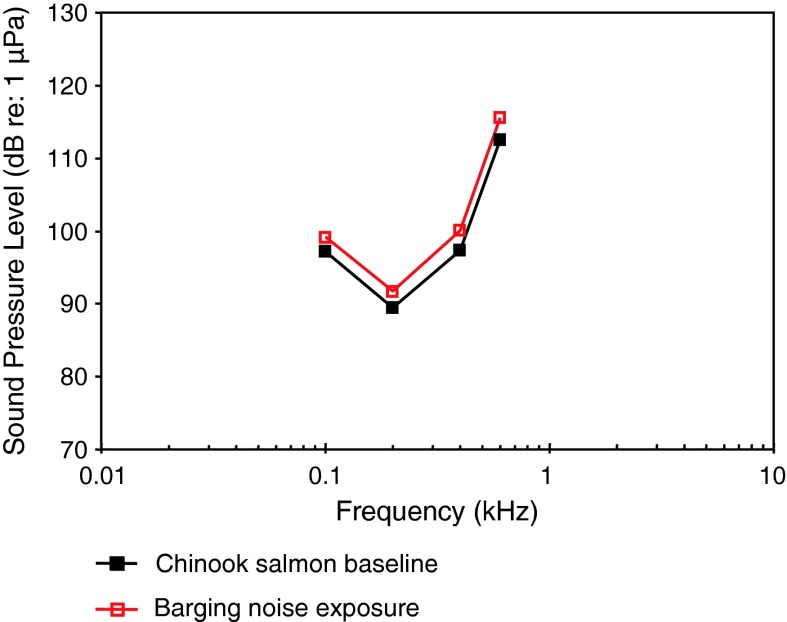
Auditory sensitivity of juvenile Chinook salmon *Oncorhynchus tshawytscha* before (baseline) and 7 days after exposure to barging noise (136 dB noise). After Halvorsen et al. (2009)

To avoid mortality caused by passage through dam turbines and spillways, juvenile Chinook salmon *Oncorhynchus tshawytscha* are annually transported downstream by barges in the Western USA through the federal hydropower system on the Snake and Columbia rivers. Barging noise of about 136 dB resulted in a small but significant TTS in juveniles 7 days after barging (Halvorsen et al. 2009) (Fig. 53).

## Applying AEP-techniques to study the effects of masking in fish

The hearing sensitivity in fish can be impaired in the presence of detectable noise, which raises the threshold above quiet conditions (masking). In contrast to the impairments mentioned in the previous section, the hearing thresholds are not measured after but during the occurrence of typically much lower noise levels and spectra. Different noise types have been applied to study masking effects, in particular white noise, natural ambient noise and anthropogenic noise. An increase in the background noise caused by natural or human activity sources may render the weakest sources undetectable, and may decrease the distance at which all sources can be detected.

### White noise masking

**Fig. 54:**
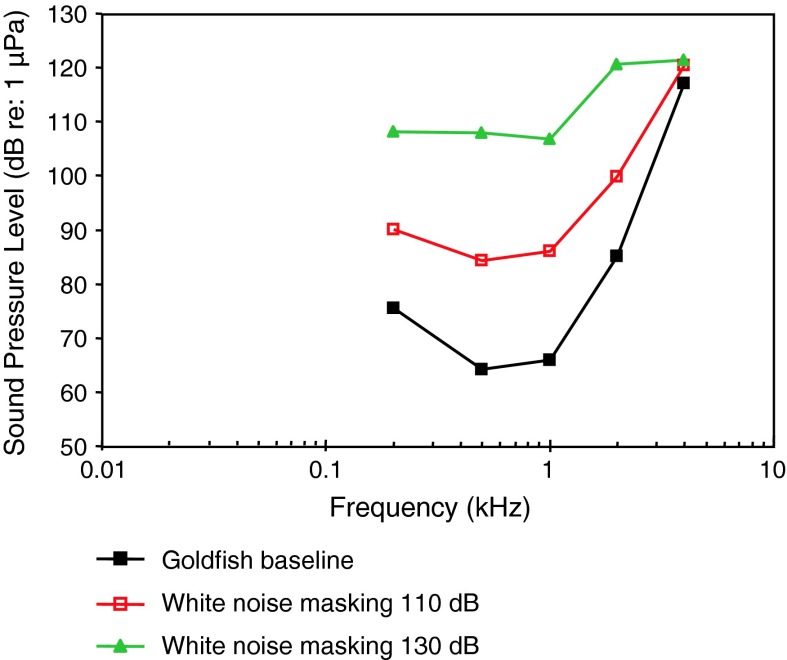
Hearing thresholds of the goldfish obtained under normal laboratory conditions (baseline) and in the presence of white masking noise of 110 and 130 dB. After Wysocki and Ladich (2005a)

**Fig. 55:**
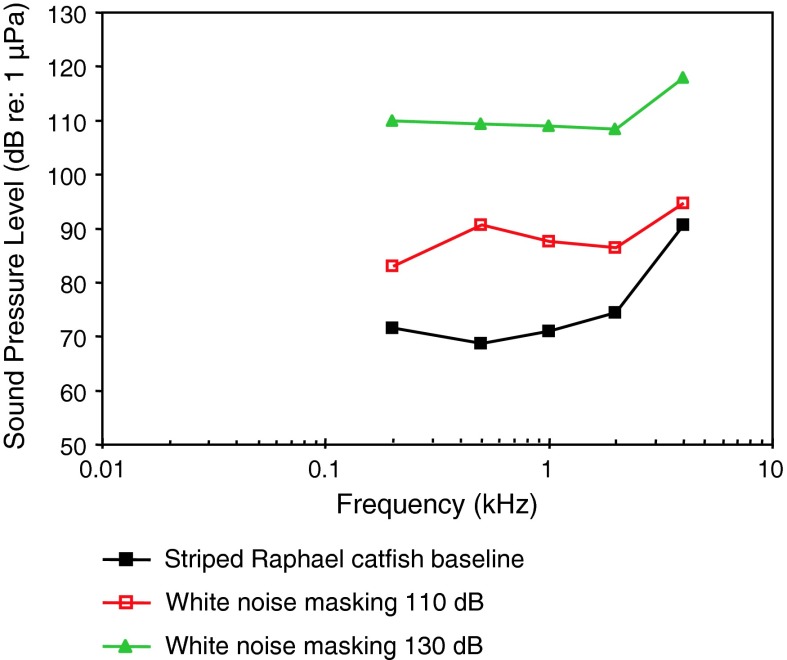
Hearing thresholds of the Striped Raphael catfish *Platydoras armatulus* obtained under normal laboratory conditions (baseline) and in the presence of white masking noise of 110 and 130 dB. After Wysocki and Ladich (2005a)

**Fig. 56:**
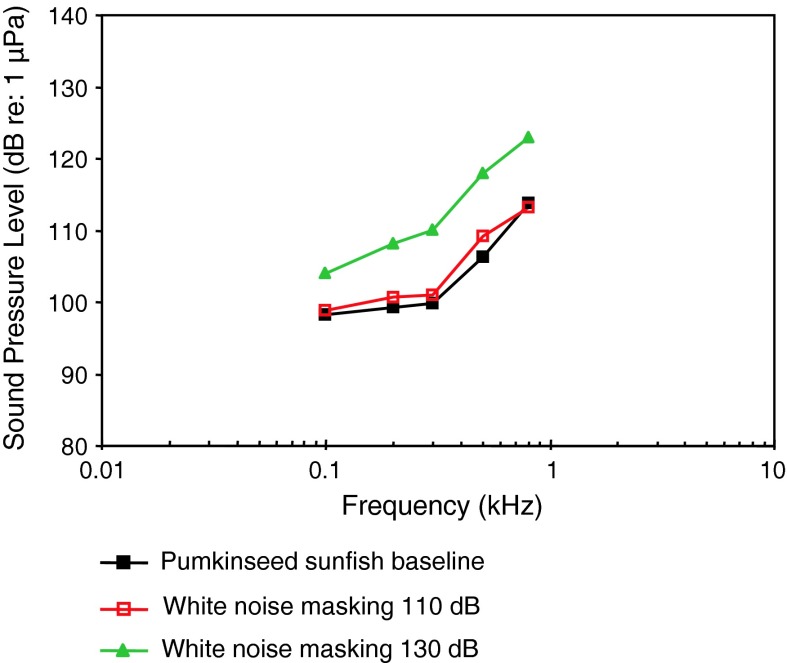
Hearing thresholds of the pumpkinseed sunfish *Lepomis gibbosus* obtained under normal laboratory conditions (baseline) and in the presence of white masking noise of 110 and 130 dB. After Wysocki and Ladich (2005a)

Wysocki and Ladich (2005a) investigated the effects of white noise on the hearing sensitivity in the goldfish, the striped Raphael catfish *Platydoras armatulus* (family Doradidae) and the pumpkinseed sunfish *Lepomis gibbosus* (family Centrarchidae) at two different SPLs of uniform spectrum noise (white noise). Continuous white noise of 110 dB RMS elevated the thresholds by up to 22 dB in the goldfish and in striped Raphael catfish. White noise of 130 dB RMS elevated overall hearing thresholds up to 44 dB in both otophysines (Figs. 54, 55). In contrast, auditory thresholds in the sunfish increased only at the higher noise level by up to 11 dB (Fig. 56).

In behavioral masking experiments, the signal-to-noise ratio at masked threshold (signal level in dB SPL at hearing threshold minus the white noise spectrum level in dB/Hz) is termed the critical masking ratio (CR) (Fay 1974; Fay 1988). When the CR using AEP is calculated for the goldfish, the CR function of frequency is not linear as is the behavioral CR function (and generally lower than the behavioral CRs) except at the lowest (200 Hz) and highest (2,000 Hz) frequencies tested. The lower CRs from the AEP studies indicate less masking than is evident in the behavioral experiments (Fay 1995; Tavolga 1974). Thus, the AEP method used to estimate CRs is not a good estimation of the behavioral CR, and cannot be interpreted in the same way.

**Fig. 57:**
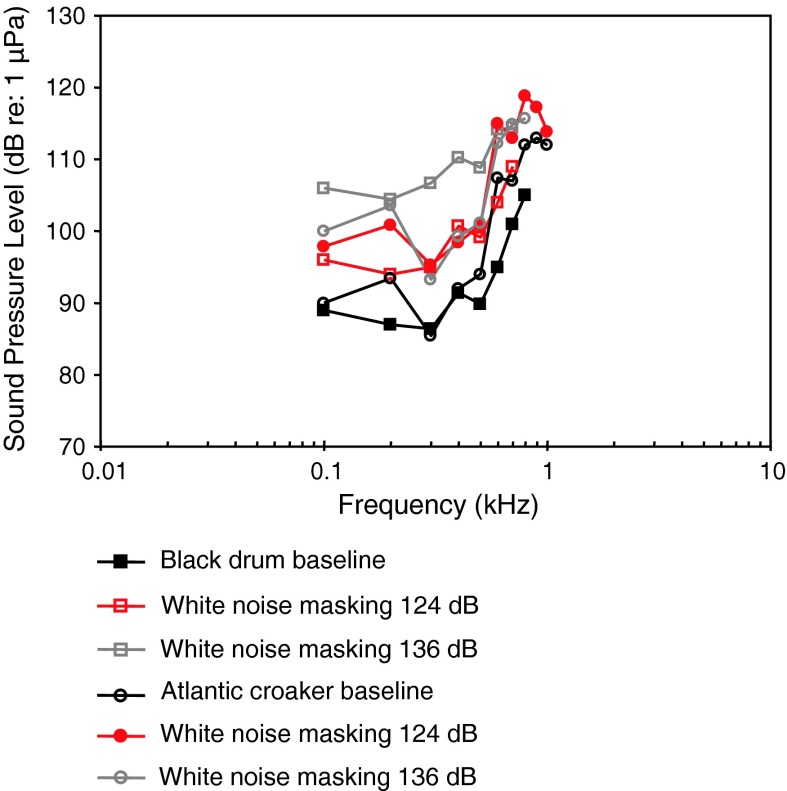
Hearing thresholds of the black drum *Pogonias chromis* and Atlantic croaker *Micropogonias undulatus* obtained under normal laboratory conditions (baseline) and in the presence of white masking noise of 124 and 136 dB. After Ramcharitar and Popper (2004)

Masking in the sunfish (Fig. 57) did not occur at 110 dB, probably because the sunfish is less sensitive overall than the goldfish and catfish, and is likely only sensitive to particle acceleration as well.

Ramcharitar and Popper (2004) measured the effect of masking by white noise in the black drum *Pogonias chromis* and Atlantic croaker (family Sciaenidae) to investigate the effect of different connections between the ear and swim bladder on noise masking effects. At the 124 dB level of white noise both species showed similar changes in auditory sensitivity. However, in the presence of the 136 dB white noise masker the black drum showed significantly greater shifts (to be expected) in auditory thresholds between 300 and 600 Hz, while the Atlantic croaker did not (Fig. 57).

### Ambient noise masking

Several studies were carried out to investigate if fish are adapted to the natural ambient noise levels or if signal detection is masked in particular regions of their habitat.

**Fig. 58:**
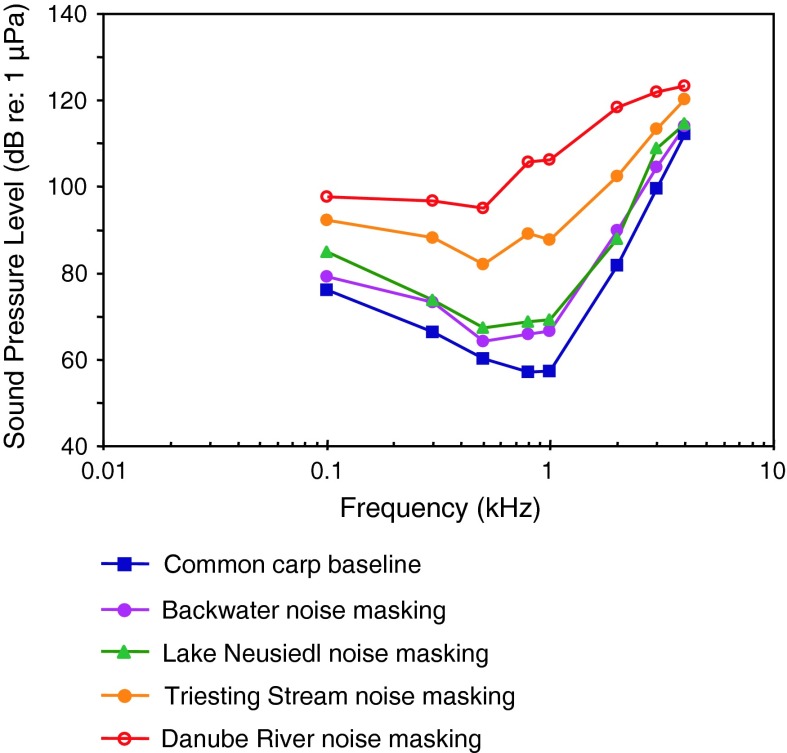
Hearing thresholds of the common carp *Cyprinus carpio* during quiet laboratory conditions (baseline) and in the presence of four different ambient masking noise conditions (Backwater: 92 dB; Lake Neusiedl: 93 dB; Triesting Stream: 114 dB; Danube River: 132 dB). From Amoser and Ladich (2005)

**Fig. 59:**
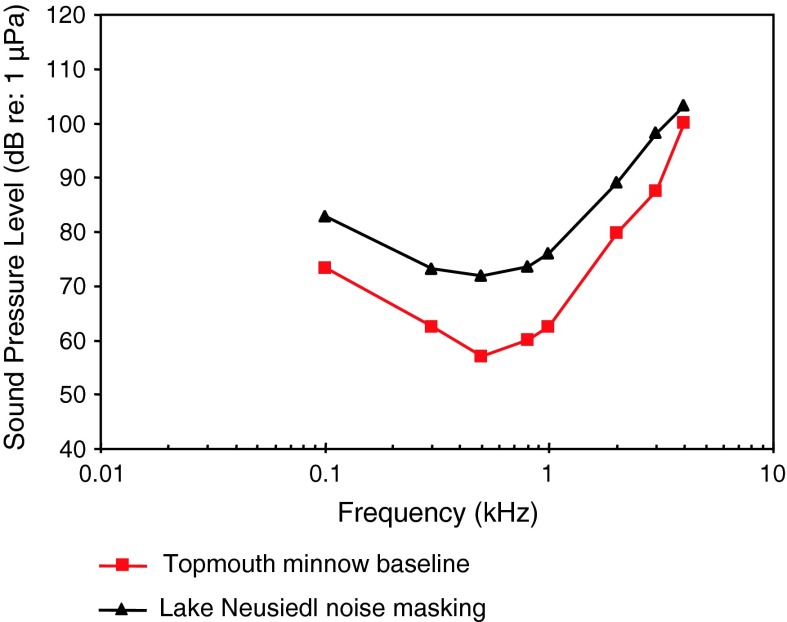
Hearing thresholds of the topmouth minnow *Pseudorasbora parva* during quiet laboratory conditions (baseline) and in the presence of ambient masking noise of Lake Neusiedl (93 dB). From Scholz and Ladich (2006)

Amoser and Ladich (2005) measured masked hearing thresholds of the common carp in the presence of ambient noise of four different habitats (Equivalent continuos SPL given for all habitats; Backwater: 92 dB; Lake Neusiedl: 93 dB; Triesting Stream: 114 dB; Danube River: 132 dB). The common carp’s hearing is heavily affected (masked) by stream and river noise by up to 49 dB (Fig. 58), and less so in a relatively quiet lake. Similar results were achieved when presenting lake noise to the closely related topmouth minnow (both family Cyprinidae) (Fig. 59) (Scholz and Ladich 2006).

**Fig. 60:**
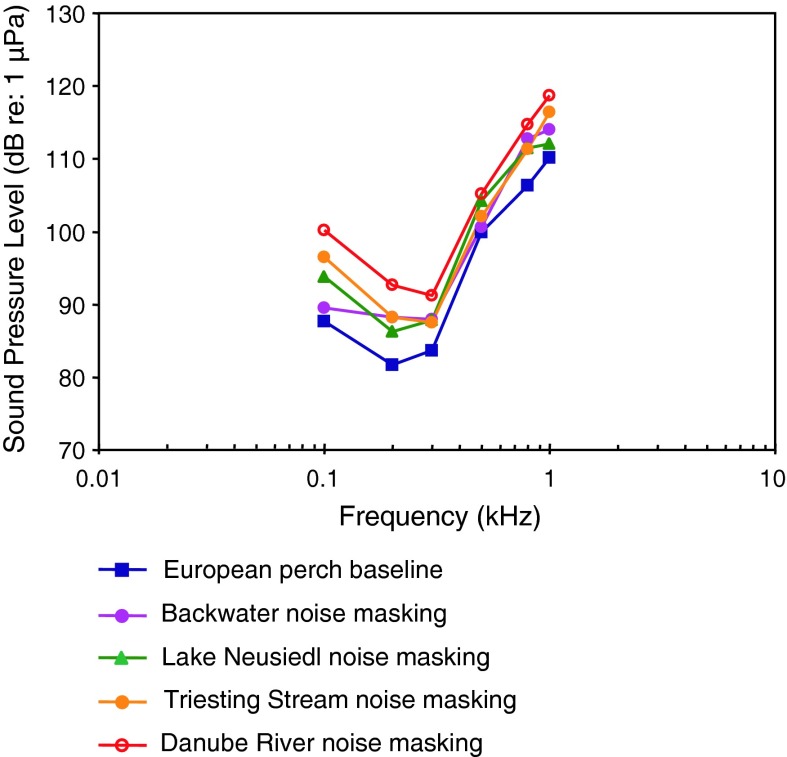
Hearing thresholds of European perch *Perca fluviatilis* during quiet laboratory conditions (baseline) and in the presence of four different ambient masking noise conditions (for details see Fig. 58). From Amoser and Ladich (2005)

Masking effects by ambient noise were much smaller in the European perch (family Percidae). The perch’s hearing thresholds were only slightly affected (mean up to 12 dB at 100 Hz) by the highest noise levels presented because it most likely lacks hearing specializations that would contribute to sound pressure sensitivity (Amoser and Ladich 2005) (Fig. 60).

**Fig. 61:**
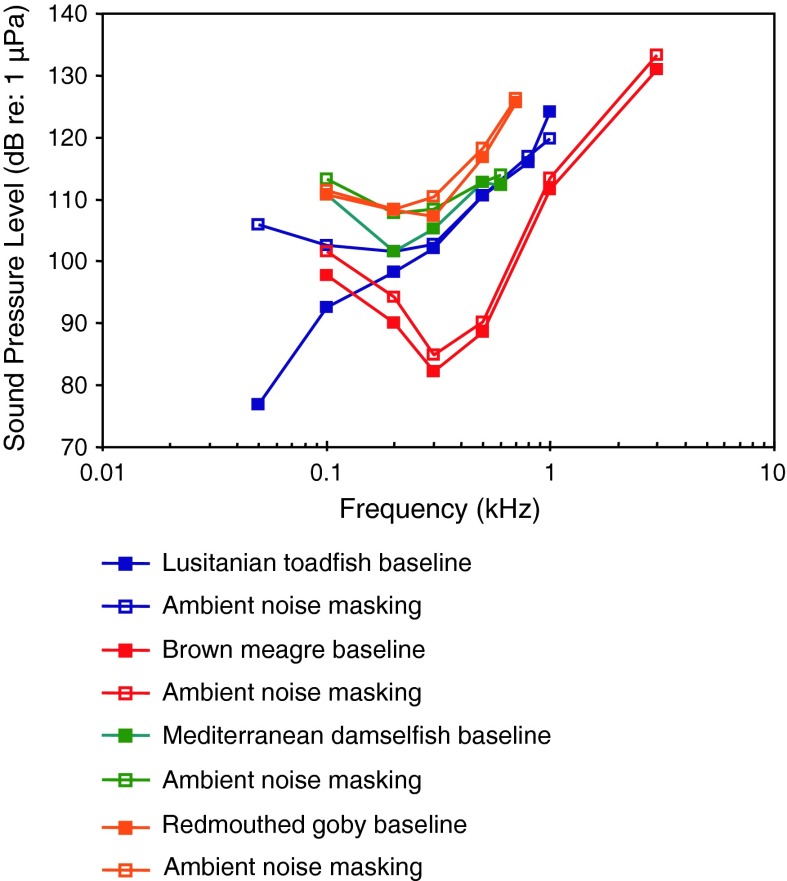
Hearing thresholds of in the Lusitanian toadfish *Halobatrachus didactylus*, the brown meagre *Sciaena umbra*, the Mediterranean damselfish *Chromis chromis* and the redmouthed goby *Gobius cruentatus* during quiet laboratory conditions (baseline) and in the presence of ambient masking noise conditions (111 dB for the toadfish and 97 dB for all other species) of their habitat. From Vasconcelos et al. (2007) and Codarin et al. (2009)

Masking by ambient noise was further studied in representatives of four marine families; the Lusitanian toadfish (family Batrachoididae), the brown meagre (family Sciaenidae), the Mediterranean damselfish (family Pomacentridae) and the redmouthed goby *Gobius cruentatus* (family Gobiidae). The ambient noise level was 111 dB for the toadfish and 97 dB for all other species. Data reveal that in all four species threshold shifts due to masking by ambient noise are small or insignificant, probably due to their adaptation to the ambient noise in their habitats (Vasconcelos et al. 2007, Codarin et al. 2009) (Fig. 61).

### Anthropogenic noise masking

Hearing in fish is frequently masked by anthropogenic noise either when kept for aquaculture or leisure or in the field by the presence of boat/ship noise, seismic air-gun shooting, various sonars, pile driving activity, and other sources.

**Fig. 62:**
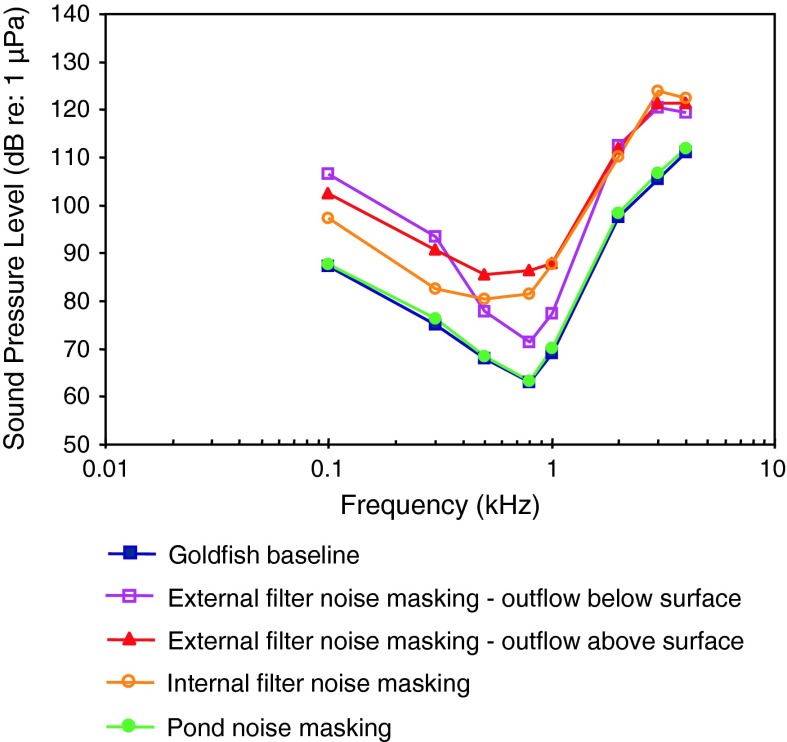
Hearing thresholds of the goldfish during quiet laboratory conditions (baseline) as well as in a pond and in the presence of masking noise generated by various aquarium filter setups. External filter, water outflow below surface: 115 dB; external filter, water outflow above surface: 119 dB; internal filter: 114 dB; pond noise: 95 dB. From Gutscher et al. (2011)

Gutscher et al. (2011) investigated the noise within a pond and of various aquarium filter setups and their effects on hearing in the goldfish (external filter, water outflow below surface: 115 dB; external filter, water outflow above surface: 119 dB; internal filter: 114 dB; pond noise: 95 dB). Pond noise did not affect hearing whereas noise caused by external aquarium filters resulted in a masked threshold shift of more than 20 dB in their best hearing range (Fig. 62).

**Fig. 63:**
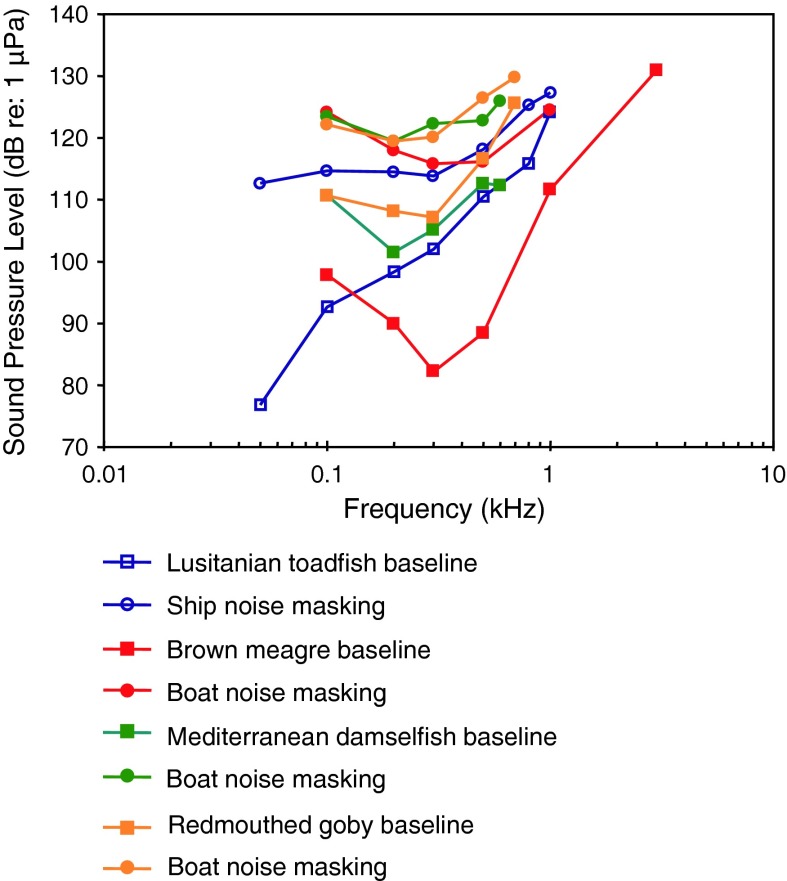
Hearing thresholds of the Lusitanian toadfish *Halobatrachus didactylus*, the brown meagre *Sciaena umbra*, the Mediterranean damselfish *Chromis chromis* and the redmouthed goby *Gobius cruentatus* during quiet laboratory conditions (baseline) and in the presence of ship and boat masking noise conditions of their habitat. Ship noise level was 131 dB for the toadfish and the boat noise level was 132 dB for the other species. From Vasconcelos et al. (2007) and Codarin et al. (2009)

Ship noise is a major anthropogenic noise source in aquatic habitats. The masking effects of ship noise have been studied in the Lusitanian toadfish, the brown meagre, the Mediterranean damselfish and the redmouthed goby. The ship noise level was 131 dB for the toadfish and 132 dB for the other species. Data reveal that the presence of ship noise decreases hearing sensitivities up to 40 dB and reduces the detectability of communication sounds (Vasconcelos et al. 2007, Codarin et al. 2009) (Fig. 63).

## Other factors affecting auditory sensitivity

A series of additional factors potentially affecting hearing sensitivity have been studied using the AEP technique. These include ecological (temperature, cave dwelling) and genetic factors (albinism), ototoxic drugs (gentamicin) and comparison of different AEP-recording protocols.

### Ecological factors

Fishes are ectothermic animals and their body temperature generally depends on ambient water temperature. Thus, ambient temperature might affect various physiological processes including sensory system function. Two studies applied the AEP-technique to find out if the auditory sensitivity changes with temperature in fishes. In general, an increase in sensitivity with frequency was found on all three catfish species investigated.

**Fig. 64:**
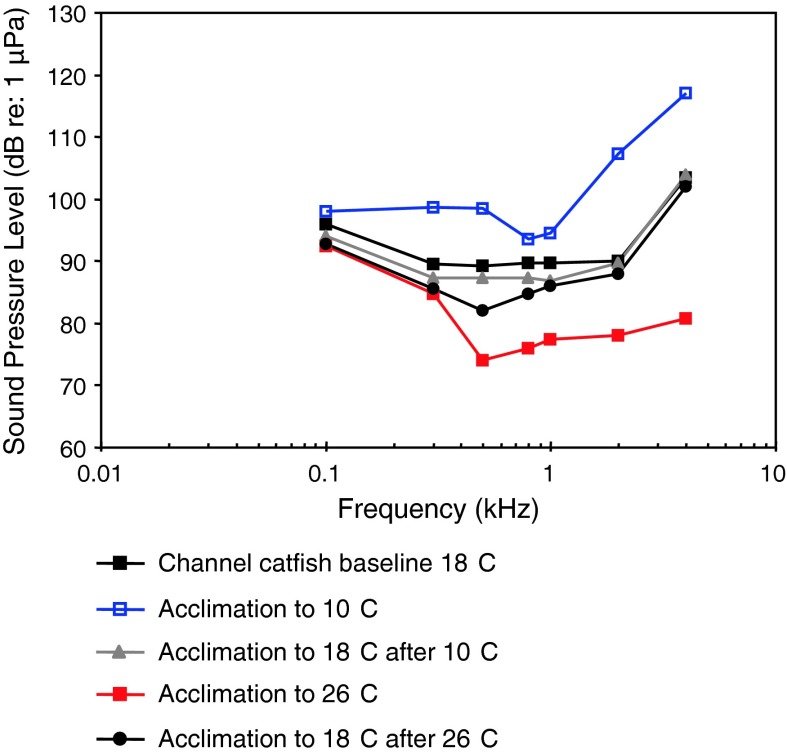
Hearing thresholds of the channel catfish *Ictalurus punctatus* at 18 °C (baseline) and after acclimation to 10, 18 and 26 °C after been acclimated to other temperatures. After Wysocki et al. (2009b)

Wysocki et al. (2009b) studied the sensitivity in the eurythermic channel catfish (family Ictaluridae) at 10, 18 and 26 °C and found that fish were up to 30 dB more sensitive at the highest temperature tested (Fig. 64). Acclimation to certain temperatures affected thresholds minimally.

**Fig. 65:**
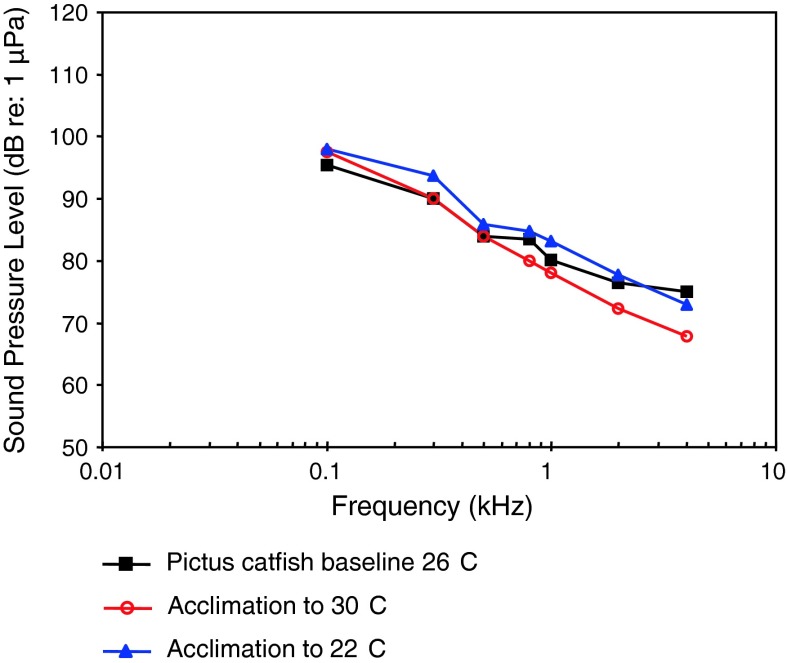
Hearing thresholds of the pictus cat *Pimelodus pictus* after acclimation to 26 °C (baseline), 22 and 30 °C. After Wysocki et al. (2009b)

**Fig. 66:**
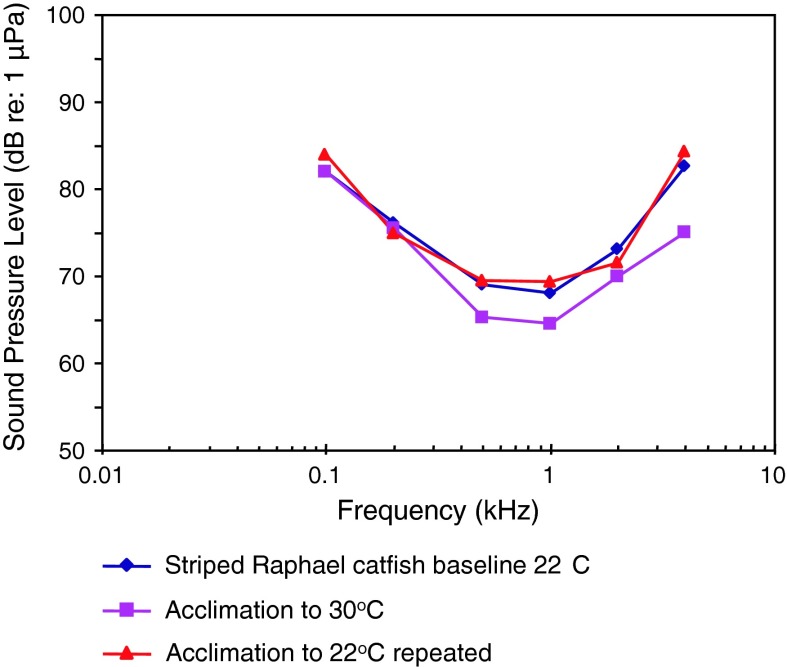
Hearing thresholds of the striped Raphael catfish *Platydoras armatulus* after acclimation to 22 °C (baseline), 30 °C and 22 °C. After Papes and Ladich (2011)

In contrast, the increase was much smaller in both Amazonian catfish species measured (stenothermic species). In the pictus cat (Pimelodidae) the hearing thresholds were maximally 7 dB higher at 30 °C as compared to 22 °C (Fig. 65) (Wysocki et al. 2009b). In the striped Raphael catfish a similar difference of up to 9 dB was observed after acclimating animals to both temperatures (Fig. 66) (Papes and Ladich 2011).

**Fig. 67:**
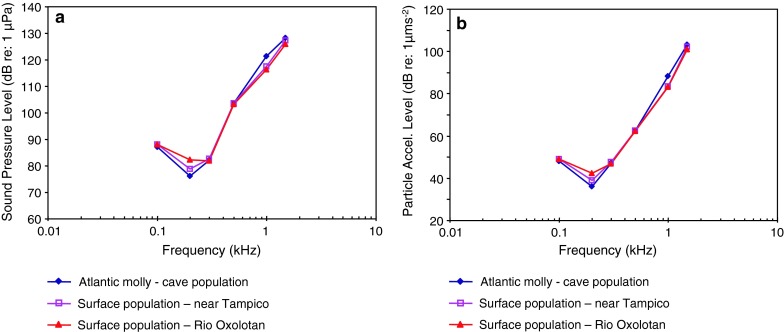
Hearing thresholds of a cave and two surface populations of the Atlantic molly *Poecilia mexicana.* **a** gives thresholds in terms of SPL and **b** in terms of PAL. After Schulz-Mirbach et al. (2010)

Besides temperature, lack of visual stimuli might potentially influence hearing in fishes. A comparison revealed similar auditory sensitivity in surface and cave dwelling (blind) populations of the Atlantic molly (Poeciliidae) (Schulz-Mirbach et al. 2010) (Fig. 67a, b).

### Genetic factors

**Fig. 68:**
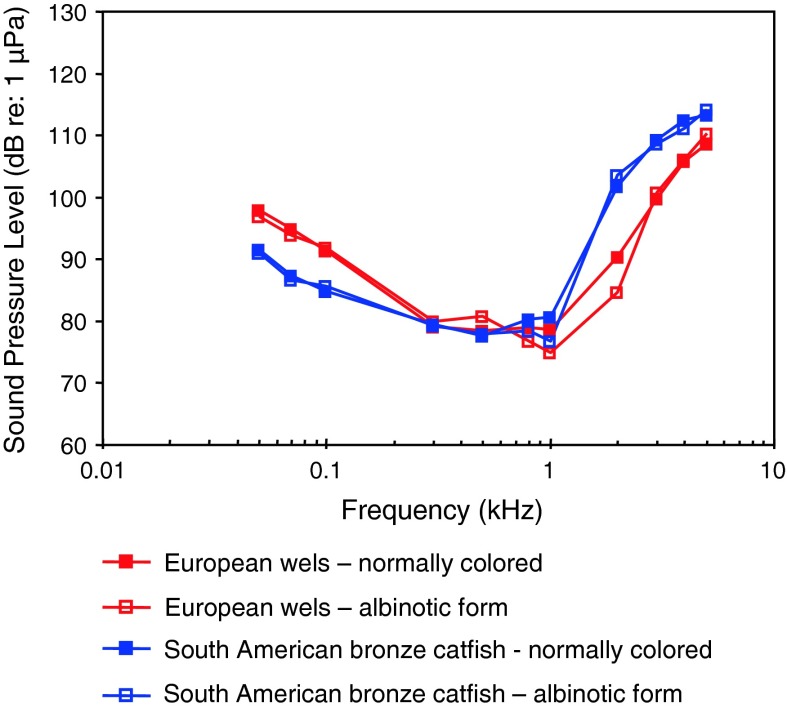
Hearing thresholds of normally colored and albinotic individuals of the European wels *Silurus glanis* and the South American bronze catfish *Corydoras aeneus*. After Lechner and Ladich (2011)

Albinism, a genetic abnormality of the melanin system in which the synthesis of this pigment is reduced or lacking, is occasionally associated with hearing impairments in mammals. Therefore, Lechner and Ladich (2011) compared auditory thresholds in normally pigmented and albinotic specimens of two catfish species, the European wels *Silurus glanis* (family Siluridae) and the South American bronze catfish *Corydoras aeneus* (family Callichthyidae). Auditory thresholds did not differ for either species between normally pigmented and albinotic specimens at any frequency tested (Fig. 68).

### Effects of ototoxins

**Fig. 69:**
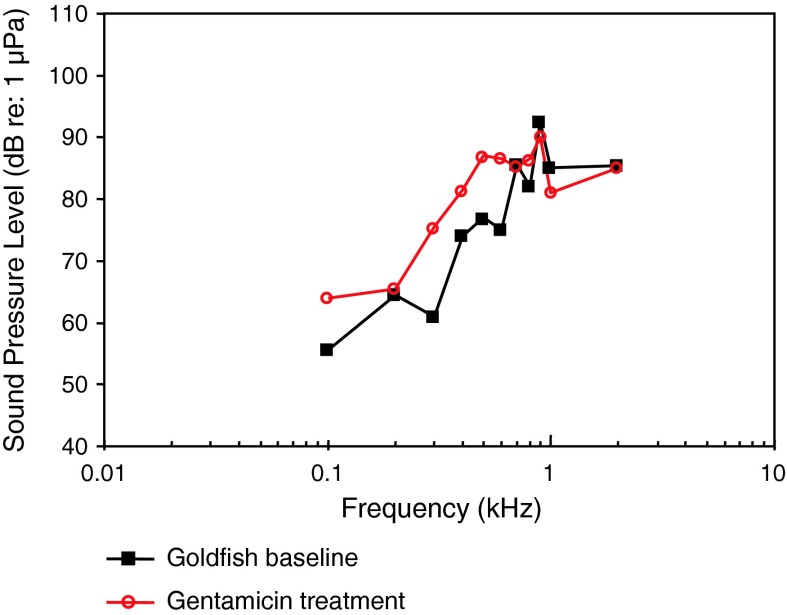
Hearing thresholds in goldfish without (baseline) and after administration of gentamicin at 100 mg/ml. After Ramcharitar and Brack (2010)

Antibiotics and other substances have been known to affect hearing sensitivity in vertebrates. Ramcharitar and Brack (2010) and Ramcharitar and Selckmann (2010) showed that gentamicin, a well studied human ototoxin, reduced hearing sensitivity in goldfish between 300 and 600 Hz (Fig. 69).

**Fig. 70:**
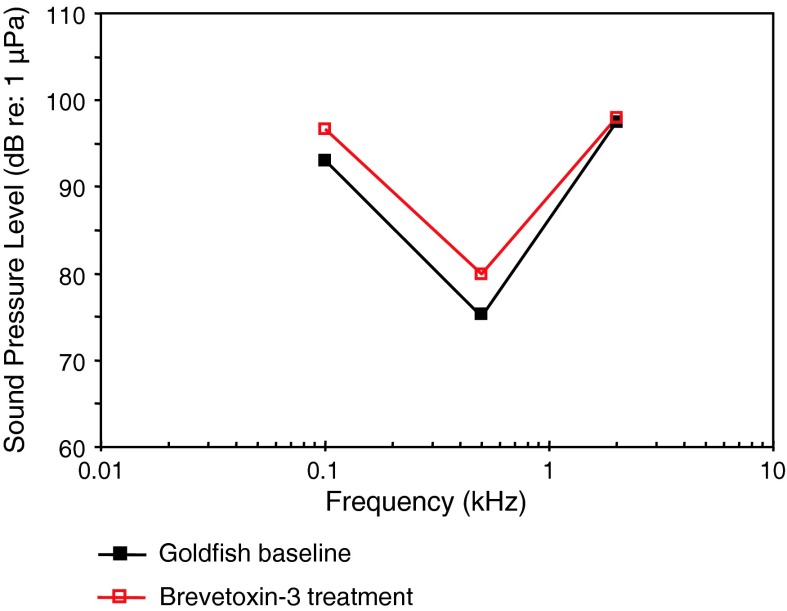
Hearing thresholds in goldfish without (baseline) and after administration of brevetoxin-3 (0.068 μg/g). After Lu and Tomchik (2002)

Lu and Tomchik (2002) studied the effect of the red-tide neurotoxin from dinoflagellates on hearing in goldfish. Sublethal-dose injection of brevetoxin-3 (0.068 μg/g) increased auditory thresholds up to 9 dB at low frequencies (100 and 500 Hz) (Fig. 70).

### Comparison of different AEP-protocols

**Fig. 71:**
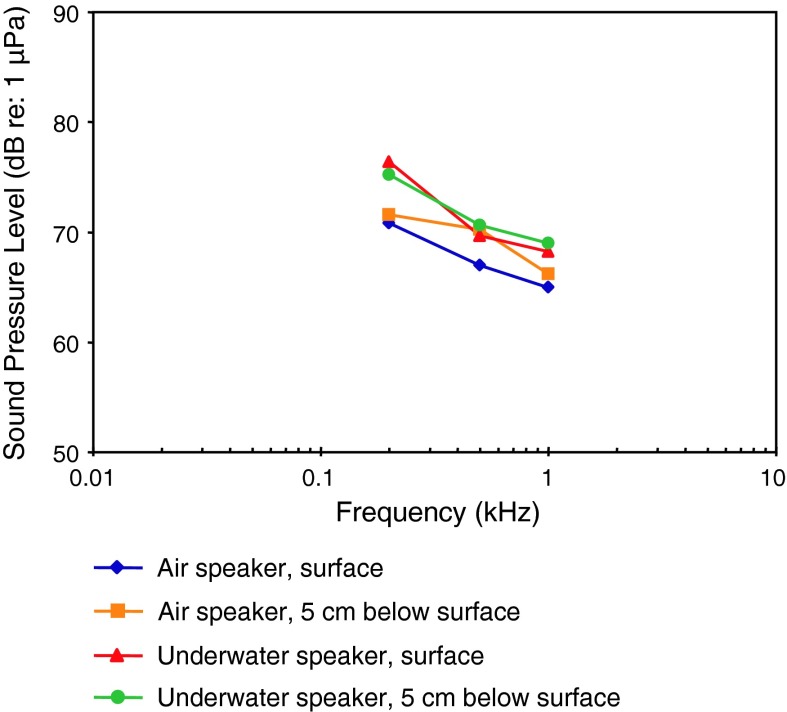
Hearing thresholds in goldfish at different positions in the sound field (at or 5 cm below surface) and when using different speakers (air speaker vs. underwater speaker). After Ladich and Wysocki (2009)

Auditory thresholds in the goldfish differ between different labs (see “Behavioral and electrophysiological measures of hearing function” section and Fig. 1). Ladich and Wysocki (2009) tried to find out if fish position or loudspeaker choice explains the variability in hearing thresholds in AEP-audiograms of goldfish. They determined hearing thresholds when fish where positioned at different water depths in the experimental tank (immediately below the water surface vs. 5 deeper) as well as when using different speakers (underwater speaker vs. speaker in air, above the water surface). They found that the maximum difference in hearing thresholds in different combinations of speakers and positions was 5.6 dB (Fig. 71). This rather small difference does not explain differences of more than 20 dB found at particular frequencies in different studies. Based on a survey of the literature, Ladich and Wysocki (2009) concluded that it is rather unlikely that factors such as fish size, temperature, background noise or degree of immobilization are responsible for difference in hearing threshold. The most likely reason (besides potential calibration errors) is the criterion stipulating what is regarded as an auditory threshold in AEP audiometry. Additional factors such as stimuli used, different numbers of AEPs averaged, signal-to-noise ratio in the electrophysiological recordings, and genetic differences between fish populations can add to the variation in hearing thresholds published for goldfish.

**Fig. 72:**
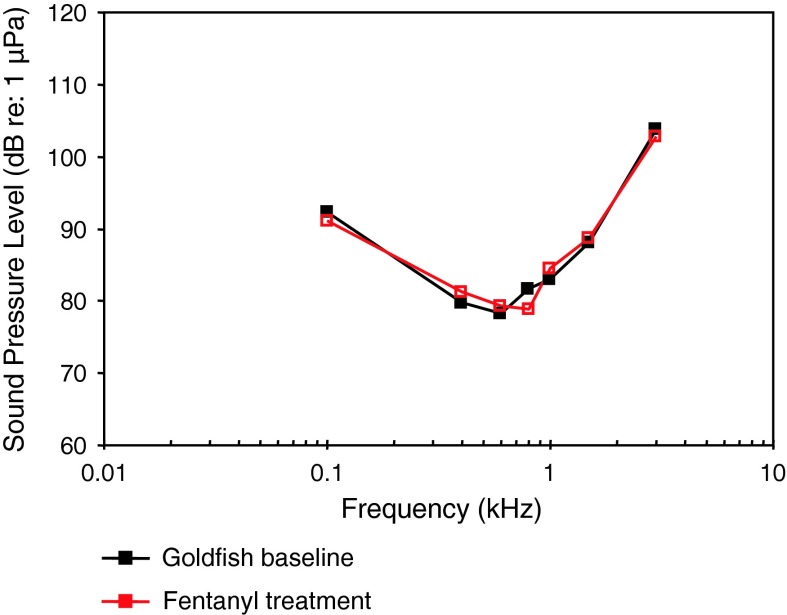
Hearing thresholds in goldfish after injections of flaxedil (baseline) and after fentanyl treatment. After Cordova and Braun (2007)

Cordova and Braun (2007) investigated if immobilization agents affect hearing thresholds. They compared auditory thresholds in goldfish after intramuscular injection of gallamine triethiodide (Flaxedil—a paralytic agent) or in combination with an injection of fentanyl (an anesthetic). Fentanyl (0.1, 0.5 and 2.5 mg g^−1^ fish) altered evoked potential waveforms slightly but did not alter estimated threshold sensitivity (Fig. 72).

Xiao and Braun (2008) investigated the effects of residual noise on threshold determination in order to reduce interobserver disagreements during subjective threshold estimations. An objective method of threshold determination was developed based on comparison between AEP amplitude and controlled residual noise.

### Effects of dominance and reproductive status

**Fig. 73:**
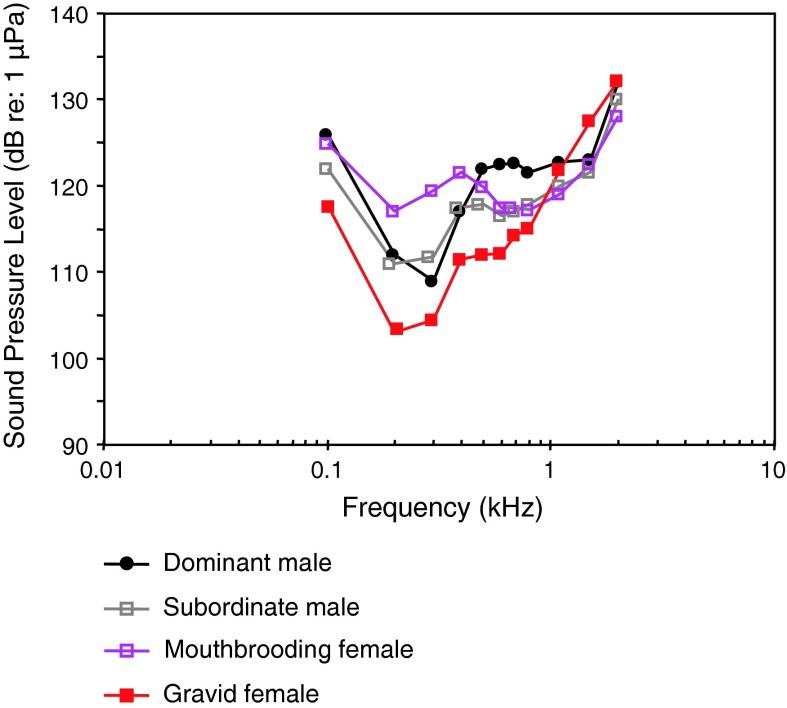
Hearing thresholds of the cichlid *Astatotilapia burtoni* depending on the dominance status in males (dominant, subordinate) and the reproductive status in females (gravid, mouth-brooding). After Maruska et al. (2012)

Maruska et al. (2012) found out that the dominance and reproductive status affects hearing in the social cichlid *Astatotilapia burtoni*. Subordinate males had lower thresholds than dominant males between 600 and 800 Hz (Fig. 73). In females, gravid individuals had lower thresholds (5–15 dB) at low frequencies from 100 to 600 Hz compared to mouth-brooding females.

## Applying AEP-techniques to study acoustic communication

The majority of AEP-studies is based on the determination of hearing thresholds in dB (either re 1 μPa or 1 μm/s^2^) gained under various conditions (see “Behavioral and electrophysiological measures of hearing function to Other factors affecting auditory sensitivity” sections). Hearing curves are frequently compared to the spectra of vocalizations to investigate the detectability of conspecific sounds in various contexts such as during ontogenetic development or in the presence of ambient or anthropogenic noise (Wysocki and Ladich 2001; Amoser and Ladich 2005; Vasconcelos et al. 2007; Maruska et al. 2012).

However, the technique is also suitable to study the temporal resolution of the auditory system. Temporal patterns of broad-band pulses within vocalizations are thought to be important carriers of information in fishes (e.g., Myrberg et al. 1978). In order to determine whether fishes are able to utilize temporal characteristics of acoustic signals Wysocki and Ladich (2002) determined the time resolution in four species of otophysines and anabantoids by analyzing AEPs gained in response to double-click stimuli with varying click inter-click intervals. The minimum interval resolvable by the auditory system using AEP methods was below 1.5 ms in each species studied (goldfish—Cyprinidae; striped Raphael catfish—Doradidae; croaking gourami, blue gourami—Osphronemidae) indicating the vocal species can process each pulse within intraspecific vocalizations. Wysocki and Ladich (2005b) studied the effects of white noise exposure (158 dB). Analysis of the response to double clicks showed that the minimum click period resolvable by the auditory system increased significantly from 1.25 to 2.08 ms immediately after noise exposure.

The AEP protocol was modified by Wysocki and Ladich (2003) to investigate how conspecific sounds are processed by the auditory system. AEPs elicited by conspecific sounds were recorded and analyzed in five species of teleosts. In fishes possessing sound pressure hearing specializations (striped Raphael catfish, pictus cat, orange finned loach, croaking gourami) each pulse within the sounds elicited a separate brainwave that closely followed the temporal structure of the stimulus. Data indicate that, besides temporal patterns, amplitude fluctuations and the frequency content of sounds can be represented in the auditory system to help extract important information for acoustic communication. In a subsequent study Codarin et al. (2009) determined the detectability of vocalizations by measuring the thresholds to conspecific sounds in the brown meagure and the Mediterranean damselfish. Vasconcelos et al. (2011) investigated the representation of conspecific mating and agonistic calls in the auditory system of the Lusitanian toadfish, and analysed auditory responses to vocalizations from heterospecifics such as the sympatric meagre *Argyrosomus regius* (Sciaenidae) and a potential predator (bottlenose dolphin *Tursiops truncatus*-family Delphinidae). The authors provide evidence that the auditory system of a vocal fish, lacking accessory hearing structures, is capable of resolving fine features of con- and heterospecific vocalizations.

## Summary and conclusions

In “Systematic description of baseline AEP-audiograms” section through “Applying AEP-techniques to study acoustic communication” we summarize in how many species and in how many different ways AEP techniques has been utilized to study hearing sensitivities in approximately 100 species of fishes. At least seven different methodical approaches have been used—ranging from the determination of baseline hearing sensitivities for various purposes up to the analysis of the AEPs gained in response to vocalizations. There are numerous advantages of the AEP techniques compared to behavioral conditioning techniques (see “Introduction” section). Nevertheless, it is necessary to summarize the advantages and shortcomings of the AEP technique.

One issue is the large variation of AEP audiograms for the same species, in particular the goldfish. This effect, however, is not peculiar to AEP studies since the same or more variation has been observed in behavioral experiments. When comparing the median goldfish audiograms from behavioral and AEP approaches we find a clear difference between these two methodical approaches. Based on the physics of the AEP technique we tentatively assume that the tendency seen in the goldfish, namely that the AEP technique gives higher thresholds at lower frequencies and lower thresholds at higher frequencies, reflects a general difference between techniques and can be expected for other species too. Because there is no second species which has been investigated as often as goldfish and because this goldfish trend cannot be seen as clearly in other species, it is impossible to quantify this effect and to derive a factor so that AEP audiograms can be transformed to closely match the behavioral data. Based on the goldfish comparison we assume that the ‘AEP effect’ is frequency dependent and that AEP curves can not simply be shifted downward to estimate the behavioral data.

In view of the large variation of AEP audiograms for the goldfish, one needs to be very cautious when comparing and interpreting results for individual species gained under different conditions or from different labs. The procedure that we recommend is to carry out all measurements under identical conditions (e.g., acoustical environment, threshold definition, etc.). Beyond that we recommend that every lab/beginner should measure goldfish for comparative purposes. This will give readers information on how thresholds measured in lab A potentially deviate from those gained in lab B.

When measurements are carried out under the identical conditions the AEP technique is a useful tool to measure and compare different species, different stages of age, and to determine the effects of accessory hearing structures, the effects of exposure to noise and the effects of masking by various noise types, the effects of temperature etc.

One generalization we can make from the data reviewed here is that all species known to possess potential specializations for sound pressure detection (a gas body near or in contact with the ears) have lower sound pressure thresholds at best frequency (55–83 dB), and respond at higher frequencies (200 Hz–3 kHz at best frequency) than fishes not known to be specialized. The fishes not known to be specialized are more diverse in sensitivity and frequency range, but generally have best thresholds between 78 and 150 dB, and best frequencies of below 100 to 1 kHz. All fishes studied by measuring AEP particle acceleration threshold levels have thresholds between 30 and 70 dB re: 1 μm s^−2^.

This review has identified several species, based on their AEP audiograms, that may have sound pressure sensitivity even though they have no obvious or known morphological specializations for detecting sound pressure. These include the red sea bream, the silver mojarra, the jewel cichlid, and the brown meagre, all perciformes.

All future audiometric studies on fishes, except species shown to be primarily sensitive to sound pressure, should include measurements of particle acceleration level in the test tank or test environment, and audiograms expressed in terms of particle acceleration as well as sound pressure. Ideally, the acoustic impedance (ratio of sound pressure to particle velocity levels) of the test environment should be characterized and compared to the impedance of the species natural habitat in order to evaluate the measured audiogram. Wherever possible, the lab studies should be carried out in tanks with very rigid walls (e.g., 4 cm-thick steel) to raise the impedance to near ideal levels (Halvorsen et al. 2011). Furthermore, more efforts should be made to experimentally determine the extent to which the species of interest is sensitive to both of these acoustic quantities. There are several ways to do this, including gas cavity extirpation (but note the caveat on using this procedure in the study by Yan et al. 2000, in Fig. 31 of “Using AEP-technique to investigate accessory hearing structures” section), and the estimation of thresholds as a function of distance from a sound source. If sound pressure thresholds vary with distance from the source, then it is likely that the animals are particle acceleration sensitive under those conditions, since particle acceleration declines steeply as distance increases near a sound source. If sound pressure thresholds are constant with variations in distance from the source, the animals are most likely pressure sensitive (e.g., Myrberg and Spires 1980). In any case, the measured sensitivity should be evaluated with respect to the actual impedance of the test environment, and its deviation from the natural habitat’s impedance. We are aware of the fact that suitable (ideally miniature) particle acceleration sensors for lab or field recordings are not (commercially) available and that fish bioacousticians face here a serious technical problem as compared to bioacousticians working with pressure sensitivity animals (crickets, frogs, birds or mammals) where suitable pressure sensitive equipment (e.g., microphones, sound level meters) is available.
